# Enteral nutrition modulation with n-3 PUFAs directs microbiome and lipid metabolism in mice

**DOI:** 10.1371/journal.pone.0248482

**Published:** 2021-03-25

**Authors:** Fuzheng Tao, Xi Xing, Jiannong Wu, Ronglin Jiang

**Affiliations:** 1 Intensive Care Unit, Taizhou Hospital of Integrated Traditional Chinese and Western Medicine, Taizhou, Zhejiang, China; 2 Intensive Care Unit, First Hospital Affiliated to Zhejiang University of Traditional Chinese Medicine, Hangzhou, Zhejiang, China; University of Pittsburgh, UNITED STATES

## Abstract

Nutritional support using exclusive enteral nutrition (EEN) has been studied as primary therapy for the management of liver diseases, Crohn’s disease, and cancers. EEN can also increase the number of beneficial microbiotas in the gut, improve bile acid and lipid metabolism, and decrease the number of harmful dietary micro-particles, possibly by influencing disease occurrence and increasing immunity. This study investigated the effects of EEN-n-3 polyunsaturated fatty acids (3PUFAs) (EEN-3PUFAs) on the gut microbiome, intestinal barrier, and lipid or bile acid metabolism in mice. Metagenomic sequencing technology was used to analyze the effects of EEN-3PUFAs on the composition of gut microbiome signatures. The contents of short-chain fatty acids (SCFAs) and bile acids in the feces and liver of the mice were assayed by gas chromatography and ultra-high-pressure liquid chromatography/high-resolution tandem mass spectrometry, respectively. The levels of lipopolysaccharide (LPS) and D-lactic acid in the blood were used to assess intestinal permeability. The results indicated that EEN-3PUFAs could improve the composition of gut microbiome signatures and increase the abundance of *Barnesiella* and *Lactobacillus* (genus), *Porphyromonadaceae*, and *Bacteroidia* (species), and *Bacteroidetes* (phylum) after EEN-3PUFAs initiation. In addition, EEN-3PUFAs induced the formation of SCFAs (mainly including acetic acid, propionic acid, and butyric acid) and increased the intestinal wall compared to the control group. In conclusion, EEN-3PUFAs modulate the alterations in gut microbiome signatures, enhanced intestinal barrier, and regulated the fatty acid composition and lipid metabolism shifts and the putative mechanisms underlying these effects.

## Introduction

Dysbiosis of the gut microbiota has been associated with multiple metabolic diseases, such as inflammatory bowel diseases (IBD) and Crohn’s disease (CD), cancer, hepatic steatosis, and type 2 diabetes mellitus [1–3]. The gut microbiome affects host lipid metabolism through multiple direct and indirect mechanisms [4]. Bäckhed *et al*. suggested that the gut microbiome is an important environmental factor that affects energy harvest from the diet and energy storage in the host [5]. Dysbiosis of the gut microbiome seriously influences the onset of multiple age- or endocrine-associated diseases and affects metabolic health. *E*. *coli* in the gut is positively correlated with the incidence of colorectal cancer, while *Lactobacilli* and *Bifidobacteria* can regulate the intestinal micro-ecology and reduce the risk of colorectal cancer [6]. Gut microbiome dysbiosis, e.g., a decrease in *Bifidobacteria* and other anaerobic beneficial bacteria and an increase of Gram-negative bacteria and other pathogenic bacteria, can easily induce intestinal metabolic disorders. A decrease of intestinal mucosal cells has been associated with an increase of the permeability and translocation of intestinal bacteria and various metabolites that release bacterial endotoxins into the blood and promote the production of a series of inflammatory factors with potential hepatotoxic effects [7,8]. Bäckhed *et al*. [5] found that disorders of the gut microbiome also affected the degradation of polysaccharides in food, leading to obesity and type I diabetes [9].

Among the major players in the intestinal microenvironment, bile acids (BAs) are synthesized from cholesterol in the liver and then stored in the gallbladder, from where they are secreted in the duodenal lumen upon food intake to facilitate fat digestion [10–12]. When they reach the colon, primary BAs are transformed by the microbiome to secondary BAs. This transformation is highly dependent on the microbiome composition and alterations, which are affected by the dietary intake, e.g., the content of fat and type of dietary fibers. Part of the secondary BAs is then reabsorbed, conjugated, and transported into the circulation. Studies have suggested that some BAs are involved in carbohydrate and lipid metabolism and that they can improve hyperglycemia [13], insulin resistance [14], intestinal inflammation [15], cholestasis disease [16], and gut barrier permeability, whereas others stimulate tumor growth [12] and colon cancer [17]. Hence, there is an urgent need to develop new methods for the identification and quantification of BAs in biological matrices.

Voitk *et al*. [18,19] first reported on the value of exclusive enteral nutrition (EEN) in the management of active IBD in the 1970s. Today, EENis recommended as first-line therapy for the treatment of active CD [20]. EEN has been shown to be beneficial for controlling disease, maintaining clinical remission, and addressing malnutrition in pediatric and adult patients with active CD [21]. In addition, there is no optimal formula that can achieve a maximal clinical response [22,23]. So far, wide ranges of formulations have been made commercially available, and decisions regarding formula choice are influenced by peripheral factors, such as patient comorbidities and volume status, or other complex factors [20,24]. EEN rich in n-3 polyunsaturated fatty acids (PUFAs) (fish oil) is widely used in clinical practice. EEN containing n-3 PUFAs have a certain beneficial effect when treating IBD and other diseases [25]. Furthermore, the addition of n-3 PUFAs to EEN can effectively reduce brain damage caused by transient cerebral ischemia in the middle cerebral artery and contribute to the recovery of neurological function after brain injury [26]. A previous study showed that n-3 PUFAs were beneficial to the recovery of respiratory function in patients with acute respiratory distress syndrome (ARDS) or ICU patients [27].

Overall, EEN, especially EEN rich in n-3 PUFAs, is an established therapy for inducing CD remission in the pediatric population; yet, its role as primary therapy for adult CD or other diseases (including critical patients) remains to be defined. In addition, the evidence confirming the accurate effects of n-3 PUFAs is limited, and the lack of evidence resulted in insignificant practice variations in the clinic. Therefore, the aim of this study was to develop and explore relevant assays to analyze gut microbiota alteration caused by EEN enriched in n-3 PUFAs on the lipid metabolism shifts with respect to lipid metabolism of n-3 PUFAs in the intestine and intestinal barrier.

## Materials and methods

### Study design

We conducted animal tests and animal control studies between November 2019 and January 2020 at the Animal Scientific Research Center of Zhejiang University, China.

All animal studies (including the mice euthanasia procedure) were done in compliance with the regulations and guidelines of Zhejiang University institutional animal care and conducted according to the AAALAC and the IACUC guidelines. Mice and related control data records were declared to the China data protection authority and registered at the China Experimental Test Registry. The study was approved by the Animal Care and Use Committee of Zhejiang University and IACUC.

### Animal grouping and processing

All animal studies (including the mouse euthanasia procedure) were done in compliance with the regulations and guidelines of Zhejiang University institutional animal care and conducted according to the AAALAC and IACUC guidelines. Mice and related control data records were declared to the China data protection authority and registered at the China Experimental Test Registry.

A total of 18 specific-pathogen-free BALB/c mice (nine males and nine females), weighing 18–20 g, were obtained from Shanghai Slack Co., Ltd, China. The mice were acclimatized for 1 week under controlled environmental conditions (temperature, 20–24°C; humidity, 40%-60%; light-dark cycle, 12–12 h) prior to the experiment. They were randomized according to three females and three males in the control group and three females and three males in two different treatment groups (two observational duration of treatment). The mice in the control group were given sterile water and were allowed to ingest food. The mice in the experimental groups were given 0.4 ml of EEN (Sino-Swed Pharmaceutical Corp. Ltd, China) on a daily basis [28]. The content of n-3 PUFAs in EEN was 1.5 g/500 ml. The two experimental groups were fed for 2 and 8 weeks, respectively, before mouse feces and blood samples collected. This was followed by blood samples taken from the eyes. The mice were euthanized by cervical dislocation and dissected upon completion of sample collection.

### Stool sample and fecal DNA extraction

Baseline fecal samples were collected 1 week prior to treatments and at 2 and 8 weeks after switching from the original polymeric diet to EEN rich in n-3 PUFAs. Two samples were collected at each time point to allow for separate analyses of bacterial DNA and metabolites. For sample collection, the subjects’ caregivers were supplied with sterile fecal sample tubes, freezer biohazard bags, instructions on sample collection, a Styrofoam cooler, and ice packs. Caregivers were instructed to collect a fecal sample within 24 h at each time point. After collection, the samples were immediately stored in a biohazard bag in -80°C freezers until further analysis. For the purpose of analysis, 300 mg of collected feces of individuals were mixed together in the same group and used for DNA extraction, high-throughput sequencing, and bioinformatics analysis. Metagenomic DNA was extracted from the mixed feces by the QIAamp DNA stool mini kit (Qiagen, USA) according to the manufacturer’s instructions. The extracted DNA was sub-packaged into four tubes to avoid multi-gelation before it was stored at 20°C.

### Analysis of gut microbiome signatures

High throughput sequencing and bioinformatics analyses were used to analyze the bacteria in the gut. Extracted intestinal macro-genomic DNA was segmented into the proper size for high-throughput sequencing. The DNA fragments were tied on a joint primer on both ends and connected to a flow cell by covalent bonds to achieve bridge amplification. Species classification was carried out on the processed sequences by the software RDP classifier (v2.10.1), which was based on Bergey’s taxonomy, adopting the Naive Bayesian assignment algorithm for each sequence to calculate p values to rank at different levels; the classification result was usually reliable (p > 0.8). Bergey’s taxonomy was divided into six layers: phylum, class, order, family, genus, and species. The dominant bacteria were mainly analyzed on the phylum, genus, and species levels. The approach to empirical research adopted for this study was one of qualitative, semi-structured high throughput sequencing and bioinformatics analyses of microorganisms.

### Determination of short-chain fatty acids in feces

For the analysis of the short-chain fatty acids (SCFAs), SCFAs were extracted from 60 mg of lyophilized stool samples. The samples were placed in a round-bottom flask and were gently suspended in 1.6 mL of distilled water. Subsequently, 0.4 mL of 50% aqueous H_2_SO_4_ and 2 ml of diethyl ether were added and mixed with an orbital shaker for 45 min, and then centrifuged at 3000 ×g for 5 min at room temperature. Anhydrous CaCl_2_ was mixed with the collected supernatant to remove the residual water. Then, 2 μL of supernatant was analyzed by gas chromatography, using an Agilent 7890A gas chromatograph fitted with a flame ionization detector (FID) and a GC column (ZB-FFAP, Phenomenex, America) of 30 m×0.32 mm×0.25 μm. Nitrogen was supplied as the carrier gas at a flow rate of 1.69 mL/min in non-split mode (injector temperature: 250°C). The initial oven temperature was 100°C for 2 min, which was then increased at a rate of 8°C**/**min to 240°C, and kept there for 10 min. SCFAs were quantified by a standard external method using the standard mix solution of acetic, propionic, butyric, and valeric acids.

### Determination of blood intestinal barrier index

The Pierce Chromogenic Endotoxin Quant Kit (Thermo Fisher Scientific, Waltham, MA, USA) and D-Lactate Assay Kit (Fluorometric, #ab174096, Abcam, Cambridge, UK) were used to analyze the levels of endotoxin and D-lactic acid, respectively, in the blood of the mice, according to the manufacturers’ instructions.

### Determination of bile acids (BAs) in feces

All BAs standards were purchased from Sigma (St. Louis, MO, USA). A standard stock solution of each free and conjugated BA was prepared at a concentration of 1 mg/mL in methanol. To be able to make solutions for calibration points, mixture solutions of all free BAs and conjugated BAs were prepared separately, at a concentration of 50 μg/mL in methanol. Stool samples, which were kept at -80°C, were allowed to thaw at room temperature. The samples were then accurately weighed (100 mg) and mixed with 200 μL of (NH_4_)_2_CO_3_. After 5 min, the samples were spiked with 500 μL of the internal standard precipitant solution (4°C, Sigma; 250μL of 80 M sodium cholate made by mixing 20μL of standard and 230μL of ultrapure water) and 500 μL of cold methanol. The samples were vortexed for 30 s and extracted for 2 min with ultrasound. After centrifugation at 13,000 ×g for 10 min at 4°C, the supernatant was collected and transferred to clean tubes and stored at -20°C. The residue was then reconstituted in 100 μL of ultrapure water and transferred into vials to be injected in the ultra-high-pressure liquid chromatography (UHPLC)-high-resolution tandem mass spectrometry (HRMS/MS) system. The stool samples were analyzed using a Waters Acquity UHPLC system (Waters Corporation, Milford, MA, USA). The samples were injected onto a Waters Acquity UHPLC BEHC_18_ column (1.8 μm, 2.1 × 100 mm; Waters Corporation). The temperature of the column was 40°C, and the flow rate was 0.4 mL/min. The mobile phases consisted of 10 mmol/L ammonium acetate in water (eluent A) and 0.1% formic acid in methanol (eluent B). The gradient elution was performed as follows: 35%-60% B (0–3 min) and 60%-100% B (3–4.5 min). At 4.5 min, the amount of B was kept constant at 100% for 0.5 min, followed by a quick drop to 35% for 1 min, and finally maintaining this concentration for 1 min for equilibration and column conditioning. The sample injection volume was 10 μL, and the auto-sampler temperature was 4°C. The Mass Spectrometry (MS) analysis was done using a Waters Xevo G2 QTOF (Waters MS Technologies, Manchester, UK) equipped with an electrospray ionization (ESI) source and operated in the positive ion mode. A capillary voltage of 2500 V, a sample cone voltage of 30 V, a source temperature of 150°C, and a desolation temperature of 450°C were applied. Data were collected in the centroid sensitivity mode in the range 100–800 *m/z*, with a lock spray scan collected every 30 s and an average of 3 scans to perform the mass correction. For quantification, the *m/z* of each molecular ion was used with a tolerance of 0.03 Da.

### Statistical analysis

Statistical significance between the control and EEN-treated groups was determined using a one-way analysis of variance (ANOVA). A P value <0.05 was considered statistically significant. Data analyses were performed using SPSS 13.0 (SPSS Inc., Chicago, IL, USA).

## Results and discussions

### Effects of EEN rich in n-3 PUFAs on mouse health

There were no differences in body weight between the experimental groups and the control group (P>0.05). At 2 and 8 weeks, the stool samples of all groups were brown, dry, and without diarrhea. The mice’s fur was smooth and shiny. All mice were in good health. No adverse health reactions were observed. These data suggested that EEN-3PUFAs did not affect mice growth.

### Comparisons of fecal bacterial communities in the treatment and control groups

**Fig 1:**
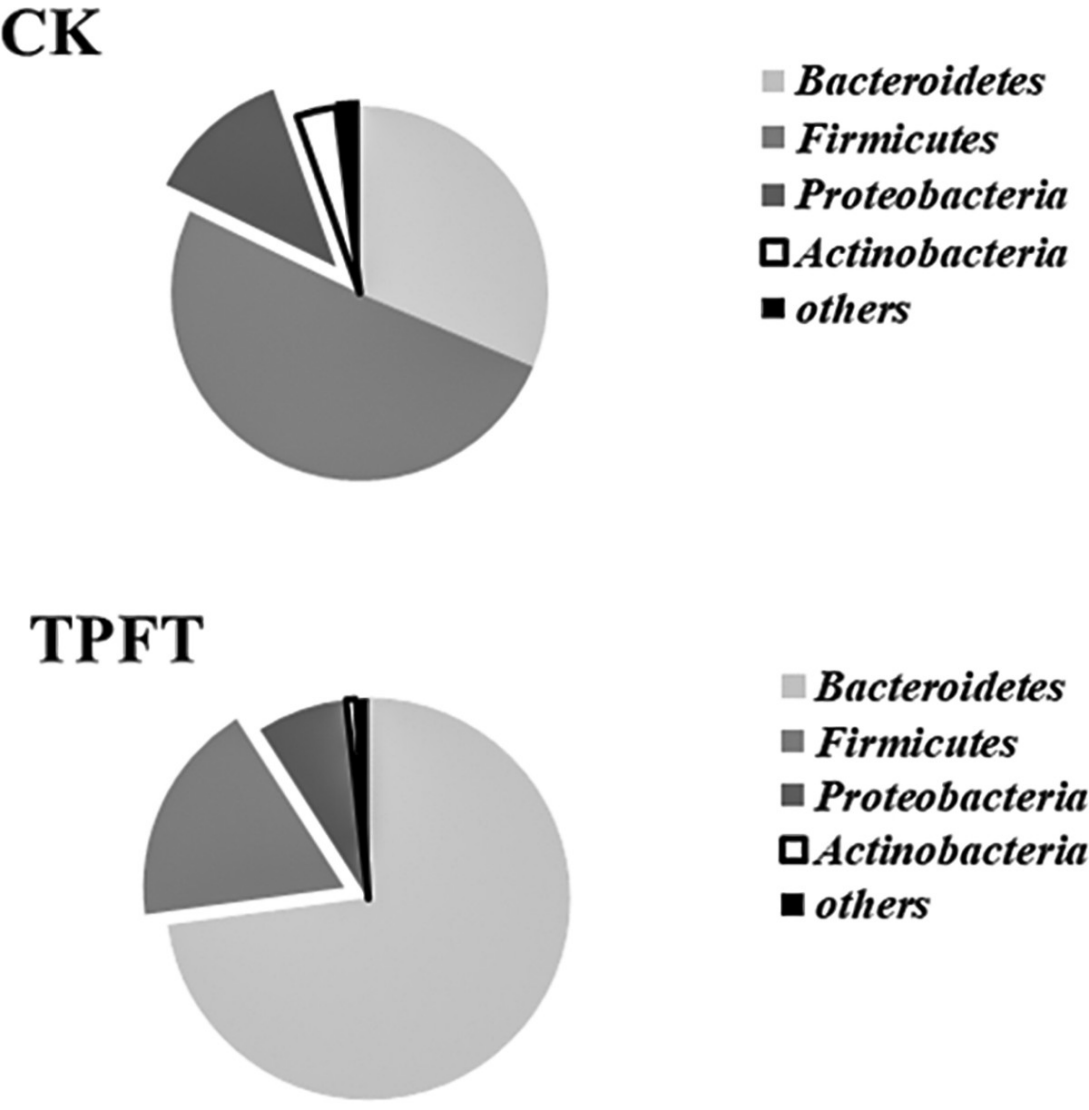
Effects of exclusive enteral nutrition (EEN)-n-3 polyunsaturated fatty acids (3PUFAs) on the gut microbiome (phylum level) after 8 weeks of treatment in mice compared with mice fed a regular diet. The bacteria were analyzed using the sequencing of the V4 region of the 16S ribosomal RNA. 3PUFAs-8 week: the experimental group treated with 3PUFAs for 8 weeks.

The bacteria taxa present within the fecal samples collected from the treatment and control groups are shown in **Fig 1**. α-diversity, a measure of species richness, was similar in the two groups.

In this experiment, we selected the specific-pathogen-free BALB/c mice as experimental animals because of their clear metabolic background and demonstrable fattening factor. To explore whether EEN-3PUFAs could induce modifications of the intestinal microbiota for the experimental durations, we quantified the highly abundant bacteria in the gut. Our data indicated that the fecal bacterial communities in control subjects clustered separately in the principal coordinate(s) analysis (PCoA) space compared to the treatment groups (p< 0.05). We used a linear discriminant analysis to compare the taxa present in the samples from the controls with taxa in baseline samples, i.e., before applying EEN-3PUFAs treatment. We found that *Firmicutes* were the most dominant bacteria in the gut, accounting for 50%-60% of the total bacterial population, followed by *Bacteroidetes*. *Proteobacteria* had a low abundance in the normal intestine, and their proportion was often less than 1%. *Actinomyces* is a type of strictly anaerobe Gram-positive bacterium. Although the number of bacteria in the gut was not dominant, *Bifidobacterium* was common in the intestine. Those results are supported by previous studies that reported those bacteria in the healthy gut [29–31].

*Streptomyces*, *Proteobacteria*, and *Putamen* were the most common phylum in the experimental mice fed with 0.4 ml EEN-3PUFAs. *Bacteroides* is a probiotic bacterium that helps the host to break down polysaccharides, increases the nutrient utilization rate [5], accelerates the formation of blood vessels in the intestinal mucosa [32], improves host immunity [33], and maintains the intestinal micro-ecological balance [34,35]. In particular, *Bacteroides* have a particularly prominent role in the utilization of polysaccharides [5]. As shown in **Fig 1**, the proportion of *Proteobacteria*, most of which are pathogenic bacteria that are associated with infectious and non-infectious IBD [36], was reduced in the treatment group, indicating the potential beneficial effects of EEN-3PUFA in the management of IBD.

**Fig 2:**
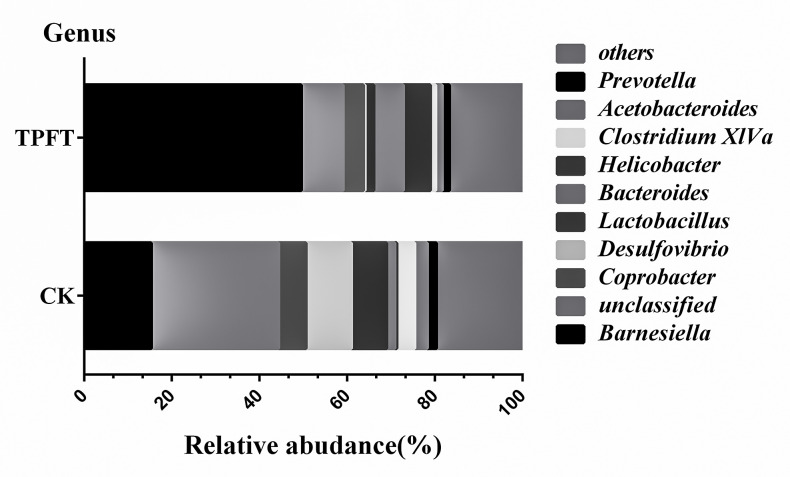
Effects of exclusive enteral nutrition (EEN)-n-3 polyunsaturated fatty acids (3PUFAs) on the gut microbiome (genus level) after 8 weeks of treatment in mice compared with mice fed a regular diet. The bacteria were analyzed at the genus level using the sequencing of the V4 region of the 16S ribosomal RNA. 3PUFAs-8 week: the experimental group treated with 3PUFAs for 8 weeks.

As shown in **Fig 2**, the relative abundance of gut microbiome distributions between the experimental and control groups was compared and analyzed at the genus level. The proportion of *Barnesiella* in the intestinal microbiome fed with EEN-3PUFAs increased significantly. *Barnesiella* can produce SCFAs in the gut and directly or indirectly act as an anti-inflammatory agent by increasing the content of SCFAs in the intestinal tract [37], protecting the intestinal barrier function [38], and regulating the role of lipid metabolism shifts and immunity [39]. It also plays an important role in the prevention and treatment of metabolic diseases, such as obesity, insulin resistance, and diabetes [39]. In addition, the amount of *Bacteroides* (3.64-fold that of the control group) and *Helicobacter* (15.8-fold that of the control group) increased, whereas *Desulfovibrio* (0.038-fold that of the control group) was greatly reduced in the experimental group. Although several species in the *Helicobacter* Genus (e.g. *H*. *pylori H*. *bizzozeronii*, *H*. *felis*, *H*. *salomonis*, *H*. *suis*, *and H*. *heilmannii*) have been known to associate with increased risk of gastritis, colorectal polyps, and especially the malignant diseases [40], a majority of other species have yet been functionally defined; therefore, their roles are still uncertain. In addition, accumulating evidence also suggests that *H*. *pylori*, as well as other species in the *Helicobacter* Genus (e.g. *H*. *mustelae*) may exert beneficial roles in obesity, childhood asthma, inflammatory bowel disease (IBD) and celiac disease among others [41–44]. As such, the result that *Helicobacter* (15.8-fold that of the control group) was increased is especially intriguing and the increased species remain to be further specified and functionally investigated in future studies.

**Fig 3:**
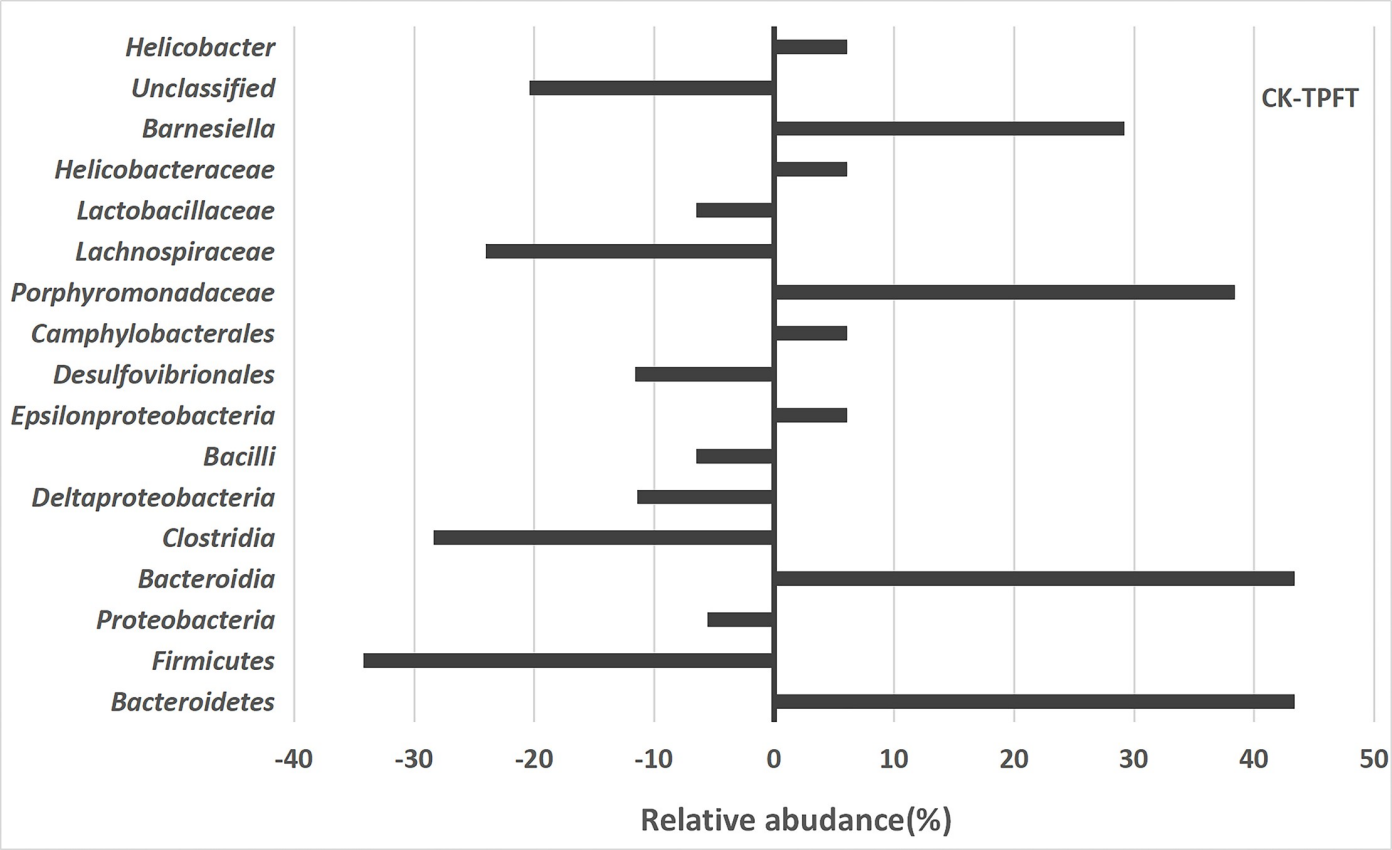
Effects of exclusive enteral nutrition (EEN)-n-3 polyunsaturated fatty acids (3PUFAs) on the gut microbiome (species level) after 8 weeks of treatment in mice compared with mice fed a regular diet. The bacteria were analyzed using the sequencing of the V4 region of the 16S ribosomal RNA. 3PUFAs-8 week: the experimental group treated with 3PUFAs for 8 weeks.

Guts containing pathogens at very high relative abundances (referred to as dominance) have a higher risk of developing bloodstream infections caused by that same dominant organism [45]. As shown in **Fig 3**, the relative abundance of gut microbiota in the 8-week treatment and control groups was compared in relation to the levels of phyla, class, order, and family. The abundance of *Lachnospiraceae* (*Clostridae*, *Firmicutes*), which are commonly found in a healthy colon, was higher in the control group than in the treatment group. The intestinal flora of the treatment group consisted mainly of *Barnesiella* (genus), *Porphyromonadaceae* (family), *Bacteroidia* (class), and *Bacteroidetes* (Phylum). *Bacteroidetes*, which are considered health-promoting bacteria, were distinguished by an increased abundance of common nosocomial pathogens, e.g., *Enterococcus* and the family *Enterobacteriaceae*. Again, this underlines a positive shift toward a more healthy gut microbiome using EEN-3PUFA.

When comparing control samples with the entire collection of study samples (i.e., all-time points), we found that the study samples were enriched with *Bacteroidetes* bacteria. Interestingly, neither of these taxa was dominant in samples collected after EEN initiation; they were replaced by dominant populations of *Bacteroides* in the mice. These results showed that the intestinal microbiota in the treatment groups underwent major changes. Despite the interindividual variations, we observed unambiguous EEN-3PUFAs-induced changes in the microbiota. Previously, Suzuki *et al*. [46] reported that *Firmicutes* bacteria had a certain effect on individual obesity. Hence, the reduction of Gram-positive bacteria could reduce the incidence of obesity. The *Desulfovibrio* was the more common pathogenic bacteria found in the control group. *Desulfovibrio* can reduce sulfates to sulfides in the intestine [47] and inhibit the oxidation of butyrate, thus leading to intestinal barrier dysfunction [48–51].

The content of *Lactobacillus* (Lac) in the feces of the experimental mice was significantly higher than that of the control group. *Lactobacillus* bacteria are non-toxic and harmless, rod-like bacteria, which can ferment carbohydrates and produce large quantities of lactic acid [52]. Among lactic acid bacteria, *Lactobacillus* was the largest genus, which was defined as rod-shaped *Lactobacilli*. These types of bacteria can prevent pathogens from invading and colonizing the intestine, inhibit pathogens, resist infections [53], maintain the micro-ecological balance of the intestine [54,55], enhance the body immunity, and prohibit the production of endotoxins. Therefore, the presence of Lactobacilli in the gut of the body is an important measure for the prevention and treatment of diseases. These data suggested that EEN-3PUFAs could increase the content of some probiotics, such as *Lactobacillus*, in the gut and improve the microbial environment.

As shown in **Fig 3**, the dominant bacteria of the treatment group with EEN-3PUFAs at the species level involved *Bacteroidetes*, *Bacteroidia*, *Porphyromonadaceae*, and *Barnesiella* compared with the control group. Moreover, *Firmicutes*, *Clostridia*, *Deltaproteobacteria*, *Bacilli*, *Desuifovibrionales*, *Lachnospiraceae*, *Lactobacillaceae*, and unclassified bacteria were all suppressed in abundance. These data showed that the diversity of the gut microbiome was significantly altered after EEN-3PUFAs was applied. Under normal conditions, the human body is relatively stable in its bacterial structure and does not cause any adverse manifestations to the host [56,57]. The proportion of beneficial bacteria in the intestine of healthy people and ordinary people is 70% and 25%, respectively. Dysbiosis in the gut can regulate lipid metabolism shifts, trigger low-grade chronic inflammation, and destroy the intestinal barrier [51] as a result of the alterations of the gut microbiome signatures. Furthermore, the gut microbiome regulates the mechanical barrier, immune barrier, and biological barrier of the intestinal mucosal system [58].

### The effect of EEN-3PUFAs on SCFAs (short-chain fatty acids) in mouse feces

SCFAs are products of unabsorbed dietary fibers that are mainly fermented by enteric bacteria in the colon [59]. Acetic acid, propionic acid, butyric acid, and valeric acid (or acetate, propionate, butyrate, and valerate) account for approximately 83% of the SCFAs produced [60]. Over recent years, studies showed that SCFAs had an enhanced effect on the immune function of the body [44] and intestinal function. SCFAs are absorbed and utilized by colon cells immediately after their formation and have an important protective effect on intestinal function [39,61].

**Fig 4:**
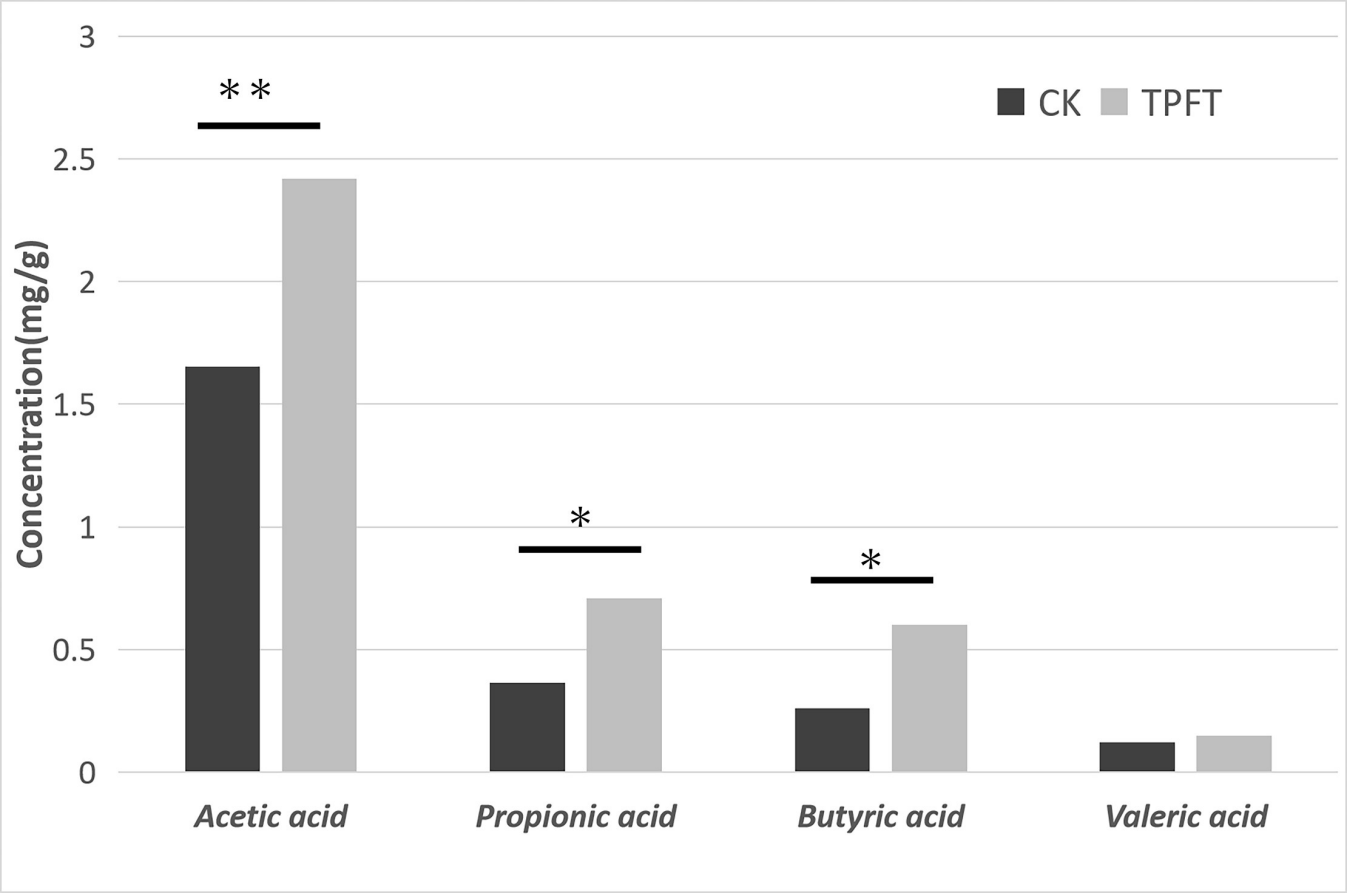
Effects of exclusive enteral nutrition (EEN)-n-3 polyunsaturated fatty acids (3PUFAs) on the content of short-chain fatty acids (SCFAs) after 8 weeks of treatment and compared with mice fed a regular diet. *, *P*<0.05; **, *P*<0.01. 3PUFAs-8 week: the experimental group treated with 3PUFAs for 8 weeks.

The concentrations of SCFAs within fecal samples collected before and after the EEN-3PUFAs intervention are shown in **Fig 4**. These measurements were done only on samples from the study mice. The concentration of acetic, propionic, and butyric acid, key metabolites in fermentative metabolism, were significantly higher in the 8-week treatment samples than in the control groups. Moreover, compared with the control group, the content of SCFAs (mainly including acetic acid, propionic acid, and butyric acid) in the feces of the experimental mice were significantly increased to varying degrees (1.45-fold, 1.95-fold, and 2.25-fold, respectively; all P<0.05), thus suggesting that the intake of EEN-3PUFAs might increase the levels of intestinal SCFAs in mice.

Part of the SCFAs are not oxidized by the colonic mucosal cells, and fatty acids can be converted into glutamine and ketone bodies (acetoacetic acid and beta-hydroxybutyric acid, etc.) through the portal vein and entering the systemic circulation together with some non-liver-transformed SCFAs, eventually reaching the intestines [62]. These substances are an important source of energy in the small intestine mucosa [63]. The increase of the levels of SCFAs in the feces was mainly caused by changes in the gut microbiome, which were related to the intake of EEN-3PUFAs. Besides, the amount of SCFAs produced by probiotics can maintain the normal function of the intestinal mucosal cells and has a protective effect on the intestinal mucosal barrier function.

### The effect of EEN-3PUFAs on intestinal barrier

**Fig 5:**
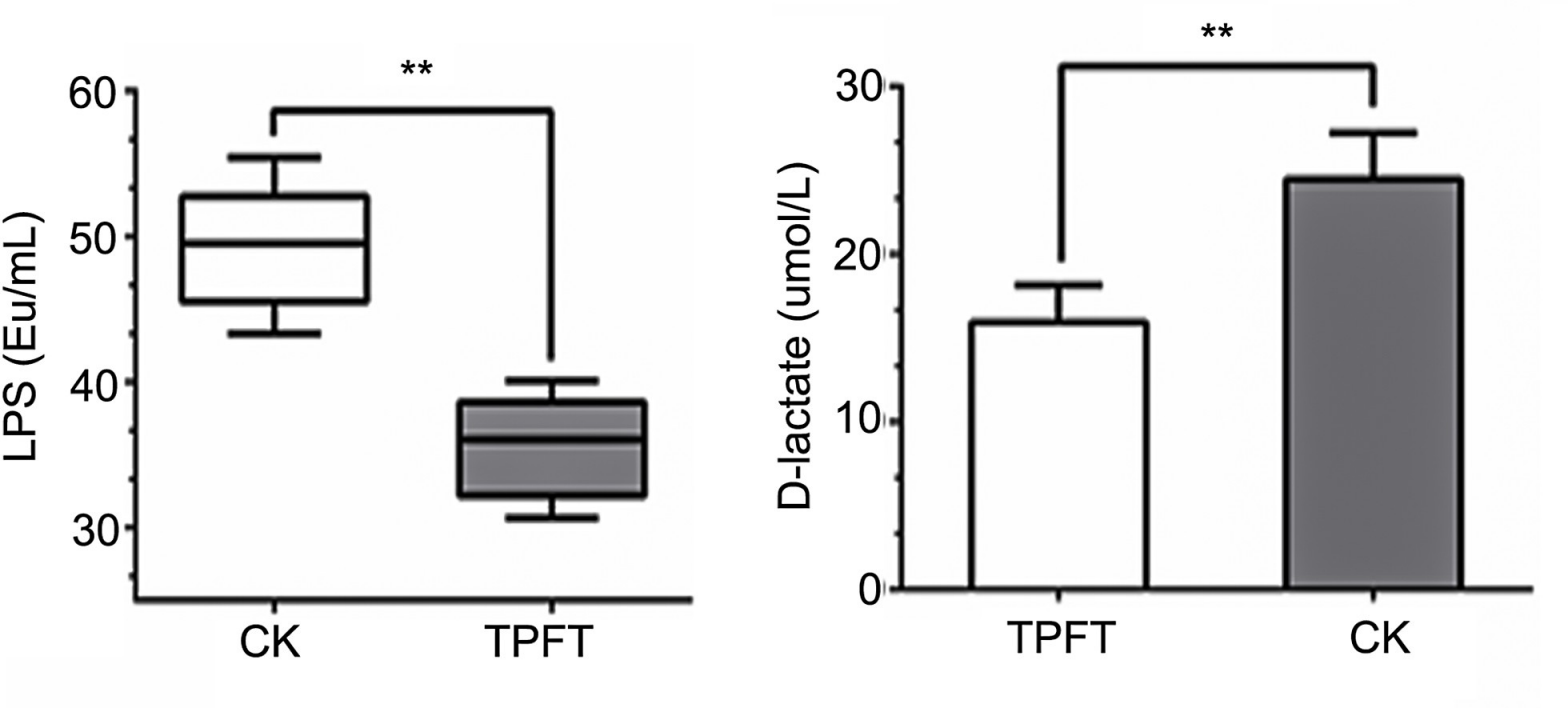
The effect of exclusive enteral nutrition (EEN)-n-3 polyunsaturated fatty acids (3PUFAs) on intestinal barrier functions 8 weeks after treatment based on lipopolysaccharides and D-lactic acid levels. **, *P*<0.01. 3PUFAs-8 week: The experimental group treated with 3PUFAs for 8 weeks.

The intestinal mucosal barrier prevents harmful substances, such as bacteria and toxins, from passing through the mucosa and affect other tissues and organs of the body [64]. The normal intestinal mucosal barrier consists of a biochemical, mechanical, and immune barrier [64,65]. Intestinal epithelial cells form a selective barrier between desmosomes, adherent junctions, and tight junctions and prevent the entry of potentially harmful substances [60,61]. However, when the intestinal mucosal barrier homeostasis is disrupted, intestinal epithelial permeability increases, enabling bacteria translocation, which can lead to systemic inflammation [66]. Endotoxins are Lipopolysaccharides (LPS) in the cell wall of Gram-negative bacteria that are decomposed and released during bacterial metabolism or death. When the intestinal barrier is damaged and mucous membrane permeability increased, LPS enters the circulatory system through the intestinal mucosa [64,65]. When the portal vein blood LPS concentration is increased, the liver Kupffer cells are stimulated to release a series of cytokines, such as tumor necrosis factor (TNF), IL1, IL6, free radicals, etc., thus causing damage to the whole body and multiple organs [67]. As shown in **Fig 5**, the contents of LPS were significantly reduced in the treatment group, which suggested the beneficial effect of the EEN-3PUFAs on the intestinal barrier. Supplement of nutrition-rich in n-3 PUFAs was beneficial to maintaining the integrity of the intestinal barrier through altering gut microbiome signatures.

D-lactic acid is a metabolite produced by bacterial fermentation [68]. Many kinds of bacteria in the intestine can produce D-lactic acid [68]. When the intestinal barrier function is impaired, a large amount of D-lactic acid in the intestine can enter the blood circulation system through the damaged intestinal mucosa [68]. Therefore, monitoring D-lactate levels in the blood can promptly reflect the changes in intestinal mucosal damage and permeability. Animal experiments showed that the intestinal mucosal damage caused by acute intestinal ischemia in rats could rapidly increase blood D-lactate levels [69]. With the prolongation of ischemic time and increasing D-lactic acid concentration, the degree of intestinal barrier injury was aggravated. The blood D-lactate content is significantly and positively correlated with intestinal mucosal injury score and blood endotoxin levels, thus suggesting that blood D-lactate levels can be used as a functional state reflecting the mechanical barrier in the intestinal barrier and as an important indicator of endotoxin and bacterial translocation in the intestine [70]. As shown in **Fig 5,** the levels of D-lactic acid in the blood of the treatment group were lower than those in the control group, indicating that EEN-3PUFAs can enhance the intestinal barrier function in mice.

### Change in levels of bile acids (BAs) in response to EEN-3PUFAs

Studies have suggested that BAs involved in carbohydrate and lipid metabolism may improve hyperglycemia [13], insulin resistance [14], intestinal inflammation [15], cholestasis disease [16], and gut barrier permeability, but can also stimulate tumor development [12,17]. Up to 90% of the primary BAs in human adults are produced through the classical neutral pathway in the liver, while the remaining 10% are synthesized via the acidic pathway [71]. In humans, the BA pool size is kept comparatively stable at about 3–5 g through the enterohepatic circulation [72]. During this process, about 95% of the BAs that have entered the small intestine are reabsorbed from the terminal ileum back to the liver via the portal vein [11]. The remaining BAs enter the large intestine, where they undergo an extensive bacterial transformation before absorption/excretion [10,73]. Previous studies have shown that dietary factors can affect atherogenesis in mice by microbial modulation of BA synthesis in the liver [74,75]. Both lingo berries and resveratrol increase the numbers of *Lactobacillus*, *Bifidobacterium*, and *Akkermansia* in the gut, and *Lactobacillus* and *Bifidobacterium* possess bile-salt hydrolase [76].

**Fig 6:**
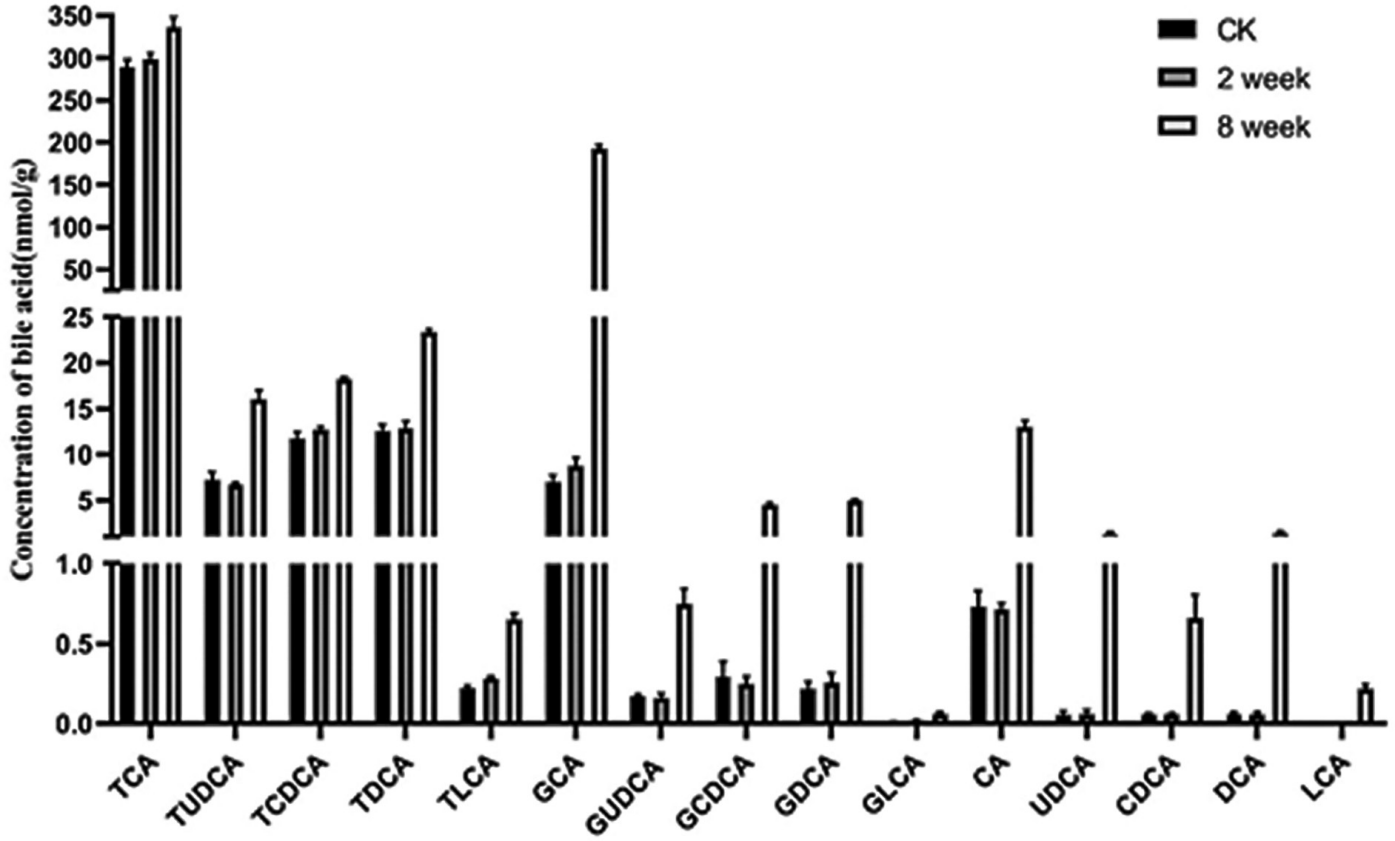
Contents of bile acids in the liver of mice after 2 and 8 weeks of treatment with exclusive enteral nutrition (EEN)-n-3 polyunsaturated fatty acids (3PUFAs) compared with mice fed a regular diet. 3PUFAs-2 week: the experimental group treated with 3PUFAs for 2 weeks; 3PUFAs-8 week: the experimental group treated with 3PUFAs for 8 weeks.

**Fig 7:**
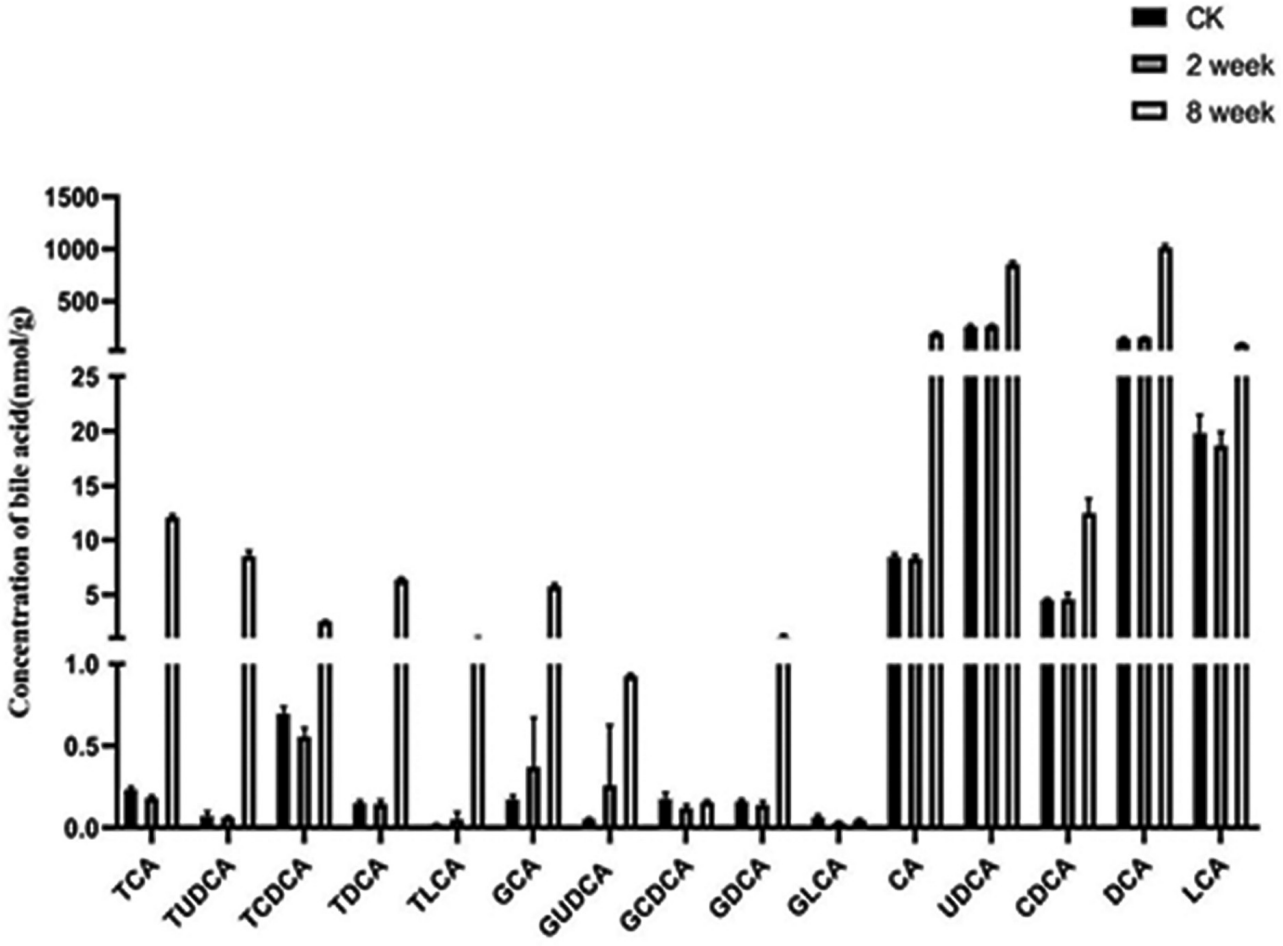
Contents of bile acids in feces of mice in the treatment for 2 and 8 weeks of treatment with exclusive enteral nutrition (EEN)-n-3 polyunsaturated fatty acids (3PUFAs) compared with mice fed a regular diet. 3PUFAs-2 week: the experimental group treated with 3PUFAs for 2 weeks; 3PUFAs-8 week: the experimental group treated with 3PUFAs for 8 weeks.

**Fig 8:**
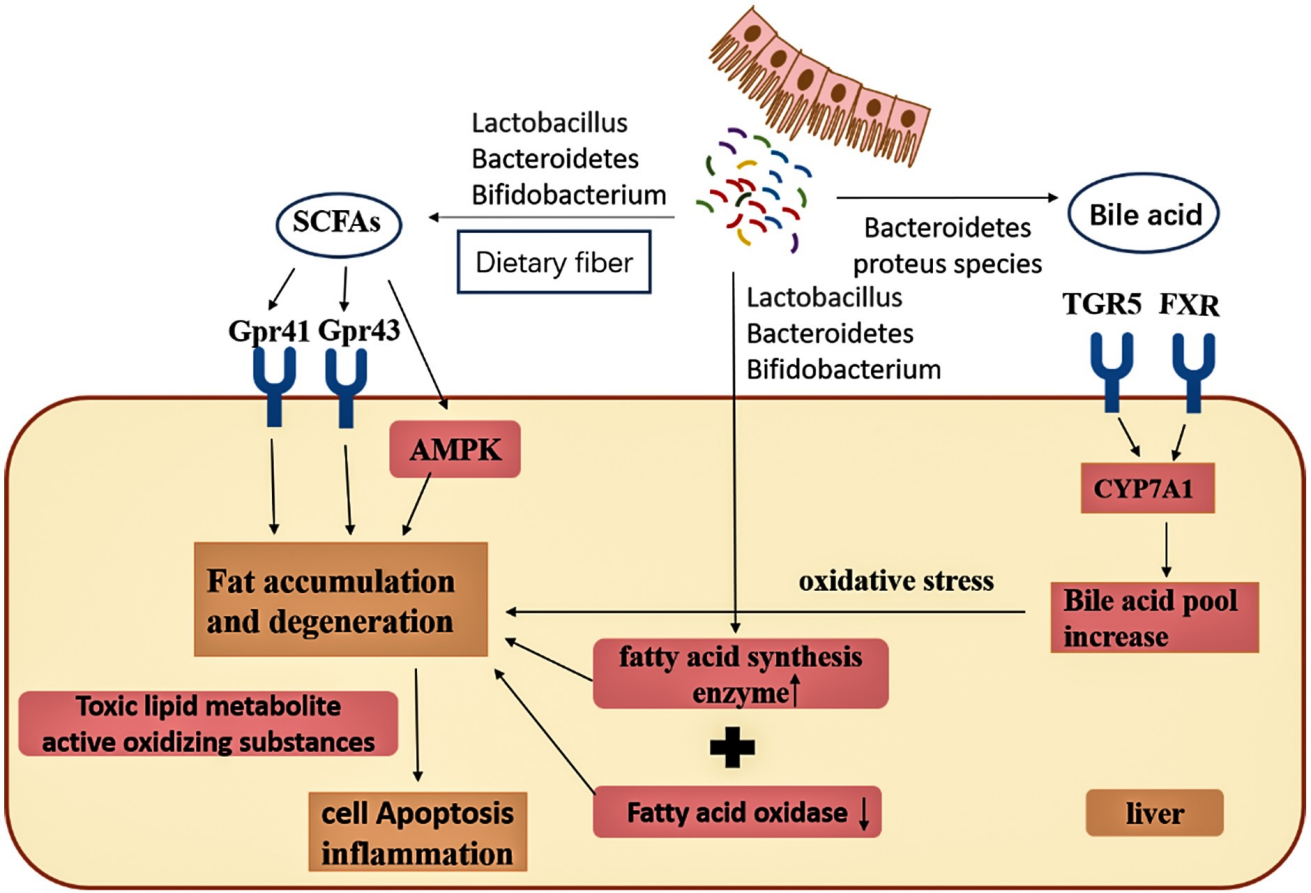
Summary of the pathways for interactions of short-chain fatty acids (SCFAs), bile acids, and the gut microbiome.

As indicated in **Figs 6** and **7**, we found that the concentration of most unconjugated BAs was higher (p < 0.001) in the liver of mice fed with EEN-3PUFAs for 2 or 8 weeks compared with the control group. The liver concentration of some free BAs increased further and was significant (p < 0.01 and p < 0.0001) for cholic acid (CA), ursodeoxycholic acid (UDCA), and deoxycholic acid (DCA) in the treatment for 2 or 8 weeks. The increase was, to a certain extent, also reflected in the group fed EEN-3PUFAs. By comparing results in groups fed with EEN-3PUFAs with the control group, the concentrations of CA, chenodeoxycholic acid (CDCA), UDCA, DCA, and lithocholic acid (LCA) in feces were also higher (p < 0.05) in the treatment for 2 or 8 weeks in mice, while their liver concentration was lower (p < 0.05). The concentration of taurine-conjugated BAs in the liver was similar in response to EEN-3PUFAs exposure in the treatment for 2 or 8 weeks, while glycine-conjugated BAs were affected to a greater extent. Thus, the group fed EEN-3PUFAs generally had higher feces concentrations of taurine-conjugated BAs than the control group. A similar increase of glycine-conjugated BAs in liver concentrations was seen in the group fed with EEN-3PUFAs compared with the control group. These trends were reflected a certain extent also in the groups fed EEN-3PUFAs, although with less significance. It should be emphasized that the BA pool in mice consisted mostly of hydrophilic bile acids, muricholic acids, and cholic acid, which was different from the hydrophobic BA pool composing of predominantly CDCA, CA, and DCA in humans. Actually, compared with the control group, the liver or feces concentration of taurine- or glycine-conjugated or free BAs generally increased in groups fed with EEN-3PUFAs, regardless of the treatment durations. The pathway for interactions of SCFAs, BAs, and the gut microbiome is briefly summarized in **Fig 8**.

Taken together, the results indicate that EEN-3PUFA modulate the gut microbiome by promoting the abundance of beneficial species and decreasing the abundance of pathogenic ones. EEN-3PUFAs also modulated the intestinal barrier and increased the production of acetic acid, propionic acid, and butyric acid, and shifted BAs towards unconjugated BAs. Those effects possibly explain the positive effects of EEN and 3PUFA in patients with IBD and CD [20,22–24,77,78]. Nevertheless, the exact mechanisms still need to be refined in future studies.

## Conclusions

In summary, the data suggested that EEN-3PUFAs improves gut microbiome composition by promoting the abundance of microbial beneficial species and decreasing the abundance of pathogenic ones, especially *Streptomyces*, *Proteobacteria*, *Putamen*, *Barnesiella*, and *Desulfovibrio*. Interestingly, the *Helicobacter* Genus was also elevated by EEN-3PUFAs, but the specific species remain to be further investigated and their functions to be further uncovered. Furthermore, EEN-3PUFAs also enhanced the intestinal barrier by decreasing peripheral levels of LPS and D-lactic acid. In addition, EEN-3PUFAs increased the production of SCFAs involving acetic acid, propionic acid and butyric acid, and shifted BAs towards unconjugated Bas; thereby lipid or bile metabolism. Nevertheless, the underlying mechanism are required to be further elucidated. Our study provides valuable data to future studies on n-3 PUFAs effects on metabolism and microbiome. However, large, well-designed trials should be performed to compare conventional and n-3 PUFAs-based formulas in humans.

## Supporting information

S1 Checklist (PDF)

S1 File (PDF)

S2 File (PDF)

S3 File (PDF)
