# Supplementary material for: Enteral nutrition modulation with n-3 PUFAs directs microbiome and lipid metabolism in mice
Source: PLoS One. 2021 Mar 25;16(3):e0248482. doi: 10.1371/journal.pone.0248482 (PMC7993877; doi:10.1371/journal.pone.0248482)
Supplement: S1 File — (PDF) [file pone.0248482.s002.pdf]

生工生物
宏基因组分类
测序项目报告

**合同编号**	16S190078
**客户单位**	浙江工业大学
**报告时间**	2019-02-20

目录

- **一. 术语解释**
- **二. 本项目使用软件与数据库**
  - 2.1 软件
  - 2.2 数据库
- **三. 项目分析流程**
  - 3.1 分析流程图
  - 3.2 详细分析内容列表
  - 3.3 分析步骤及方法简介
- **四. 分析结果展示**
  - 4.1 数据预处理
  - 4.2 去除嵌合体及非特异性扩增序列
  - 4.3 操作分类单元 (OTU) 分类
  - 4.4 OTU分布韦恩图
  - 4.5 OTU数目与聚类similarity值关系图
  - 4.6 基于OTU丰度的样本聚类图
  - 4.7 多样性指数分析
  - 4.8 稀疏性曲线
  - 4.9 Rank-abundance曲线
  - 4.10 物种分类学
  - 4.11 群落结构组分图
  - 4.12 样本与物种关系图
  - 4.13 单样品多级物种组成图
  - 4.14 分类和系统发育信息可视化
  - 4.15 物种丰度热图
  - 4.16 物种丰度3D柱状图
  - 4.17 物种分类箱线图
  - 4.18 基于物种丰度样本聚类树图
  - 4.19 样品聚类树与柱状图组合分析
  - 4.20 群落分类学系统组成树
  - 4.21 PCA分析
  - 4.22 NMDS非度量多维尺度分析
  - 4.23 Network图
  - 4.24 微生物间相互关系分析图
  - 4.25 系统发生进化树
  - 4.26 UniFrac分析
  - 4.27 基于UniFrac的多样品相似度树分析
  - 4.28 基于UniFrac的heatmap图
  - 4.29 基于UniFrac的PCoA分析
  - 4.30 UniFrac距离箱式图
  - 4.31 样本间菌群丰度差异分析
  - 4.32 Ternary Plot图
  - 4.33 Adonis/PERMANOVA分析
  - 4.34 PICRUSt功能分析
  - 4.35 功能组分图
  - 4.36 基于功能丰度的样本聚类图
  - 4.37 功能丰度热图
  - 4.38 样品聚类树与功能柱状图组合分析
  - 4.39 基于功能的PCA分析

4.40 基于功能的NMDS分析

- 4.41 Procrustes分析
- 4.42 功能累计曲线图
- 4.43 功能丰度差异分析

- **五. 分析结果文件说明**
- **六. 参考文献**

一. 术语解释

Bp: base-pair，碱基对，读长的单位，每一个bp指一对互补的碱基。

Read: 读长，测序数据中每一条序列就是一个read。

Raw_reads: 原始数据。

Clean_reads: QC之后的数据。

Barcode: 标签序列，位于reads的开头，用于区分这一条reads属于哪一个样本。分配完毕之后barcode会被删除。

Fastq: 序列数据存储的标准格式之一，每4行为一条read的信息。包含测序read名，序列，正反链标示，序列质量值。

Fasta: 序列数据存储的标准格式之一，每两行为一条read信息。包含测序reads名和序列。通常在QC之后，以此格式保存数据。

Pair-end测序: 双端测序，两端均测序，随后合并成一条read。

质量评分: 指的是一个碱基的错误概率的对数值，即质量评分越高，错误概率越小。

QC: Quality control，即质量控制。

低复杂度序列: 即有大量简单重复的序列。

嵌合体: PCR过程中，因为不同的模板混杂，错误产生的序列，这条序列并非真实存在。

靶区域外序列: 引物非特异性扩增产生的序列。

OTU: operational taxonomic unit (操作单元分类) 。要了解样品测序中群落分布信息，就需要对序列进行聚类（cluster），通过聚类，就可以根据序列的相似度分成很多序列的集合，每一个序列的集合就是一个OTU。

RDP: Ribosomal Database Project。为了得到每个OTU对应的物种分类信息，采用RDP classifier贝叶斯算法对97%相似度水平的OTU代表序列进行分类学分析，并在界门纲目科属水平，统计各个样品的菌落组成。

Node: 网络图概念，每一个点就是一个node，在本项目network中，node有三种形式:样本、OTU和物种分类。

Edge: 网络图概念，在network中，两点之间的连线就是edge。

Alpha多样性: 是指一个特定区域或生态系统内的多样性，经常用物种丰富度来度量。

Beta多样性: 不同生态系统之间多样性的比较，是物种组成沿环境梯度或者在群落间的变化率，用来表示生物种类对环境异质性的反应。

PCA分析: 在多元统计分析中，主成分分析PCA (Principal Component Analysis) 是一种简化数据集的技术。主成分分析经常用于减少数据集的维数，同时保持数据集中对方差贡献最大的特征，从而有效地找出数据中最“主要”的元素和结构，去除噪音和冗余，将原有的复杂数据降维，揭示隐藏在复杂数据背后的简单结构。

PCoA分析: PCoA分析 (Principal Co-ordinates Analysis) 是一种研究数据相似性和差异性的可视化方法。进过一系列的计算之后，选择主要的，排在前几位的特征值，对样本之间的关系进行描述。

RDA/CCA分析: 是基于对应分析发展而来的一种排序方法，将对应分析与多元回归分析相结合，每一步计算均与环境因子回归，又称多元直接梯度分析。

NMDS分析: 非度量多维尺度分析，是一种将多维空间的研究对象简化到低维空间进行定位，分析和归类，同时又保留对象间原始关系的数据分析方法。其特点是根据样品中包含的物种信息，以点的形式反映在多维空间上，而对不同样品间的差异程度，则是通过点与点的距离体现的，最终获得样品的空间定位点图。

滑窗法: 检测一个窗口内的碱基质量值，如果满足条件则向前移动一个单位继续检测，如果不满足条件即做删除处理，随后继续移动到下一个单位进行检测，直到检测完所有的数据。

RDP分类阈值: 即分类可信度，在RDP classifier中，使用bootstrapping方法估计分类的可信度。当可信度设置为≥80%时，V3、V4区的序列可以正确分配到属的概率分别是98.1%和95.7%，满足分析需要。[1]

**Variable region**	**V3**			**V6**			**V4**		
**Bootstrap cutoff (≥)**	0%	50%	80%	0%	50%	80%	0%	50%	80%
**Fraction of sequences classified to genus**	100%	92.4%	82.3%	100%	73.5%	40.4%	100%	97.0%	87.9%
Fraction of sequences correctly classified to genus	92.0%	95.0%	98.1%	79.0%	96.5%	98.7%	92.8%	94.5%	95.7%

Of 7,208 full-length 16S reference sequences from the human gut 6,054 were classified at genus-level with 80% bootstrap support. The RDP-classifier was trained with the latest training set No. 4 from December 2008. For each of the three extracted variable regions fragments were classified again, at three different bootstrap thresholds, and compared with the full-length classifications (last row).
doi:10.1371/journal.pone.0006669.t002

二. 本项目使用软件与数据库

2.1 软件

SoftWare Name	Version	R Package	Version
Prinseq[2]	0.20.4	vegan	2.0-10
FLASH[3]	1.2.3	ape	3.3
pear[4]	0.9.6	VennDiagram	1.6.16
Mothur[5]	1.30.1	scatterplot3d	0.3-36
Usearch[6]	5.2.236	pheatmap	1.0.7
Cytoscape	3.2	gplots	2.17.0
Qiime[7]	1.8.0	ggtern	2.1.1
R	3.2	igraph	1.0.1
Muscle[8]	3.8.31		
MEGAN[9]	5.7.1		
RDP classifier[10]	2.12		
NCBI Blast+[11]	2.28		
cutadapt	1.2.1		
Pynast[12]	1.2.2		
Uchime[13]	4.2.40		
Fasttree[14]	2.1.3		
STAMP[15]	2.1.3		
PICRUSt[16]	1.0.0		
GraPhlAn[17]	0.9.7		
LEfSe[18]	1.1.0		
Krona[19]	2.6.1		
iTOL[20]	3.2.1		
SparCC	1.0.0		

2.2 数据库

16S细菌古菌核糖体数据库:

RDP数据库：默认数据库，http://rdp.cme.msu.edu/misc/resources.jsp

Silva[21]数据库: http://www.arb-silva.de/

NCBI 16S数据库: http://ncbi.nlm.nih.gov/

18S真菌核糖体数据库:

Silva数据库：默认数据库，http://www.arb-silva.de/

NCBI 18S数据库：http://ncbi.nlm.nih.gov/

ITS真菌核糖体数据库:

RDP数据库：默认数据库，http://rdp.cme.msu.edu/misc/resources.jsp

Unite[22]数据库: http://unite.ut.ee/index.php

功能基因数据库:

FGR：RDP整理来源于GenBank的功能基因数据库，http://fungene.cme.msu.edu/

三. 项目分析流程

3.1 分析流程图

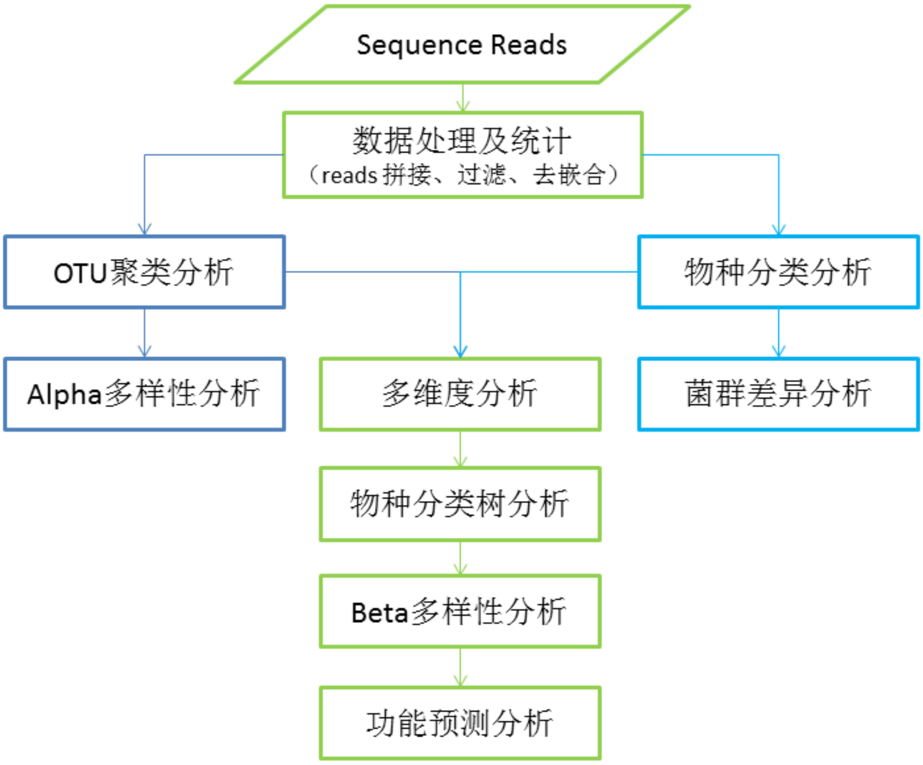

3.2 详细分析内容列表

分析项目	详细分析内容	说明
**1. 数据处理与统计**	1) 数据预处理	
	2) 去除嵌合体及非特异性扩增序列	
**2. OTU聚类分析**	1) 操作分类单元 (OTU) 聚类	韦恩图需要样本数或组数大于1小于6，聚类树需要样本数大于1
	2) OTU分布韦恩图	
	3) OTU数目与聚类similarity值关系图	
	4) 基于OTU丰度的样本聚类图	
**3. Alpha多样性分析**	1) 多样性指数分析	物种累计曲线需要样本数大于10

分析项目	详细分析内容	说明
	2) 稀疏性曲线	
	3) Rank-abundance曲线	
	4) Specaccum物种累积曲线	
**4. 物种分类分析**	1) 物种分类学<br>2) 群落结构组分图<br>3) 单样品多级物种组成图<br>4) 分类和系统发育信息可视化<br>5) 物种丰度热图<br>6) 物种丰度3D柱状图<br>7) 基于物种丰度样本聚类树图<br>8) 样品聚类树与柱状图组合分析<br>9) 群落分类学系统树状图	热图、聚类图需要样本数大于1
**5. 多维度分析**	1) PCA分析<br>2) NMDS非度量多维尺度分析<br>3) OTU、物种与环境因子相关性分析<br>4) RDA/CCA分析<br>5) Network图分析<br>6) 微生物间相互关系分析图	PCA/NMDS分析需要样本数大于4，环境因子相关分析需要提供环境因子，Network图分析需要样本数大于1，微生物间相互关系分析图需要样本数大于2
**6. 物种分类树分析**	1) 系统发生进化树	
**7. Beta多样性分析**	1) UniFrac分析<br>2) 基于UniFrac的多样品相似度树分析<br>3) 基于UniFrac的heatmap图<br>4) 基于UniFrac的PCoA分析<br>5) UniFrac距离箱式图	该分析需要样本数大于1，其中PCoA分析需要样本数大于4，箱型图需要分组信息
**8. 菌群差异分析**	1) 样本间菌群丰度差异分析<br>2) Ternary Plot图<br>3) LEfSe分析<br>4) Anosim分析<br>5) PERMANOVA分析	该分析需要样本数大于1，其中Ternary Plot分析需要样本数大于2，Anosim分析、ANOVA分析、PERMANOVA分析和LEfSe分析需要分组信息

分析项目	详细分析内容	说明
	6) ANOVA分析	
**9. 功能预测分析**	1) PICRUSt功能分析	该分析仅适用于16S 项目，其中聚类热图差异等分析需要样本数大于2，PCA、NMDS、功能累计曲线分析需要样本数大于4
	2) 功能组分图	
	3) 基于功能丰度的样本聚类图	
	4) 功能丰度热图	
	5) 样品聚类树与功能柱状图组合分析	
	6) 基于功能的PCA分析	
	7) 基于功能的NMDS分析	
	8) 功能累计曲线图	
	9) 功能丰度差异分析	

3.3 分析步骤及方法简介

1. **数据处理**
   1) 通过barcode区分样品序列，并对各样本序列做QC。
   2) 去除非特异性扩增序列及嵌合体。
4. **OTU聚类分析: 将多条序列根据其序列之间的距离来对它们进行聚类，后根据序列之间的相似性作为域值分成操作分类单元 (OTU)**
   1) 在OTU聚类结果的基础上，获取每一个OTU聚类中的代表性序列。**默认的，我们选择丰度最高的序列作为代表性序列**
   2) 根据各样本在OTU的分布情况绘制韦恩图和聚类树图
7. **Alpha多样性分析: 衡量样本物种多样性**
   1) 计算ACE/Chao/Shannon/Simpson/Coverage等物种多样性指数，并制作所有样品ACE/Chao/Shannon/Simpson/Richness指数的箱形图和稀释性曲线。
   2) 根据各样本的OTU丰度分布情况绘制Rank-abundance曲线。
10. **物种分类分析: 将序列进行物种分类，对每个样本和每个物种单元分类进行序列丰度计算构建样本和物种分类单元序列丰度矩阵**
   1) 物种丰度图。基于物种分类分析，绘制物种分类条形图，物种丰度饼图，物种丰度热图，classifier分类图，单样本群落分布丰度柱状图，群落分布分度3D图，样本聚类与柱状图组合分析图。
   2) 系统发育信息图，绘制反映在每一个层级上各样本群落分布丰度。
13. **多维度分析: 基于OTU聚类分析和物种分类的共有分析**
   1)

PCA/NMDS分析。根据物种分类单元和样本丰度矩阵，利用降维的方法来反应样本之间的距离。

2) 网络图分析。根据样本OTU丰度和物种分类丰度，得到样本与OTU、物种分类之间的关系，绘制网络图。同时根据得到的OTU之间的相关性和物种分类之间的相关性做相互关系分析图。
3) 物种，OTU/环境因子相关性分析。计算物种或者OTU与环境因子的相关性，判断其显著性，最终获得显著影响某些物种的环境因子 (需提供环境因子信息，且环境因子的数目最好小于样本数目)。
4) RDA/CCA分析。检测环境因子、样品、群落分布三者之间的关系或者两两之间的关系 (需提供环境因子信息，且环境因子的数目最好小于样本数目)。

18. 物种分类树分析: 绘制所有群落分布之间的进化树图，同时绘制OTU聚类结果中丰度较高的OTU的进化树图，并标注其所属的群落分布信息。

19. Beta多样性分析: 比较多(组)样本之间的差别度量

1) 根据多序列队列构建代表性序列为节点的进化树，利用Unifrac算法计算样本距离。
2) 根据样本之间的距离绘制样本聚类树、样本热图、样本PCoA、组内距离箱型图。

22. 菌群差异分析

1) 根据物种分类单元和样本丰度矩阵，利用统计检验筛选样本组间的差异物种分类单元，绘制误差线图、LEfSe图、Ternary Plot图。
2) 相似性分析(Anosim)，用来检验组间（两组或多组）的差异是否显著大于组内差异，从而判断分组是否有意义。
3) 方差分析(ANOVA)，用于两个及两个以上样本均数差别的显著性检验。

26. 功能预测分析

1) 根据对已有测序微生物基因组的基因功能的构成分析结果和测序获得的物种构成，推测样本中的功能分类的构成。
2) 根据功能分类丰度，绘制功能分类条形图、丰度热图、丰度柱状图、丰度聚类树图，并进行PCA/NMDS分析。
3) 根据功能分类丰度在样本之间的差异，利用统计检验筛选样本组间的差异功能分类单元。

四. 分析结果展示

4.1 数据预处理

4.1.1 原始序列数据

Illumina Miseq™得到的原始图像数据文件经CASAVA碱基识别 (Base Calling) 分析转化为原始测序序列 (Sequenced Reads) ，我们称之为 Raw Data或Raw Reads，结果以 FASTQ (简称为fq)文件格式存储，其中包含测序序列 (reads) 的序列信息以及其对应的测序质量信息。

FASTQ格式文件中每个read由四行描述，如下所示:

```
@MISEQ03:113:000000000-AFJGE:1:1101:12409:1286 1:N:0:TCTACA
NAAGAACACGTTCGGTCACCTCAGCACACTTGTGAATGTCATGGGATCCAT
+
#55???BBBBB?BA@DEEFFCFFHHFFCFFHHHHHHHFAE0ECFFD/AEHH
```

其中第一行以“@”开头，随后为Illumina 测序标识符 (Sequence Identifiers) 和描述文字 (选择性部分) ；

第二行是碱基序列；

第三行以“+”开头，随后为Illumina 测序标识符 (选择性部分) ；

第四行是对应碱基的测序质量，该行中每个字符对应的 ASCII 值减去 33，即为对应第二行碱基的测序质量值。

Illumina 测序标识符详细信息详见下表：

MISEQ03	Instrument - unique identifier of the sequencer
113	run number - Run number on instrument
000000000-AFJGE	FlowCell ID - ID of flowcell
1	LaneNumber - positive integer
1101	TileNumber - positive integer
12409	X - x coordinate of the spot. Integer which can be negative
1286	Y - y coordinate of the spot. Integer which can be negative
1	ReadNumber - 1 for single reads; 1 or 2 for paired ends
N	whether it is filtered - NB: Y if the read is filtered out, not in the delivered fastq file, N otherwise

0	control number - 0 when none of the control bits are on, otherwise it is an even number
TCTACA	Illumina index sequences

Miseq™的碱基测序质量值 (Phred quality score,Q_{phred}) 是测序错误率 (base-calling error probabilities,P) 的整数映射，映射关系为：Q_{phred}= -10log_{10}(P)。

Phred quality scores are logarithmically linked to error probabilities		
**Phred Quality Score**	**Probability of Incorrect Base Call**	**Base Call Accuracy**
10	1 in 10	90%
20	1 in 100	99%
30	1 in 1,000	99.9%
40	1 in 10,000	99.99%

4.1.2 数据预处理

Miseq测序序列中含有barcode序列，以及测序时加入的引物和接头序列。首先需要去除引物接头序列，再根据PE reads之间的overlap关系，将成对的reads 拼接 (merge) 成一条序列，然后按照barcode标签序列识别并区分样品得到各样本数据，最后对各样本数据的质量进行质控过滤，得到各样本有效数据。

数据优化方法和参数：

1) 去除3'端测序引物接头，Read1 3' 端测序接头为TGGAATTCTCGGGTGCCAAGGAACTC。
2) 根据PE reads之间的overlap关系将成对reads拼接 (merge) 成一条序列。
3) 根据各样本barcode序列从融合后数据中分割出各样本数据。
4) 去除各样本中reads尾部质量值在20以下的碱基。设置10bp的端口，如果窗口内的平均质量值低于20，从窗口开始去除后端的碱基。
5) 切除reads中含N部分序列，并去除数据中的短序列，长度阈值200bp。
6) 随后再对低复杂度的序列进行过滤

软件: **cutadapt** (去除接头，主要参数: -O 5 -m 50)，**PEAR**(序列对拼)，**Prinseq** (质量剪切，主要参数：-lc_method dust -lc_threshold 40 -min_len 200)。

4.1.3 结果说明

结果目录: **1_data_for_analysis/**

1_Raw_data: 测序原始序列数据。

2_Merged_data: PE reads对拼后的序列。

3_QC_data/ALL_raw_read_len_distribution.pdf: QC之前原始序列长度分布图。

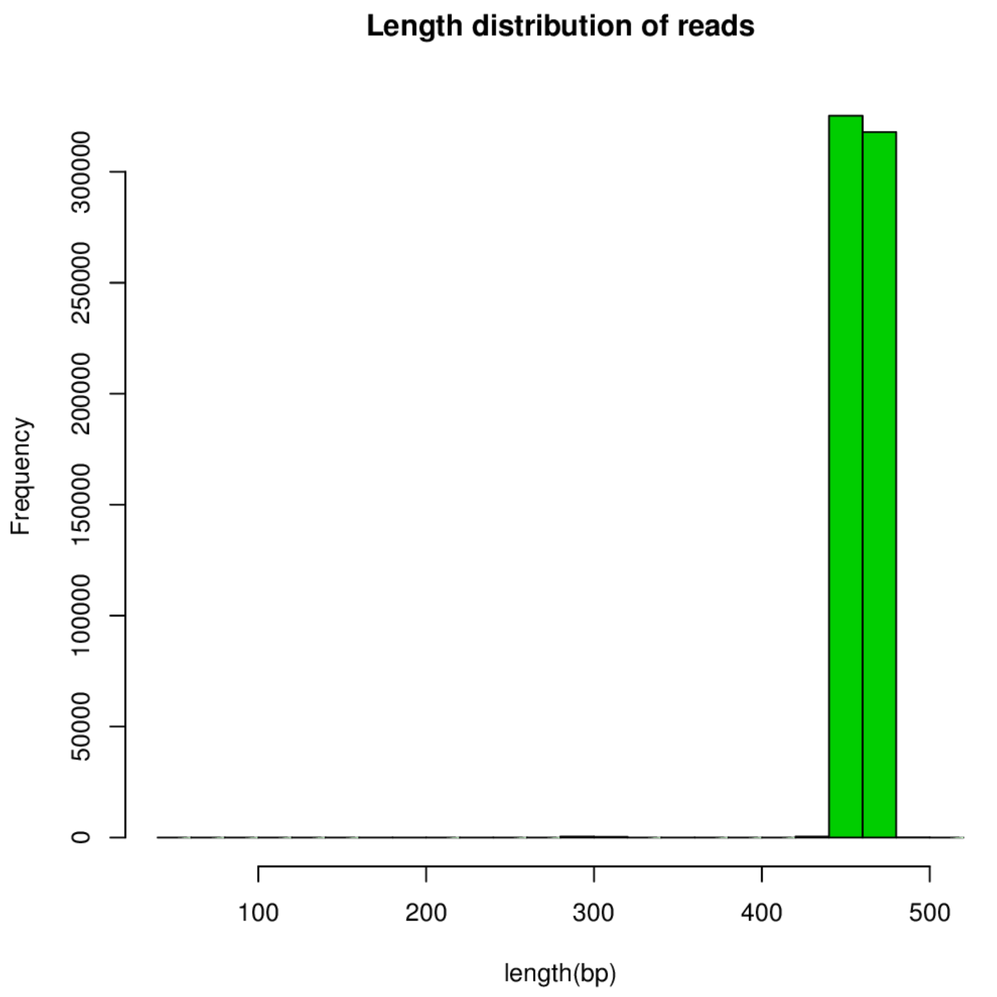

图1.1 原始数据长度分布图
原始序列使用pear融合后的序列长度，以及该长度reads数目。

3_QC_data/ALL_QC_read_len_distribution.pdf: QC之后序列长度分布图。

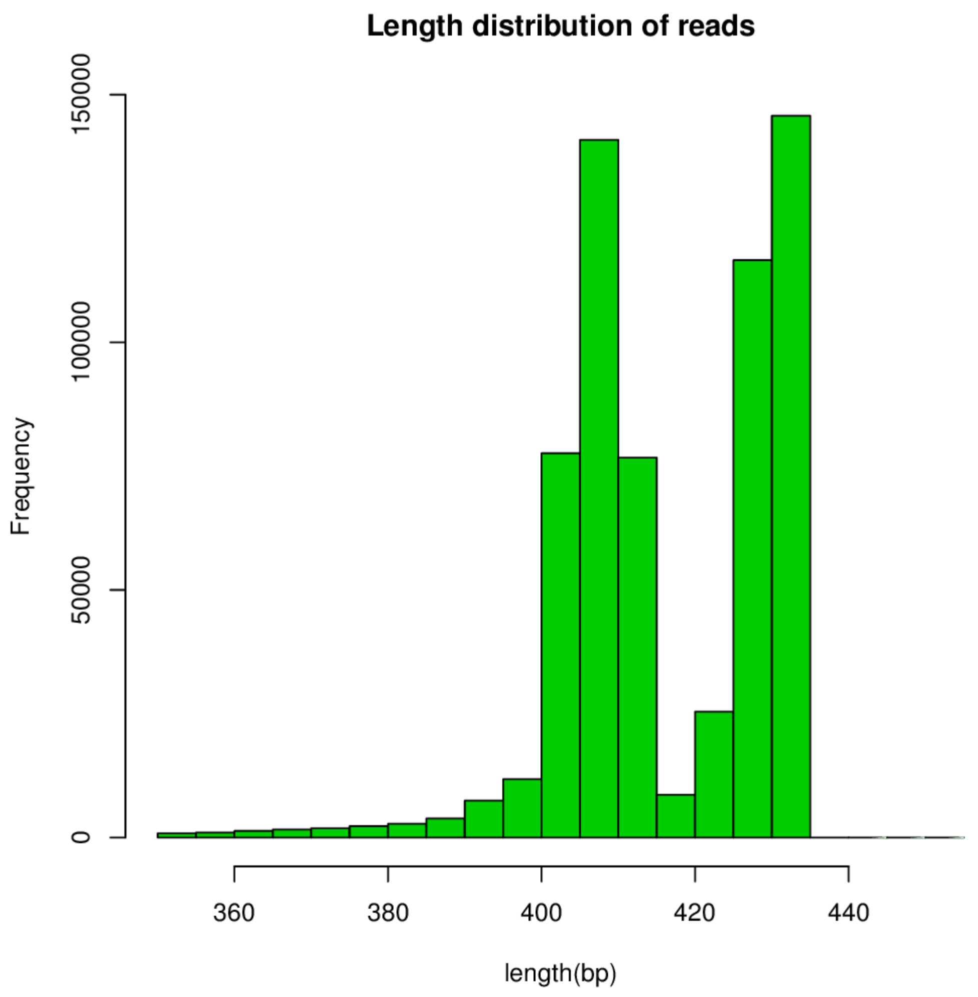

图1.2 质控后序列长度分布图
质控(去除barcode、primer以及部分低质量序列)后序列长度，以及该长度reads数目。

Sample_infor.xls: QC之后序列统计总表。

表1.1 各样本数据信息统计

Group	Sample	Barcode	Raw num	Mean len	Clean num	Mean len
16S190078	1	TGTTAT	60452	462.25	58646	422.13
16S190078	2	GCCATC	82689	450.29	80440	410.38
16S190078	3	TGTGTT	67398	455.47	65440	415.86
16S190078	4	TAGGAC	76438	449.88	74315	409.68
16S190078	5	TGGACG	41517	456.71	40229	416.66
16S190078	6	CGATGT	64191	455.90	62294	415.89

Group	Sample	Barcode	Raw num	Mean len	Clean num	Mean len
16S190078	7	TCCTGT	65856	446.37	63911	406.52
16S190078	8	GAAGGC	67685	465.34	66083	425.23
16S190078	9	ATGTCA	53397	468.52	52111	428.11
16S190078	10	CGGTTA	64894	466.49	63023	426.39

Group: 样本的分组信息
Sample: 样本名
Barcode: 区分样本使用的barcode信息
Raw_num: 原始reads数目
Mean_len: 原始序列的平均长度
Clean_num: QC之后剩余reads数目
Mean_len: QC之后序列平均长度

4.2 去除嵌合体及非特异性扩增序列

4.2.1 分析方法

在PCR反应过程中,会由于延伸不完全产生一些不完整的扩增产物,这些不完整的扩增产物在下一轮的扩增循环中,与序列相近的其他同源模板退火,继续延伸另一种模板的序列而得到的一种杂合的DNA片段，这种片段称之为Chimera (嵌合体)。同时也会产生一些非特异性扩增序列。为了保证信息分析质量，必须对其进行剔除。

使用**Usearch**去除预处理后序列中非扩增区域序列，而后对序列进行测序错误校正，并调用**uchime**进行鉴定嵌合体。随后，我们再将去除嵌合体的序列与数据库代表性序列进行**blastn**比对，低于阈值的比对结果我们认为是靶区域外序列，并剔除掉该部分序列。

4.2.2 结果说明

结果目录：**2_filter_chimeras/**

filter_chimeras_result.xls:去除嵌合体与靶区域外序列统计表。

表2.1 处理后结果统计表

Group	Sample	Seq num	Organelle num	Out target num	Chimeras num	Filtered num
16S190078	1	58646	22	0	225	58399
16S190078	2	80440	29	0	187	80224

Group	Sample	Seq num	Organelle num	Out target num	Chimeras num	Filtered num
16S190078	3	65440	131	0	89	65220
16S190078	4	74315	4	0	187	74124
16S190078	5	40229	3	1	907	39318
16S190078	6	62294	3	0	99	62192
16S190078	7	63911	10	0	4336	59565
16S190078	8	66083	43	0	206	65834
16S190078	9	52111	2	0	240	51869
16S190078	10	63023	6	0	232	62785

Group: 样本的分组信息
Sample: 样本名
Seq_num: 处理之前序列总数
Organelle_num: 比对到细胞器组织序列数目
Out_target: 非靶区域序列数目
Chimeras_num: 嵌合体数目
Filtered_num: 处理后剩余序列

4.3 操作分类单元 (OTU) 分类

4.3.1 分析方法

OTU (Operational Taxonomic Units) 指的是在系统发生学或群体遗传学研究当中，为了便于分析，人为的给每个分类单元设置的同一标志。要了解一个样品测序结果中的菌种、菌属等信息，就需要对序列进行归类操作。方法为将所有样本序列按照序列间的距离进行聚类，后根据序列之间的相似性将序列分成不同的操作分类单元 (OTU)。通常在97%的相似水平下的OTU进行生物信息统计分析。

在OTU聚类结果的基础上，获取OTU聚类中的代表性序列， 默认的，我们选择丰度最高的序列作为OTU的代表性序列，进行各类的OTU分析。同时在大于5个样本的时候，会去除只对应一条reads的OTU。

软件: **Usearch**。

4.4 OTU分布韦恩图

4.4.1 分析方法

VENN图可以用来统计样本中共有的和独有的OTU的数目，直观的展现出环境样品的OTU数目组成相似性及重叠情况。

使用软件: **R**的**VennDiagram** package。

4.4.2 结果说明

结果目录: **3_OTU/VENN/**

***_venn.pdf:** 样本间或者组间OTU韦恩图

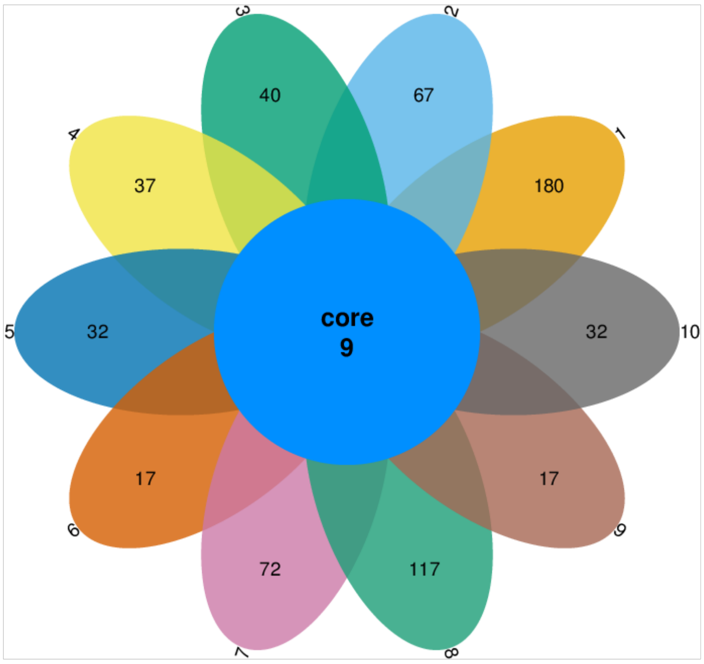

图4.1 OTU 样本分布韦恩图或花瓣图
不同样品 (组) 用不同颜色表示，图中数字代表特异或共有的OTU数。韦恩图构建所需样本数一般为2-5个，多于5个时，用花瓣图展示。

4.5 OTU数目与聚类similarity值关系图

4.5.1 分析方法

通过绘制OTU数目变化与聚类similarity值之间的关系图，从中选择最佳的similarity值进行OTU分析和分类学分析。

软件: **Usearch**。

4.5.2 结果说明

结果目录: **3_OTU/**

gradient_plot.pdf: OTU数目与聚类similarity值关系图

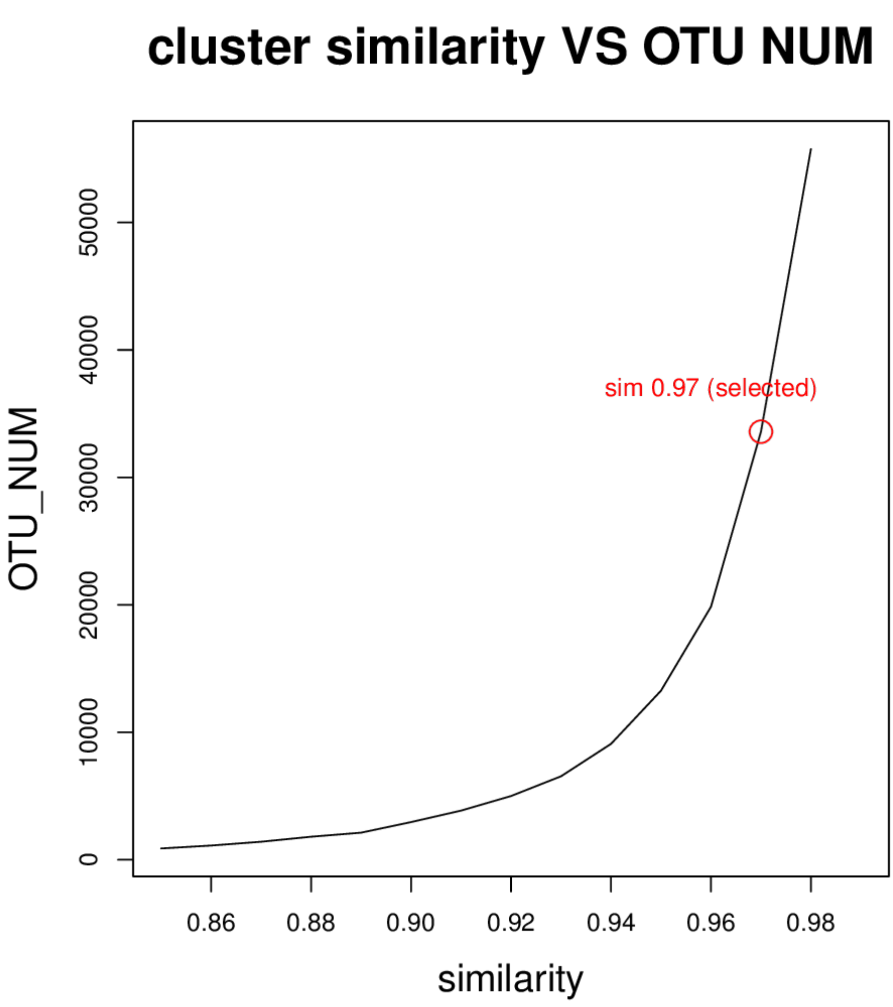

图5.1 OTU数目与聚类similarity值关系图
图中展示了不同similarity值 (0.86–0.99) 与OTU数目之间的关系。红字标示的是本次分析使用的similarity值。

4.6 基于OTU丰度的样本聚类图

4.6.1 分析方法

样本聚类树图可以通过树枝结构直观的反应出多个样品间的相似性和差异关系。首先根据beta多样性距离矩阵进行层次聚类 (Hierarchical cluatering) 分析，再使用非加权组平均法UPGMA (Unweighted pair group method with arithmetic mean) 算法构建树状结构，得到树状关系形式用于可视化分析

软件: 使用**R**的**vegan** package根据各样本OTU丰度计算beta多样性距离矩阵，计算样本间距离的方法为Bray-Curtis。

4.6.2 结果说明

结果目录: **3_OTU/bray_crutis_tree/**

OTU_NMDS_bray_crutis_tree.phylo: 样本的聚类树作图数据

OTU_NMDS_bray_crutis_tree.pdf: 所有样本的聚类树图

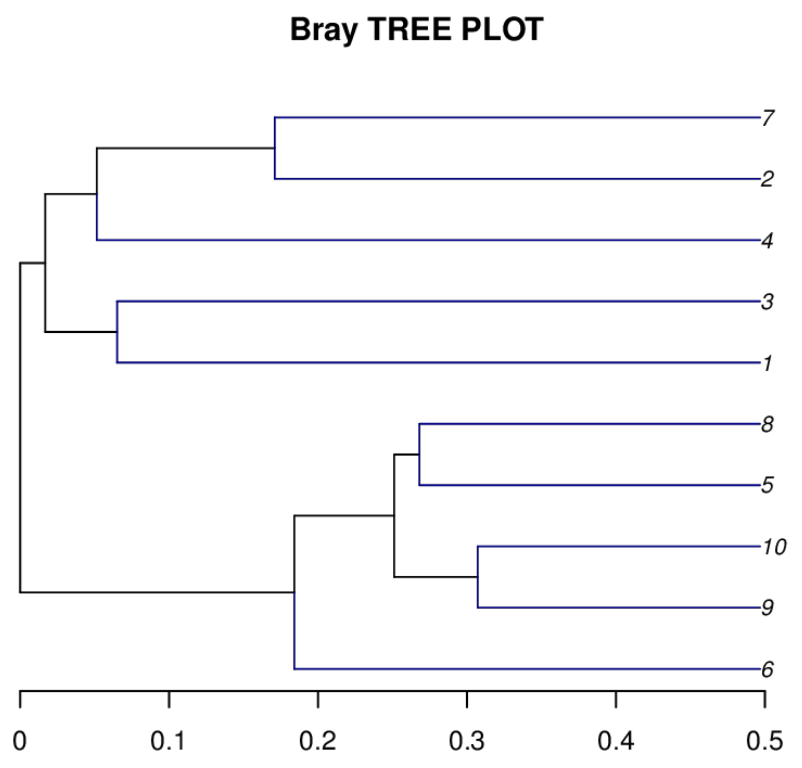

图6.1 基于OTU的样本聚类树图
树枝的长度代表样本间的距离，越相似的样本会越靠近，图中同一颜色的树枝表示来源于同一组。

4.7 多样性指数分析

4.7.1 分析方法

群落生态学中研究微生物多样性，通过单样品的多样性分析 (Alpha多样性) 可以反映微生物群落的丰度和多样性，包括一系列统计学分析指数估计环境群落的物种丰度和多样性。

计算群落分布丰度 (Community richness) 的指数有:

Sobs - the observed richness (http://www.mothur.org/wiki/Sobs)

Chao - the Chao1 estimator (http://www.mothur.org/wiki/Chao)

ACE - the ACE estimator (http://www.mothur.org/wiki/Ace)

计算群落分布多样性 (Community diversity) 的指数有：:

Shannon - the Shannon index (http://www.mothur.org/wiki/Shannon)

Simpson - the Simpson index (http://www.mothur.org/wiki/Simpson)

Coverage - the Good's coverage (http://www.mothur.org/wiki/Coverage)

各指数算法如下:

Chao: 用chao1算法估计群落中含OTU数目的指数，chao1在生态学中常用来估计物种总数，由Chao (1984) 最早提出。计算公式如下：

$$S_{chao1} = S_{obs} + \frac{n_1(n_1-1)}{2(n_2+1)}$$

其中，

S_{chao1} = 估计的OTU数

S_{obs} = 实际观测到的OTU数

n_1 = 只含有一条序列的OTU数目 (如 "singletons")

n_2 = 只含有两条序列的OTU数目 (如 "doubletons")

Ace: 用来估计群落中OTU数目的指数，由Chao提出，是生态学中估计物种总数的常用指数之一，与Chao 1的算法不同。计算公式如下：

$$S_{ACE} = \begin{cases} S_{abund} + \frac{S_{rare}}{C_{ACE}} + \frac{n_1}{C_{ACE}}\hat{\gamma}^2_{ACE}, & \text{for } \hat{\gamma}_{ACE} < 0.80 \\ S_{abund} + \frac{S_{rare}}{C_{ACE}} + \frac{n_1}{C_{ACE}}\tilde{\gamma}^2_{ACE}, & \text{for } \hat{\gamma}_{ACE} \geqslant 0.80 \end{cases}$$

其中，

$$N_{rare}=\sum_{i=1}^{abund} in_i \quad C_{ACE}=1-\frac{n_1}{N_{rare}}$$

$$\hat{\gamma}_{ACE}^2=max\left[\frac{S_{rare}}{C_{ACE}}\frac{\sum_{i=1}^{abund}i(i-1)n_i}{N_{rare}(N_{rare}-1)}-1,0\right]$$

$$\tilde{\gamma}_{ACE}^2=max\left[\hat{\gamma}_{ACE}^2\left\{1+\frac{N_{rare}(1-C_{ACE})\sum_{i=1}^{abund}i(i-1)n_i}{N_{rare}(N_{rare}-C_{ACE})}\right\},0\right]$$

n_i = 含有i条序列的OTU数目

S_{rare} = 含有"abund"条序列或者少于"abund"的OTU数目

S_{abund} = 多于"abund"条序列的OTU数目

abund = “优势”OTU的阈值，默认为10

Shannon: 用来估算样品中微生物多样性指数之一。它与Simpson多样性指数常用于反映alpha多样性指数。**Shannon值越大，说明群落多样性越高。**计算公式如下：

$$H_{shannon}=-\sum_{i=1}^{S_{obs}}\frac{n_i}{N}ln\frac{n_i}{N}$$

其中，

S_{obs} = 实际观测到的OTU数

n_i = 第i个OTU包含的序列数

N = 所有个体数目，此处为序列总数

Simpson: 用来估算样品中微生物多样性指数之一，由Edward Hugh Simpson (1949) 提出，在生态学中常用来定量描述一个区域的生物多样性。**Simpson指数值越大，说明群落多样性越低。**计算公式如下：

$$D_{simpson}=\frac{\sum_{i=1}^{S_{obs}}n_i(n_i-1)}{N(N-1)}$$

其中，

S_{obs} = 实际观测到的OTU数

n_i = 第i个OTU包含的序列数

N = 所有个体数目，此处为序列总数

Coverage: 各样品文库的覆盖率，其数值越高，则样本中序列没有被测出的概率越低。该指数实际反映了本次测序结果是否代表样本的真实情况。计算公式如下：

$$C = 1 - \frac{n_1}{N}$$

其中，

n_1 = 只含有一条序列的OTU数目 (如 "singletons")

N = 所有个体数目，此处为序列总数

软件: **mothur**。

4.7.2 结果说明

结果目录: **4_alpha_index/diversity_box/**

alpha_diversity_change_name.xls: alpha多样性指数统计表

表7.1 处理后结果统计表

Sample ID	Seq num	OTU num	Shannon index	ACE index	Chao1 index	Coverage	Simpson
1	54694	363	2.59	408.43	386	1.00	0.15
10	59331	163	1.37	205.36	190.5	1.00	0.43
2	76264	340	2.63	492.36	439.82	1.00	0.12
3	62223	301	2.78	374.13	355.81	1.00	0.13
4	70564	210	1.19	302.09	255.78	1.00	0.59
5	37315	211	1.77	418.50	322.56	1.00	0.33
6	59162	229	2.66	373.39	308.29	1.00	0.16
7	56449	333	3.40	526.91	440.61	1.00	0.06
8	62243	350	2.13	439.28	407.48	1.00	0.30
9	48942	144	0.43	274.06	197.2	1.00	0.87

Sample_ID: 样本名称
Seq_num: 样本的优质reads数目
OTU_num: 样本聚类得到的OTU数目
其余5列分别是5种Alpha指数的数值

Alpha_diversity_*.pdf: 组间Alpha指数箱式图

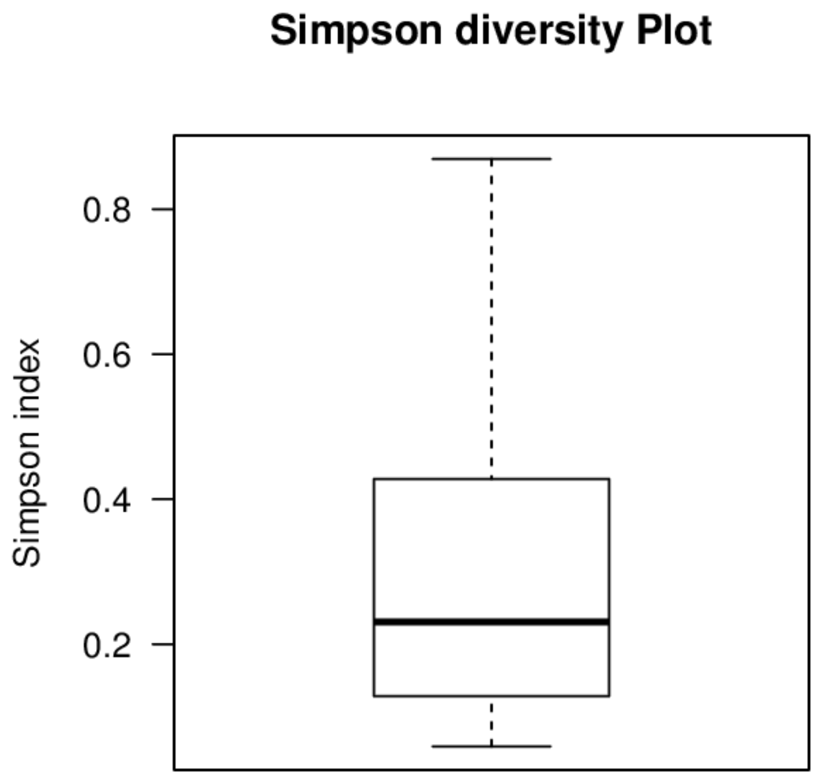

图7.1 分组间Alpha指数箱式图
图中每一个盒代表一个分组下面所有样本的Alpha指数，通过该图可以直观的看出每组样本下指的离散及分布情况，盒子越窄说明该组内样本离散程度越小，在一定程度下可反应出该组内样本的保守性。(以Simpson指数为例)

4.8 稀疏性曲线

4.8.1 分析方法

采用对测序序列进行随机抽样的方法，以抽到的序列数与它们所能代表OTU的数目构建曲线，即稀释曲线(Rarefaction Curve)。它可以用来比较测序数据量不同的样本中物种的丰富度，也可以用来说明样本的测序数据量是否合理。当曲线趋向平坦时，说明测序数据量合理，更多的数据量只会产生少量新的OTU，反之则表明继续测序还可能产生较多新的OTU。

软件: 使用97%相似度的OTU，利用**mothur**做rarefaction分析，利用**R**制作曲线图。

4.8.2 结果说明

结果目录: **4_alpha_index/Rarefaction/**

***_rarefaction_plot.pdf:** Alpha指数稀疏曲线图

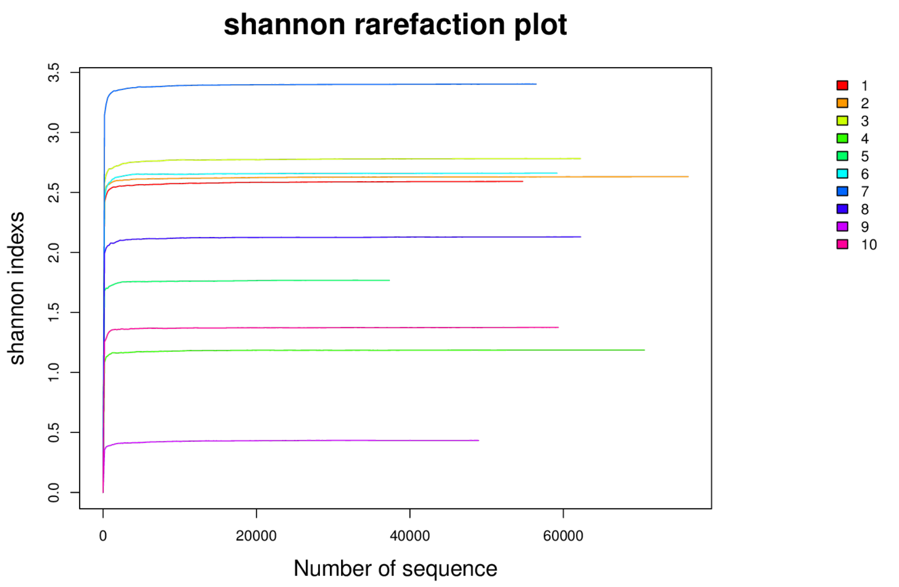

图8.1 Alpha指数稀疏曲线图
横坐标为样本中随机抽取序列数，纵坐标为所得相应的Alpha指数，每条曲线是一个样本。(以Shannon指数为例)

r_*_rarefaction_result.xls: Alpha指数稀疏曲线分析统计表

4.9 Rank-abundance曲线

4.9.1 分析方法

Rank-abundance曲线是分析多样性的一种方式。构建方法是统计单一样品中，每一个OTU所含的序列数，将OTUs按丰度（所含有的序列条数）由大到小等级排序，再以OTU等级为横坐标，以每个OTU中所含的序列数（也可用OTU中序列数的相对百分含量）为纵坐标做图。

Rank-abundance曲线用于同时解释样品多样性的两个方面，即样品所含物种的丰富程度和均匀程度。物种的丰富程度由曲线在横轴上的长度来反映，曲线越宽，表示物种的组成越丰富；物种组成的均匀程度由曲线的形状来反映，曲线越平坦，表示物种组成的均匀程度越高。

软件：利用**R**制作曲线图。

4.9.2 结果说明

结果目录: **4_alpha_index/Rank_Abundance/**

rank_abundance.pdf: Rank_Abundance曲线图

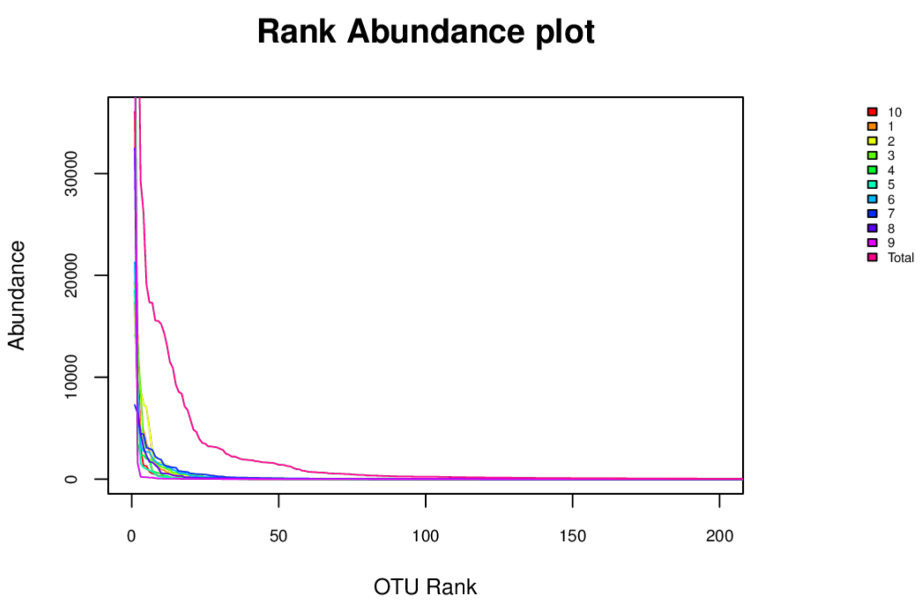

图9.1 Rank_Abundance曲线图
横坐标为按OTU相对丰度含量等级降序排列的OTU Rank值，纵坐标为样本在这个OTU下的丰度数值，每条曲线是一个样本。

4.10 物种分类学

4.10.1 分析方法

为了得到每个OTU对应的物种分类信息,需要对OTU进行物种分类，物种分类有两种方式:

RDP classifier: RDP classifier基于Bergey's taxonomy，采用Naïve Bayesian assignment算法对每条序列在不同层级水平上计算其分配到此rank中的概率值。一般认为概率值 (即RDP分类阈值) 大于0.8时此分类结果可信 (测序片段长度<250时可适当调低此值到0.5，如只测V3、V6、V4区)。Bergey's taxonomy分为6层，它们依次为域 (domain)、门 (phylum)、纲 (class)、目 (order) 、科 (family)、属 (genus)。默认采用该方法来进行物种分类。

Blast: 利用blastn将OTU序列与对应数据库进行比对，筛选出OTU序列的最佳比对结果，并对比对结果进行过滤，默认满足相似度>90%且coverage>90%的序列被用来后续分类，不满足条件的序列则被归为unclassified。

物种分类采用的数据库详见2.2 数据库。

根据分类学分析结果，统计在各个分类层级水平上的每个样品的群落组成。

4.10.2 结果说明

结果目录: **5_Taxonomic_Classification/plot_raw_file/**

***_reads_change_name.txt:** 各样本主要taxonomy对应reads数目

表10.1 genus水平上各样本主要rank reads数目

	1	2	3	4
Escherichia/Shigella	3	7360	43	613
Akkermansia	2	3	1	54082
Blautia	159	20170	639	2020
Bifidobacterium	1943	8845	16961	2034
Lachnospiracea incertae sedis	4785	9042	2269	423
Gemmiger	1	1	428	2423
Clostridium XVIII	4815	5481	1205	121
Raoultella	17543	0	1	0
Dorea	3	12262	205	2414
Erysipelotrichaceae incertae sedis	257	349	274	1
Enterococcus	62	4	14155	39
Citrobacter	8510	4	10	78
Faecalibacterium	2	1610	8622	1
Collinsella	515	1	1201	3528

第一列表示taxonomy名字，后面每列为各样本在genus分类水平下的序列数目。

***_ratio_change_name.txt:** 各样本主要taxonomy对应reads数与总reads数的百分比，结果同上

4.11 群落结构组分图

4.11.1 分析方法

根据分类学分析结果，可以得知一个或多个样品在各分类水平上的分类学比对情况。在结果中，包含了两个信息:

(1).样品中含有何种微生物

(2).样品中各微生物的序列数，即各微生物的相对丰度

因此，可以使用统计学的分析方法，观测样品在不同分类水平上的群落结构。将多个样品的群落结构分析放在一起对比时，还可以观测其变化情况。根据研究对象是单个或多个样品，结果可能会以不同方式展示。通常使用较直观的饼图或柱状图等形式呈现。群落结构的分析可在任一分类水平进行。

软件：利用**R**对物种分类学统计结果进行作图。

4.11.2 结果说明

结果目录: **5_Taxonomic_Classification/**

barplot/*/*_barplot.pdf: 所有样本群落结构分布柱状图

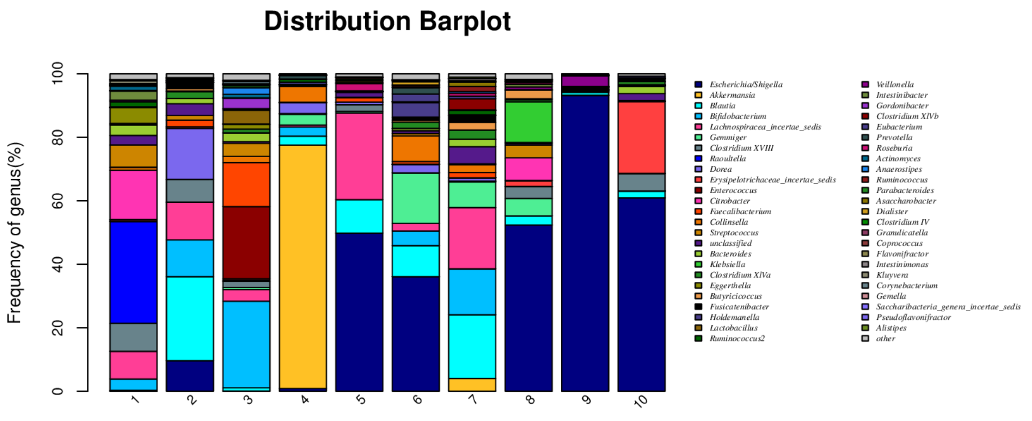

图11.1 genus水平所有样本群落结构分布图
横轴为各样品的编号，纵轴为相对丰度比例。颜色对应此分类学水平下各物种名称，不同色块宽度表示不同物种相对丰度比例。

pie_plot/*/*_*_2D_pie_plot.pdf: 单样本物种丰度饼图2D图

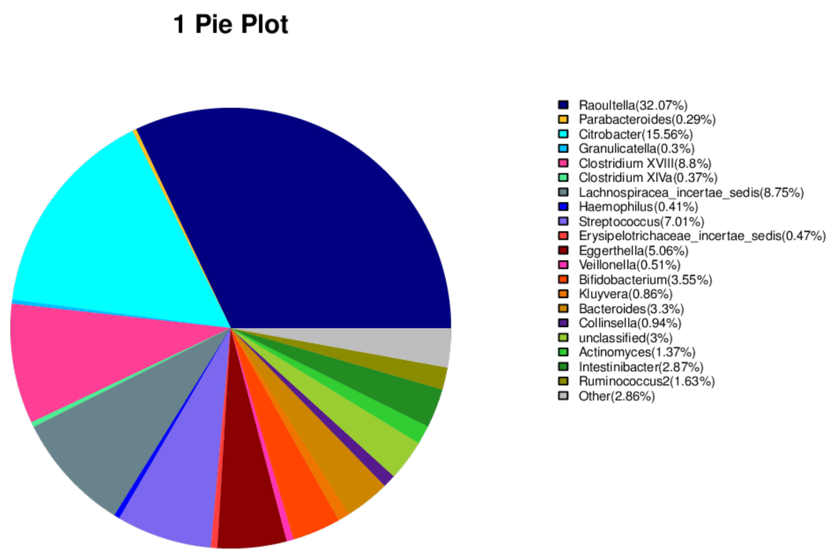

图11.2 genus水平单样本的丰度饼图2D图

pie_plot/*/*_*_3D_pie_plot.pdf: 单样本物种丰度饼图3D图

pie_plot/*/*_labeled_*_2D_pie_plot.pdf: 单样本物种丰度饼图2D图，饼图外圈标注信息

sample_barplot/*/*_*_sample_barplot.pdf: 单样本物种丰度柱状图

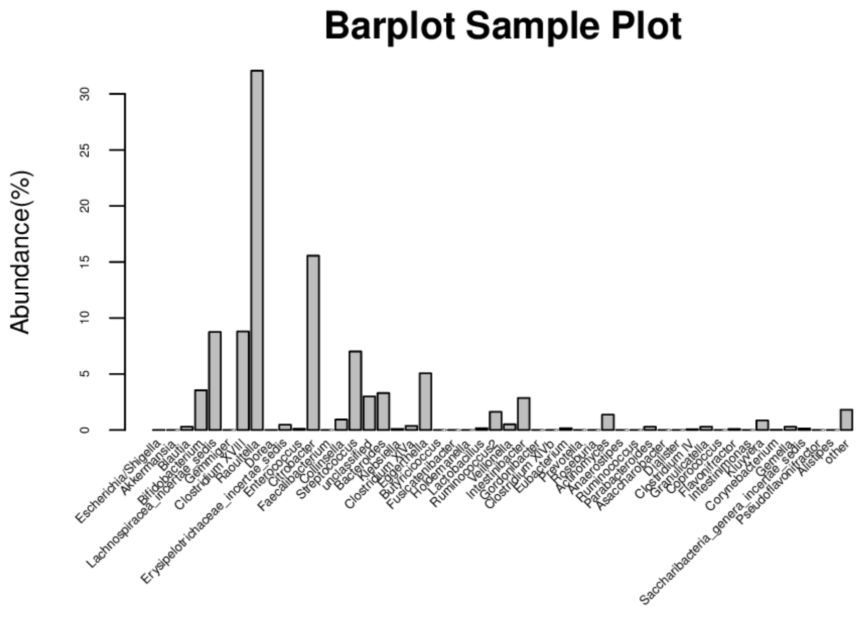

图11.3 genus水平物种分类丰度单样本柱状图

genus水平下，样本物种分类中丰度占比最高的前50个物种分类的分布情况，并按照总体丰度从大到小排序。总体丰度指的是，在所有样本中物种分类reads num的总值。为了展示效果，只显示前50个物种分类，剩余物种分类合并成other。

4.12 样本与物种关系图

4.12.1 分析方法

样本与物种的共现性关系图是一种描述样本与物种之间对应关系的可视化圈图，该图不仅反映了每个样本的优势物种组成比例，同时也反映了各优势物种在不同样本之间的分布比例。

4.12.2 结果说明

结果目录: **5_Taxonomic_Classification/circos/**

***/*_circos.pdf:** 样本与物种关系图

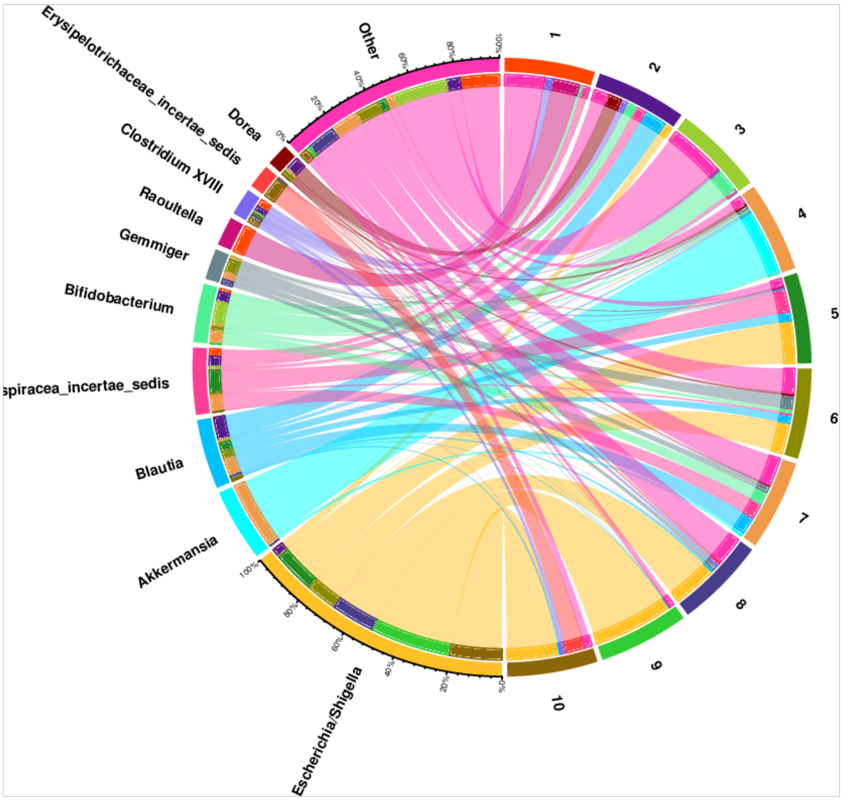

图12.1 genus水平上样本与物种关系图

样本与物种的共线性关系图中，右边半圆表示样本的物种丰度组成情况，左边半圆表示在该分类水平下物种在不同样本中的分布比例情况。圆圈从外到内：第一、二彩色圈：右半部分圆圈表示不同样本对应的物种组成，不同颜色表示不同物种，长度代表某一物种在该样本中的丰度比例 (第二圈内显示的百分比)；左半部分圆圈表示不同样本在优势物种中的分布比例，不同颜色表示不同样本，长度代表该样本在某一物种中的分布比例 (第二圈内显示的百分比)；第三圈：圈内的彩色条带，一端连接样本 (右边半圆)，条带端点宽度表示物种在该样本中的丰度，另一端连接物种 (左边半圆)，条带端点宽度表示该样本在相应物种中的分布比例，圈外数值表示相应物种的丰度数值。 (为了显示效果，仅显示前10个样本和丰度最高的前10个物种分类。)

4.13 单样品多级物种组成图

4.13.1 分析方法

单样品多级物种组成图可以将单个样本在域、门纲目等分类学水平的注释结果，通过多个同心圆由内向外直观地展现出来。

使用软件: **Krona**。

4.13.2 结果说明

结果目录: **5_Taxonomic_Classification/Krona/**

RDP.Krona.html: 样品多级物种组成图

下图仅为示例结果。

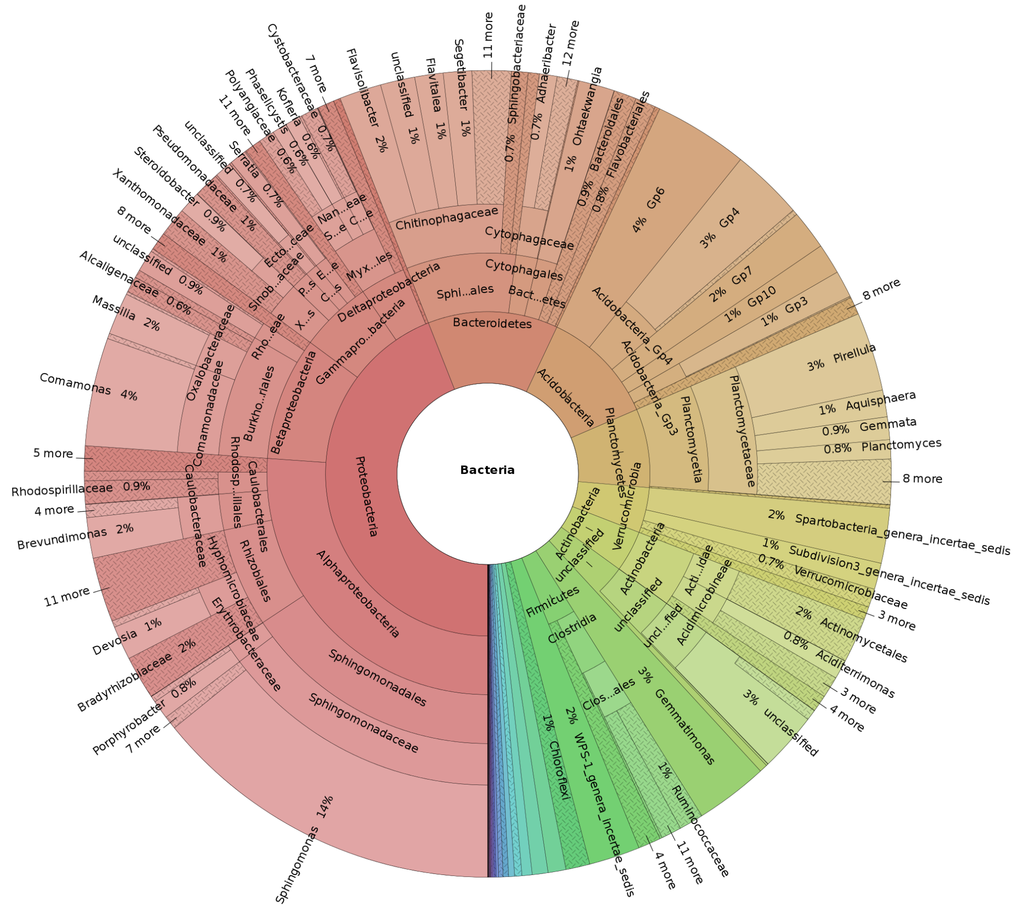

图13.1 单样品多级物种组成图

从最里圈往外圈看，依次为域、门、纲、目、科、属等水平的物种组成。(仅为示例图)

4.14 分类和系统发育信息可视化

4.14.1 分析方法

根据每个样本的分类学比对结果，选出优势物种的分类，结合物种丰度信息，以环状树状图显示。

使用软件: **GraPhlAn和iTOL**。

4.14.2 结果说明

结果目录: **5_Taxonomic_Classification**

GraPhlAn/*/*_graphlan_*.pdf: GraPhlAn绘制的分类和系统发育信息可视化图

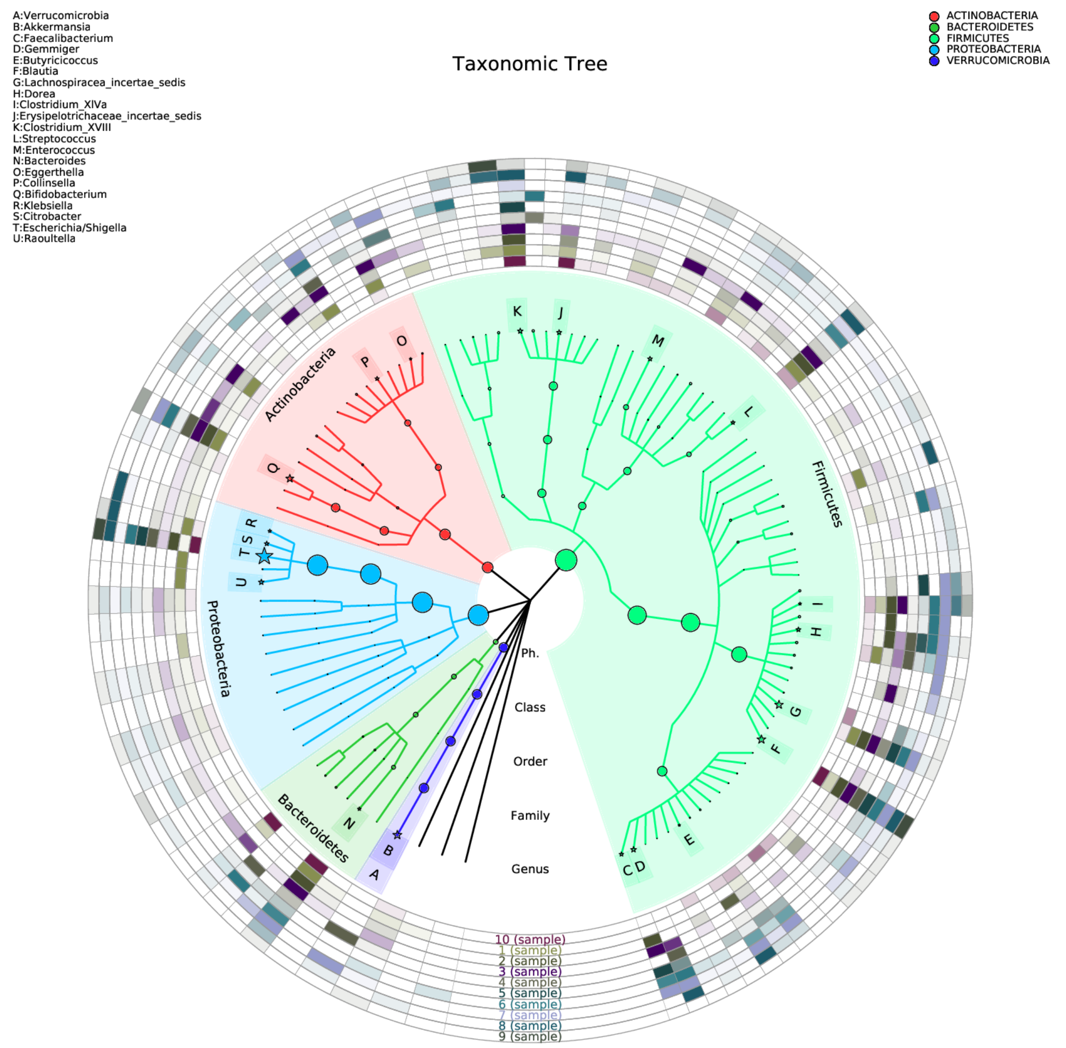

图14.1 GraPhlAn绘制的分类和系统发育信息可视化图
图居中的是丰度前100个物种进化分类树，并将丰度前20个物种 (以星号标出) 所对应的门按不同的颜色标出，圈和星号的大小代表丰度大小。外围环为热力图，每一环为一个样本(组)，每个样本对应一种颜色。颜色深浅随物种丰度变化

4.15 物种丰度热图

4.15.1 分析方法

Heatmap可以用颜色变化来反映群落分布的丰度信息，可以直观的将群落分布丰度值用定义的颜色深浅表示出来。同时将样品以及群落分布信息进行聚类并重新排布，将聚类之后的结果显示在heatmap中。因此可以很好的反映各分类水平上群落分布组成的异同。

软件：**R**的**gplots** package。

4.15.2 结果说明

结果目录: **5_Taxonomic_Classification/heatmap/**

***/*_heatmap_rainbow.pdf:** 所有样本物种丰度热图

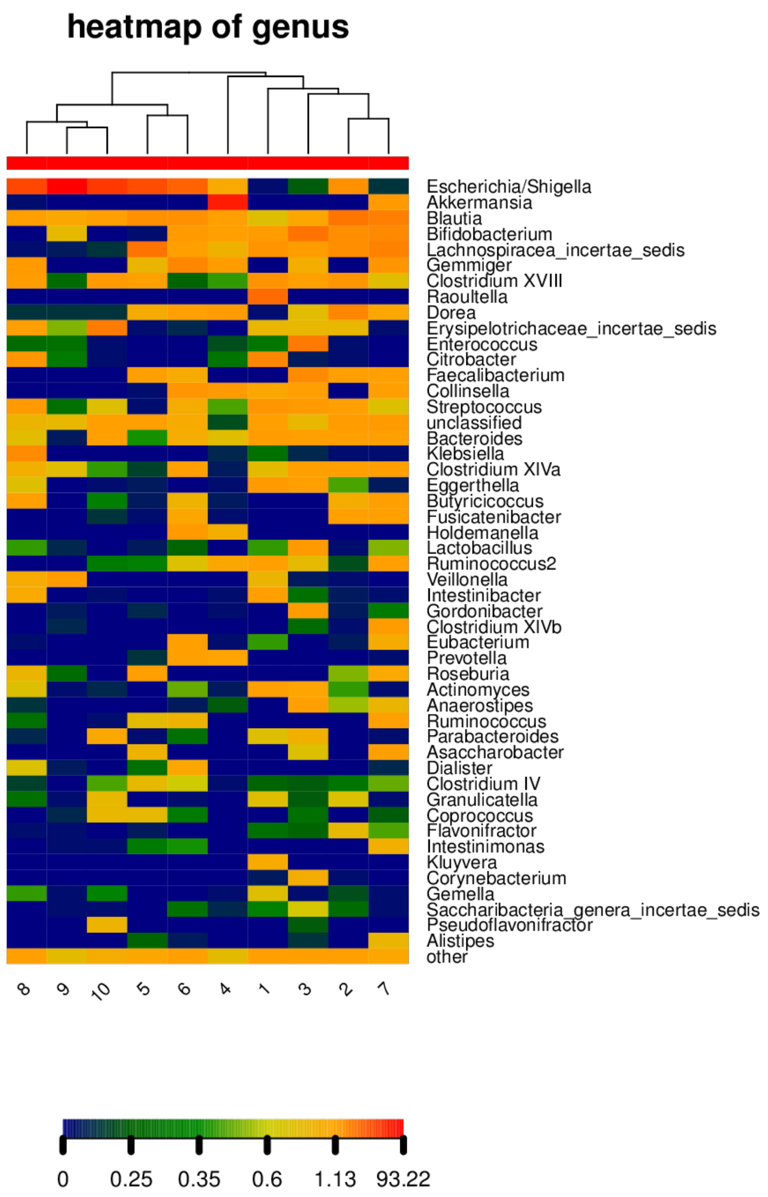

图15.1 genus水平物种丰度热图

物种丰度热图，用物种丰度矩阵绘制，图中每一列代表一个样本，行代表群落结构，颜色块代表相对物种丰度值，颜色越红表示相对丰度越高，颜色越蓝反之。另外热图对样本做了聚类，样本菌群分布越类似则样本距离越近，在图上方聚类树中的位置越靠近。为了展示效果，只显示丰度最高的前50个物种分类信息，剩余的物种分类合并成other，在图上的上方有颜色块，来自同一组的样本颜色相同。

4.16 物种丰度3D柱状图

4.16.1 分析方法

物种丰度3D图可以更立体的观察所有样本中群落的分布情况。

软件：**R的scatterplot3D** package。

4.16.2 结果说明

结果目录: **5_Taxonomic_Classification/ThreeD/**

***/*_plot_3D.pdf:** 所有样本物种丰度3D柱状图

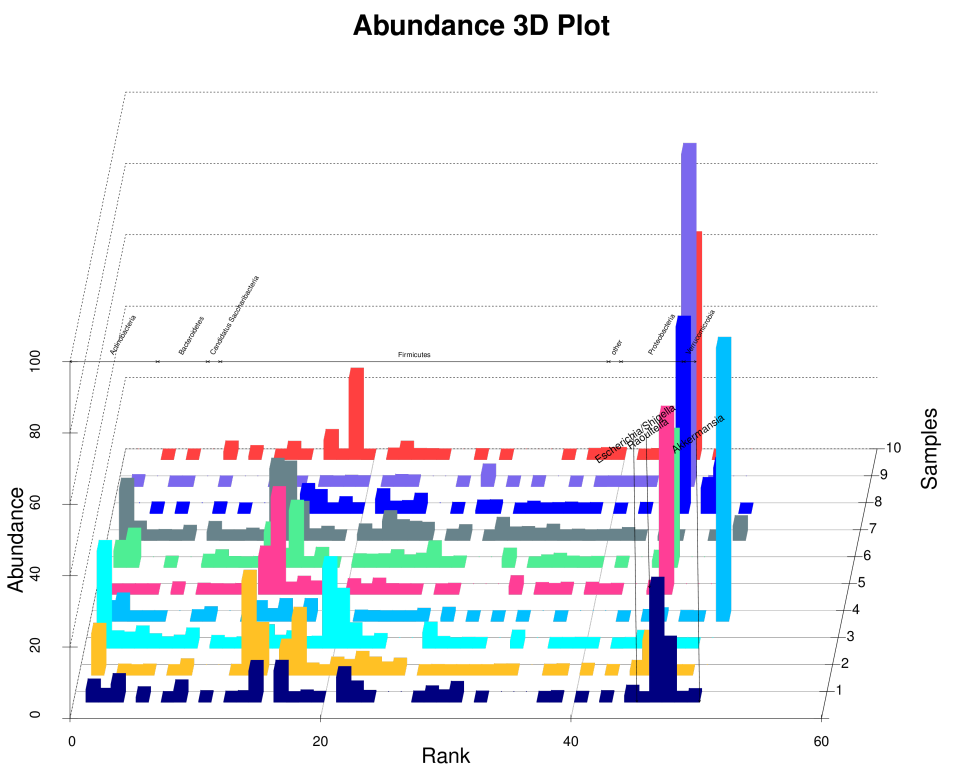

图16.1 genus水平上所有样本物种丰度3D柱状图
X轴代表菌落，Y轴代表丰度，Z轴代表样本。X轴上方的标签代表该群落的门水平，X轴物种的顺序是根据门水平进行排列。当物种的丰度水平在任意一个样本中超过阀值，会用箭头将此物种标记出来。

4.18 基于物种丰度样本聚类树图

4.18.1 分析方法

样本聚类树图可以通过树枝结构直观的反应出多个样品间的相似性和差异关系。首先根据beta多样性距离矩阵进行层次聚类 (Hierarchical cluatering) 分析，再使用非加权组平均法UPGMA (Unweighted pair group method with arithmetic mean) 算法构建树状结构，得到树状关系形式用于可视化分析。

软件: 使用**R**的**vegan** package根据各样本物种丰度计算beta多样性距离矩阵，计算样本间距离的方法为Bray-Curtis。

4.18.2 结果说明

结果目录: **5_Taxonomic_Classification/bray_crutis_tree/**

genus_RDP_NMDS_bray_crutis_tree.pdf: 所有样本在属水平上的聚类树图

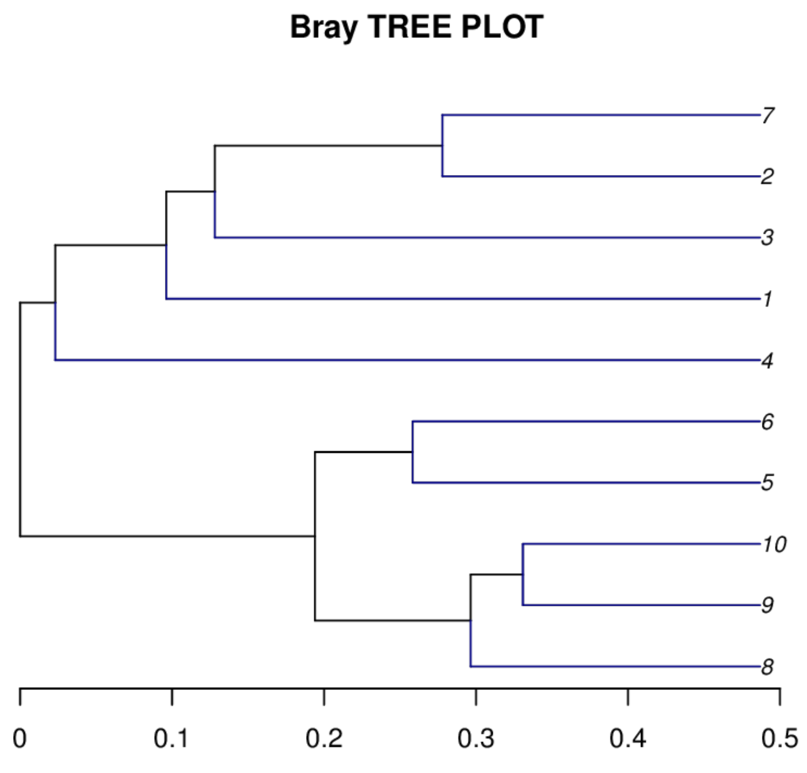

图18.1 genus水平上所有样本聚类树图
树枝的长度代表样本间的距离，越相似的样本会越靠近，图中同一颜色的树枝表示来源于同一组。

4.17 物种分类箱线图

4.17.1 分析方法

将不同层级水平上的多组样品的丰度进行四分位计算，比较不同样品丰度差异。同时根据与高层级的关系，绘制高层级的箱型图。

软件：**R**

4.17.2 结果说明

结果目录: **5_Taxonomic_Classification/boxplot/**

***/*_boxplot.pdf:** 物种分类箱线分析图。

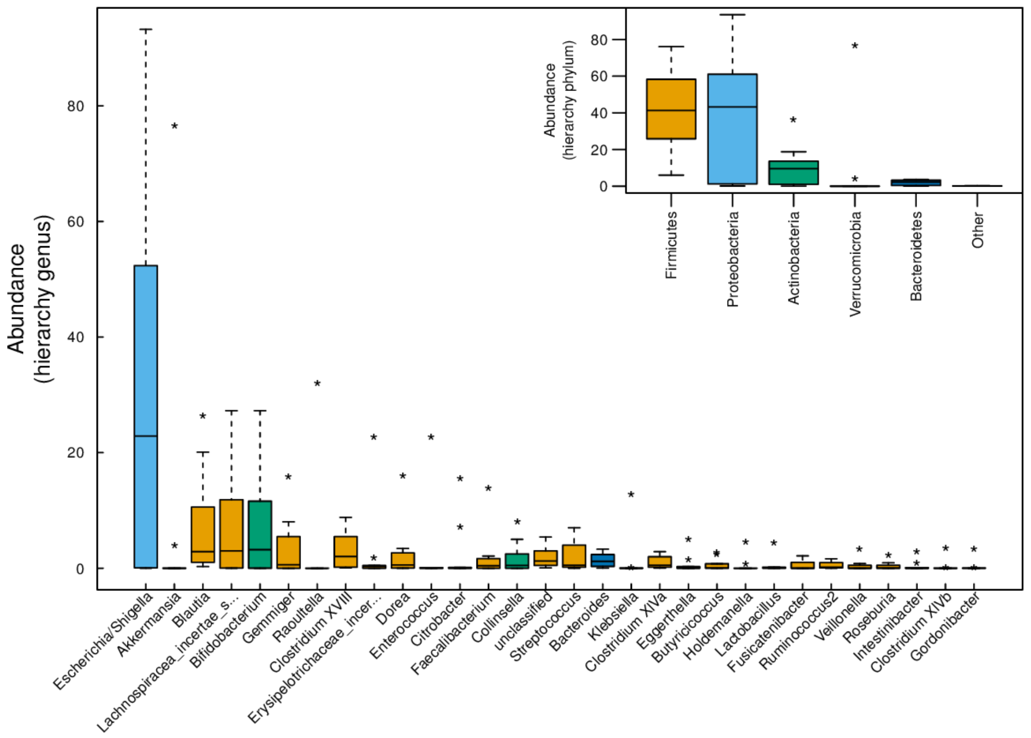

图17.1 物种分类箱线分析图

4.19 样品聚类树与柱状图组合分析

4.19.1 分析方法

将基于各样本物种丰度通过Bray-Curtis算法构建的样本聚类树与物种丰度柱状图结合起来，能够更加直观的看出样本间的关系及物种构成。

4.19.2 结果说明

结果目录: **5_Taxonomic_Classification/cluster_barplot/**

***/*_cluster_barplot.pdf:** 样品聚类树与柱状图组合分析图

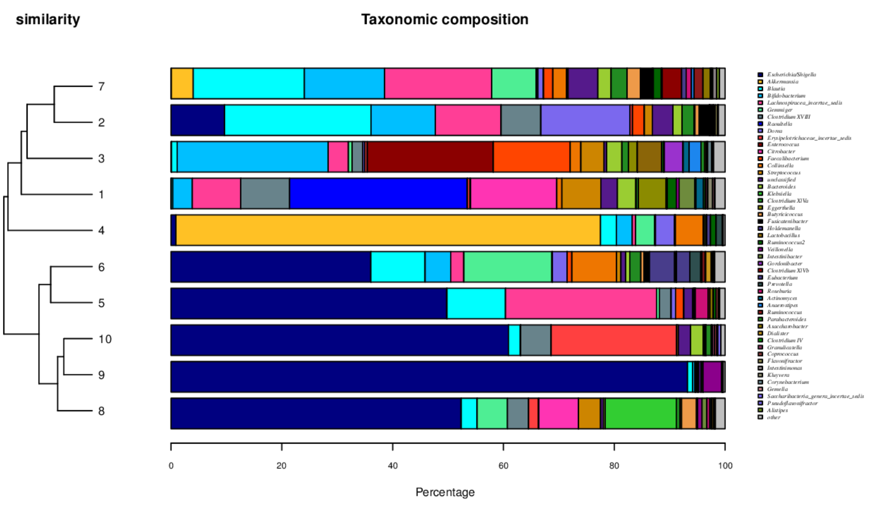

图19.1 genus水平上所有样品聚类树与柱状图组合分析图
图分为左、中、右三个部分。图左：基于Bray-Curtis的样本聚类树图；图中：根据聚类顺序排序的物种丰度柱状图。图右：物种的图示。

4.20 群落分类学系统组成树

4.20.1 分析方法

根据每个样本或多个样本的分类学比对结果，选出优势物种的分类，从整个分类系统上了解测序的环境样本中优势微生物的进化关系和丰度差异。

软件: **python的ete3** package。

4.20.2 结果说明

结果目录: **5_Taxonomic_Classification/ETE/**

***/*_ete3_*.pdf:** 单样本分类系统组成树状图

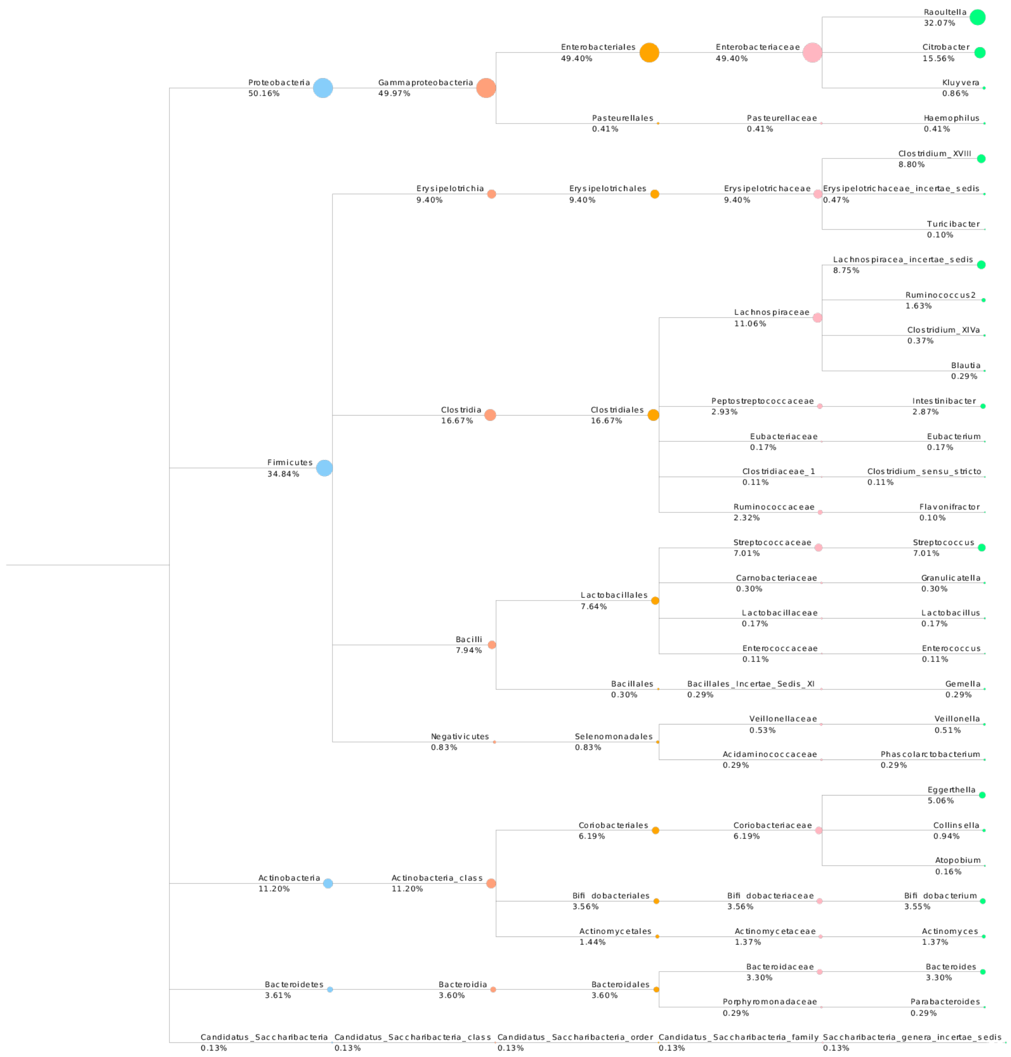

图20.1 genus水平上单样品分类系统组成树状图
图中不同颜色代表不同分类层级，从左至右依次为分类层级，圆圈大小代表物种丰度，支点附近分别为该分类名称和其对应丰度数值。最右侧为丰度前30的物种分布。

***/ALL_ete3_*.pdf:** 所有样本分类系统组成树状图

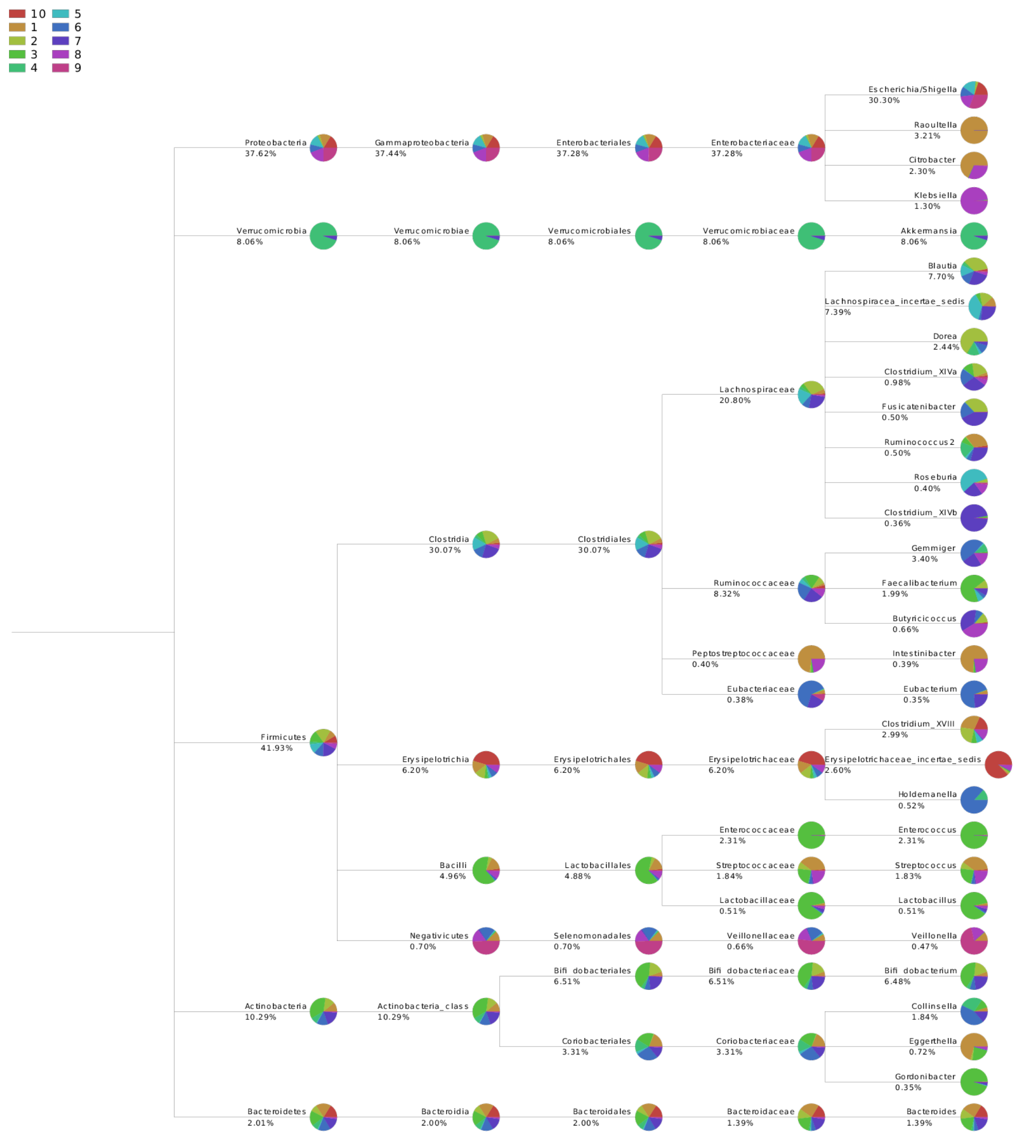

图20.2 genus水平上所有样品分类系统组成树状图

比对到不同样品在某分支上的丰度差异，通过带颜色的饼状图呈现。不同颜色代表不同的样品，颜色的扇形面积越大，说明在该分支上该样品的丰度越高。支点附近分别为该分类名称和其对应平均丰度数值。最右侧为丰度前30的物种分布。

4.21 PCA分析

4.21.1 分析方法

在多元统计分析中，主成分分析PCA (Principal Component Analysis) 是一种简化数据集的技术。主成分分析经常用于减少数据集的维数，同时保持数据集中对方差贡献最大的特征，从而有效地找出数据中最“主要”的元素和结构，去除噪音和冗余，将原有的复杂数据降维，揭示隐藏在复杂数据背后的简单结构。

软件：**R的vegan** package

4.21.2 结果说明

基于OTU的结果目录: **6_multi_dimension_analysis/OTU/PCA/**

OTU_PCA_3D.pdf: 所有样本基于OTU的PCA 3D图

OTU_PCA_PCA*_VS_PCA*.pdf: 所有样本基于OTU的PCA 2D图

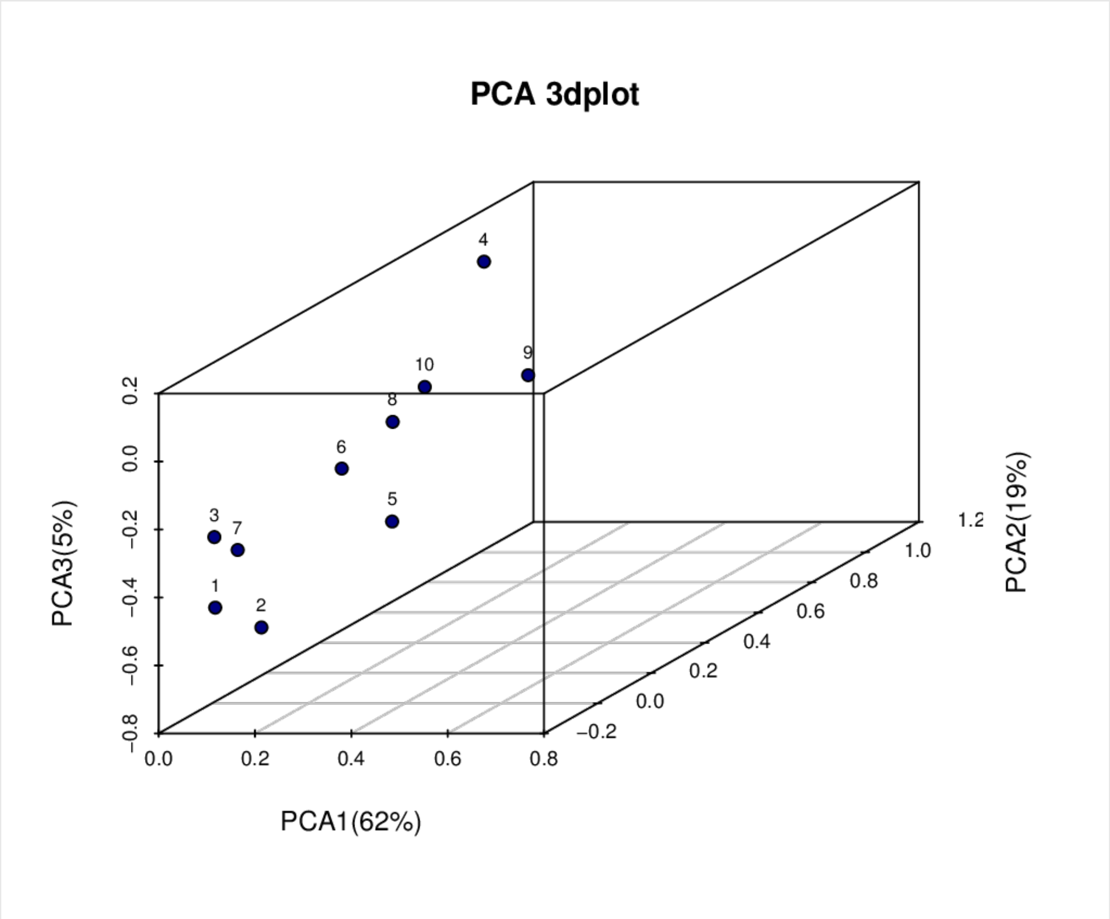

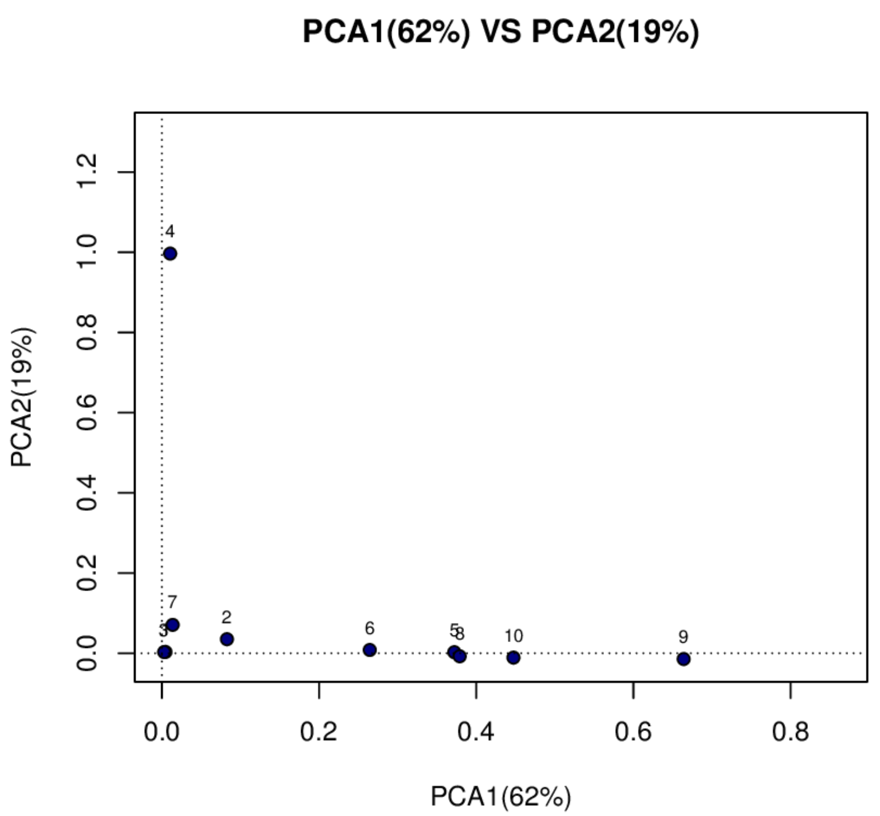

图21.1 基于OTU的PCA图
图中不同颜色代表不同样本或者不同的group中的样本，样本间相似度越高则在图中越聚集。

基于Taxonomy的结果目录: **6_multi_dimension_analysis/Taxonomy/PCA/**，文件格式同上

4.22 NMDS非度量多维尺度分析

4.22.1 分析方法

非度量多维尺度法是一种将多维空间的研究对象 (样本或变量) 简化到低维空间进行定位、分析和归类，同时又保留对象间原始关系的数据分析方法。适用于无法获得研究对象间精确的相似性或相异性数据，仅能得到他们之间等级关系数据的情形。其基本特征是将对象间的相似性或相异性数据看成点间距离的单调函数，在保持原始数据次序关系的基础上，用新的相同次序的数据列替换原始数据进行度量型多维尺度分析。换句话说，当资料不适合直接进行变量型多维尺度分析时，对其进行变量变换，再采用变量型多维尺度分析，对原始资料而言，就称之为非度量型多维尺度分析。其特点是根据样品中包含的物种信息，以点的形式反映在多维空间上，而对不同样品间的差异程度，则是通过点与点间的距离体现的，最终获得样品的空间定位点图。

软件：**R**的**vegan** package

4.22.2 结果说明

基于OTU的结果目录: **6_multi_dimension_analysis/OTU/NMDS/**

OTU_NMDS.pdf: 所有样本基于OTU的NMDS图

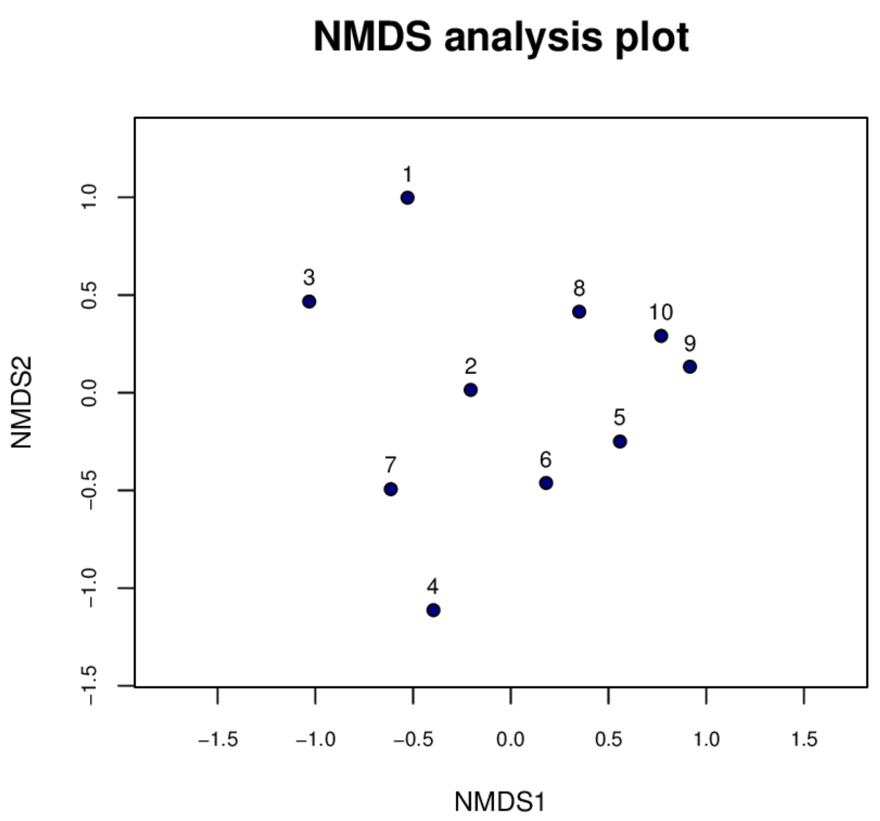

图22.1 基于OTU的NMDS图
图中不同颜色代表不同样本或者不同的group中的样本，样本间相似度越高则在图中越聚集。

基于Taxonomy的结果目录: **6_multi_dimension_analysis/Taxonomy/NMDS/**，文件格式同上

4.23 Network图

4.23.1 分析方法

网络 (Network) 图可以形象的展示不同样本或组之间物种的丰度情况，不同颜色代表不同样本，中间的交叉节点代表不同的物种或OTU，节点的面积代表物种或OTU丰度。当节点是物种时，采用门/纲/目/科/属分类信息进行绘图。分析时选取丰度高于 1%或丰度排序在前100位的物种或OTU信息。绘图时选取具有显著联系 (weight >= 100) 的节点绘制。

软件: 使用**QIIME**进行network分析，并使用**R**的**igraph** package进行绘图。

4.23.2 结果说明

基于OTU的结果目录: **6_multi_dimension_analysis/OTU/Network/**

OTU_network.kk.pdf: 基于OTU采用kamada kawai算法绘制的network图

OTU_network.rd.pdf: 基于OTU采用random算法绘制的network图

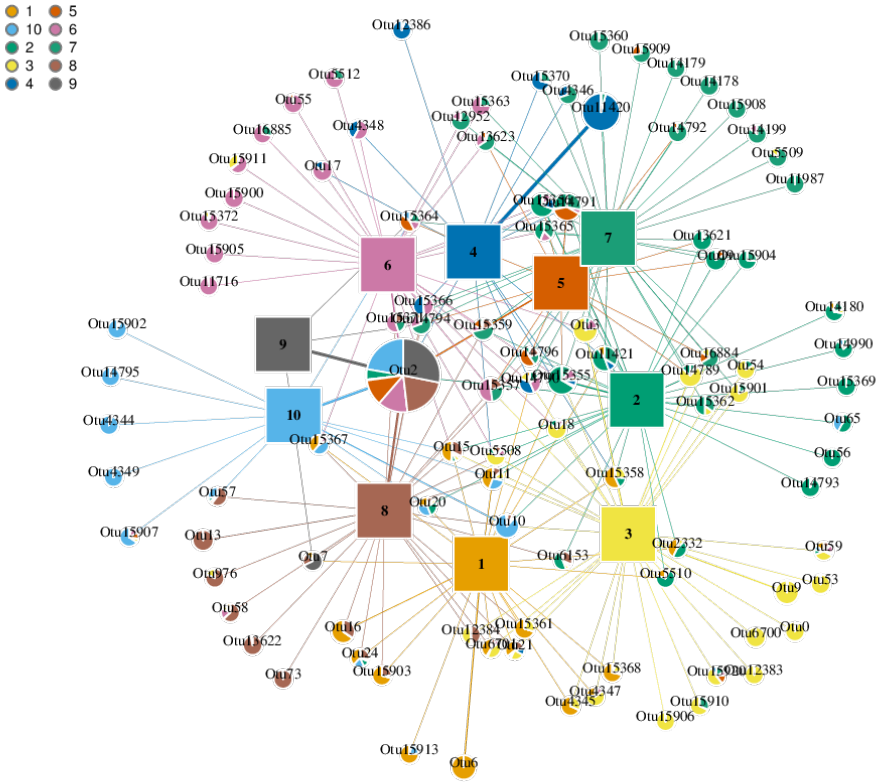

图23.1 基于OTU的network图
图中样本以矩形为标识，不同样本颜色不同，OTU或物种分类以饼图为标识，面积大小代表整体丰度大小，饼图中的不同色块代表不同样本在该OTU或物种分类上的丰度大小。

基于Taxonomy的结果目录: **6_multi_dimension_analysis/Taxonomy/Network/**，文件格式同上

4.24 微生物间相互关系分析图

4.24.1 分析方法

相关性分析是用于分析微生物间相互作用关系的经典方法，可甄别出微生物群落间具有显著相关性、强相关、正相关、负相关的各项。分析时选取丰度高于 1% 或丰度排序在前100 位的物种或OTU信息进行双侧检验。

软件: 使用**SparCC**计算群落/OTU间的相关性系数和p值，并使用**R**的**igraph** package绘制Network图，**corrplot** package绘制相关矩阵图。

4.24.2 结果说明

基于OTU的结果目录: **6_multi_dimension_analysis/OTU/**

Co-Network/OTU_co-network.kk.pdf: 基于OTU采用kamada kawai算法绘制的相关性分析network图

Co-Network/OTU_co-network.rd.pdf: 基于OTU采用random算法绘制的相关性分析network图

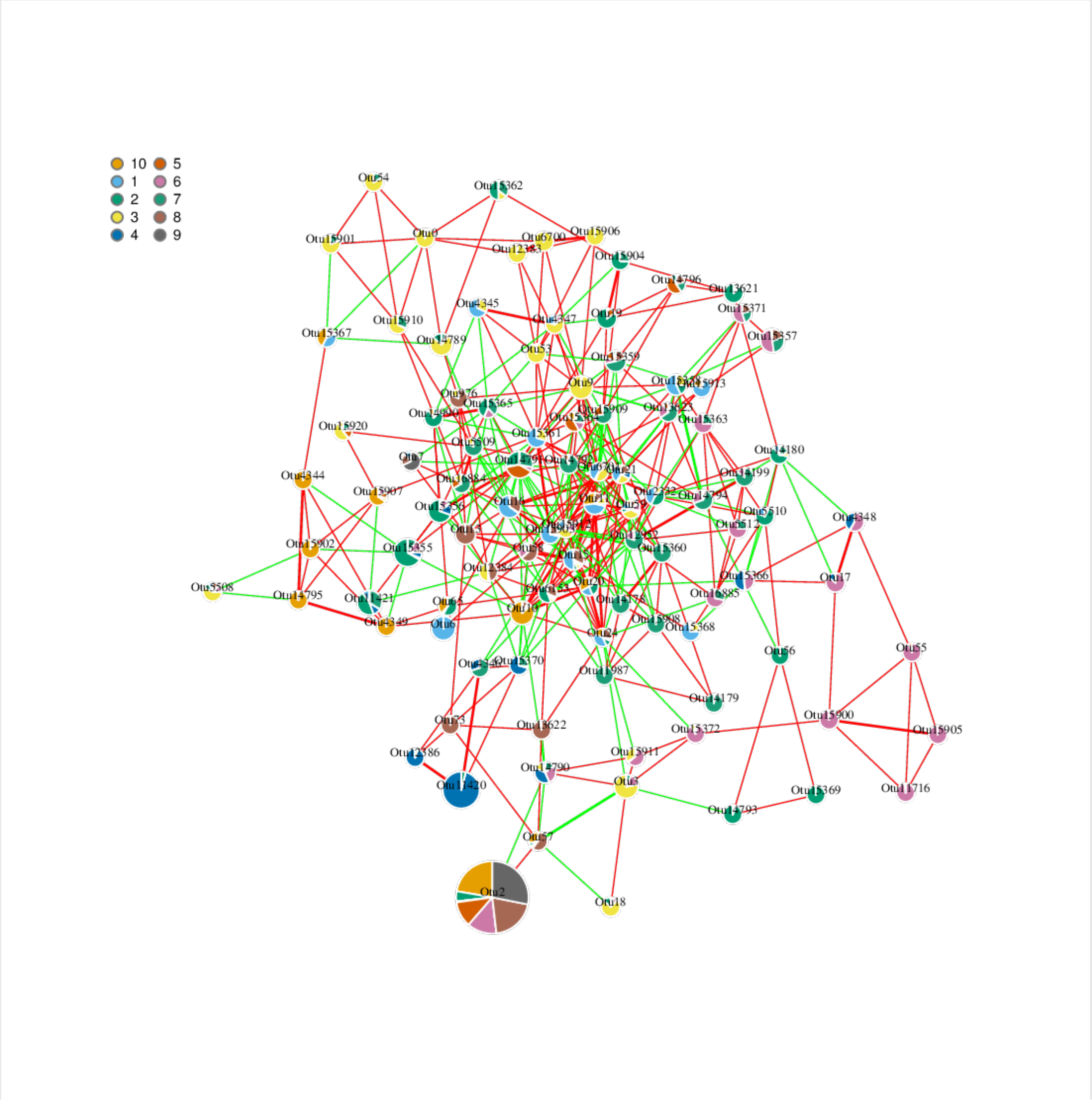

图24.1 基于OTU的相关性分析network图
图中饼图代表不同的物种或OTU，饼图中的不同色块代表不同样本在该OTU或物种分类上的丰度大小。在p值为0.01~0.05 时，相关性是显著的，用虚线表示。在p值小于 0.01 时，相关性是十分显著的，表明物种之间有强相关，用实线表示。相关系数为正数，表明物种之间为正相关，用红色线表示，负相关用绿线表示。当相关性系数大于0.8的时候，用粗线标识，当相关性系数小于0.8时，用细线表示。绘图时选取相关性系数大于0.6，p值小于0.05的节点绘制。

corrplot/OTU_corrlation.pdf: 基于OTU的相关矩阵图

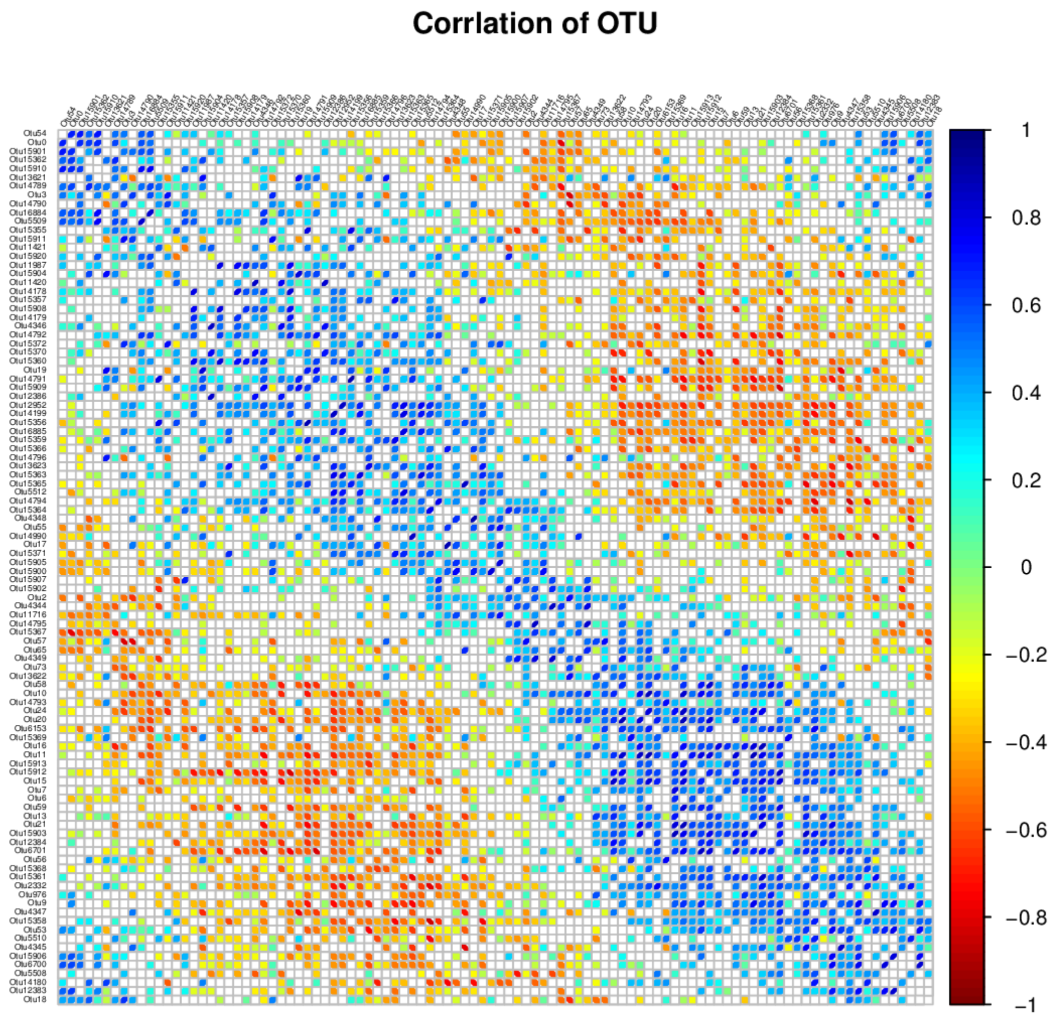

图24.2 基于OTU的相关矩阵图
图中椭圆大小代表相关性系数绝对值大小 (相关性绝对值越大，椭圆越小)，左斜的为正相关，右斜的为负相关，颜色随右侧色阶变化。图中仅显示p值小于0.05的结果。

基于Taxonomy的结果目录: **6_multi_dimension_analysis/Taxonomy/**，文件格式同上

4.25 系统发生进化树

4.25.1 分析方法

在分子进化研究中，系统发生的推断能够揭示出有关生物进化过程的顺序，了解生物进化历史和机制，可以通过某一分类水平上序列间碱基的差异构建进化树。

软件：选取OTU聚类结果中总体丰度最大的前50个OTU聚类的代表性序列和数据库中与这50个代表性序列种属信息一致且最长的序列，使用**MUSCLE**进行多序列比对得到alignment文件，采用**FastTree**根据最大似然法 (approximately-maximum-likelihood phylogenetic trees) 构建进化树。

4.25.2 结果说明

结果目录: **7_phylogenetic/OTU_repre/**

first50_tree_genus.pdf: 前50个OTU代表性序列与数据库序列系统发生进化树树状图

first50_tree_genus.circular.pdf: 前50个OTU代表性序列与数据库序列系统发生进化树环状图

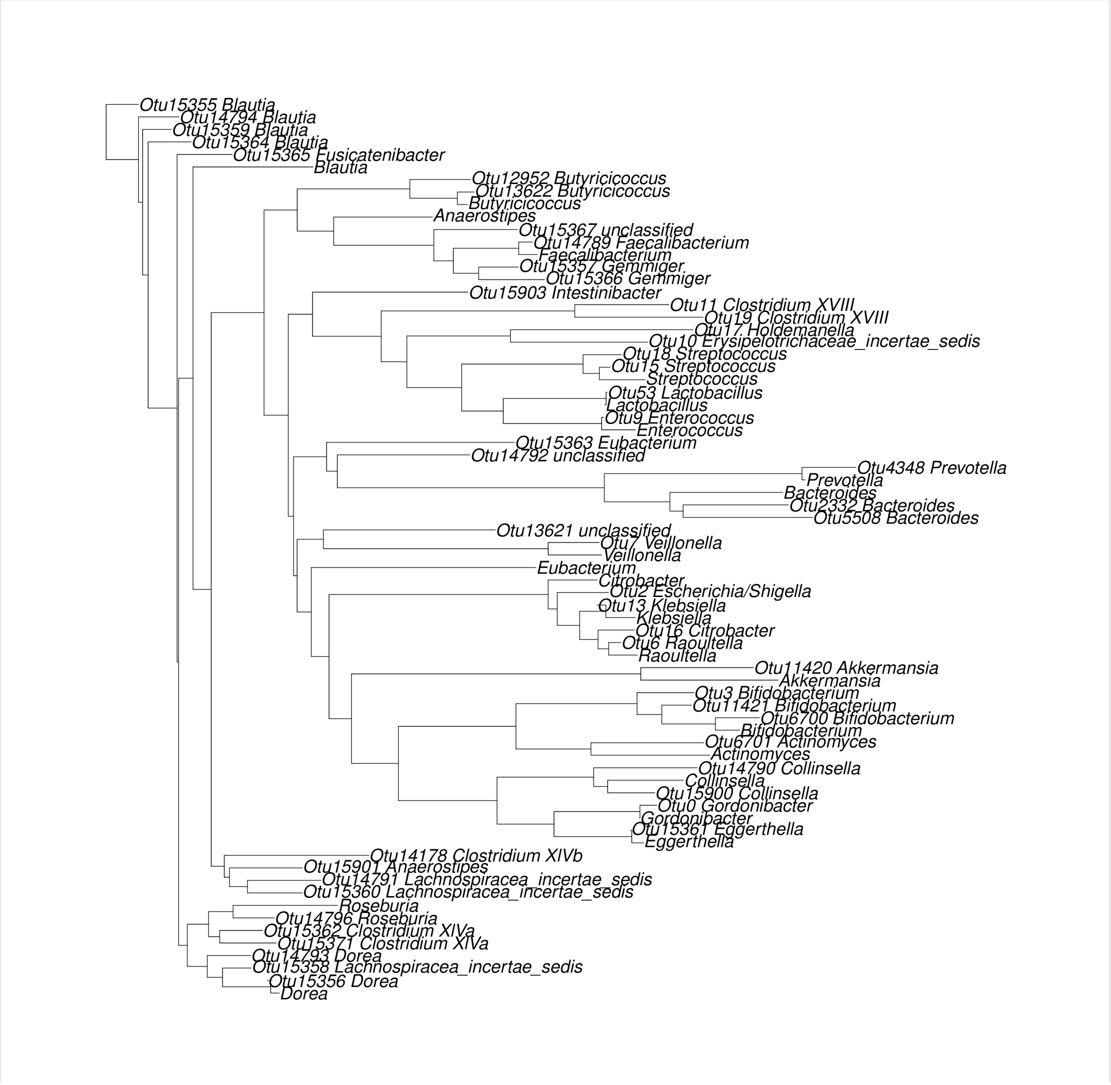

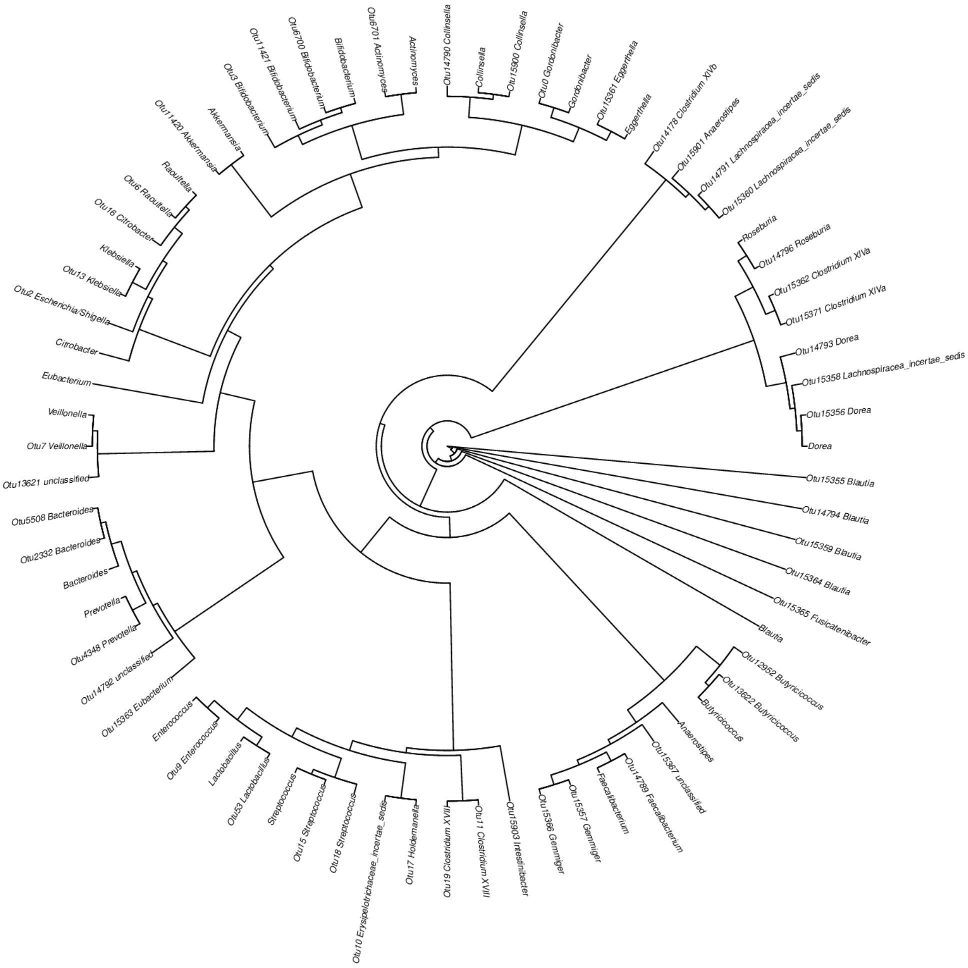

图25.1 前50个OTU代表性序列与数据库序列系统发生进化树结果图
前50个OTU代表性序列与数据库序列系统发生进化树结果树状图和环状图，使用genus水平信息绘制，有Otu标记的为OTU代表性序列结果，没有的为数据库对应序列的结果。

4.26 UniFrac分析

4.26.1 分析方法

UniFrac分析利用各样品序列间的进化信息来比较环境样品在特定的进化谱系中是否有显著的微生物群落差异。UniFrac可用于beta多样性的评估分析，即对样品两两之间进行比较分析，得到样品间的unifrac距离矩阵。其计算方法为：首先利用来自不同环境样品的OTU代表序列构建一个进化树，Unifrac度量标准根据构建的进化树枝的长度计量两个不同环境样品之间的差异，差异通过0-1距离值表示，进化树上最早分化的树枝之间的距离为1，即差异最大，来自相同环境的样品在进化树中会较大几率集中在相同的节点下，即它们之间的树枝长度较短，相似性高。如果两个环境较相似，则会共享不同的进化树枝，当所有树枝都被共享时，unifrac距离即为0。因为重复的序列不会影响进化树的树枝长度，所以unweighted unifrac度量方法没有计入不同环境样品的序列相对丰度，由于不同菌落的相对丰度可以更严格的描述群落的变化，使用weighted unifrac算法在计算树枝长度时将序列的丰度信息进行加权计算，因此unweighted unifrac可以检测样品间变化的存在，而weighted unifrac可以更进一步定量的检测样品间不同谱系上发生的变异。

软件：使用**MUSCLE**对所有OTU代表序列进行多序列比对得到alignment文件，采用**FastTree**根据最大似然法 (approximately-maximum-likelihood phylogenetic trees) 构建进化树，再使用**mothur**得到样品间距离矩阵。

4.26.2 结果说明

结果目录: **8_unifrac/(un)weighted**

(un)weighted_unifrac_distance_matrix.xls: 样品间的距离矩阵

表26.1 样品间的距离矩阵

	1	10	2	3
1	0.0	0.33	0.41	0.40
10	0.33	0.0	0.53	0.51
2	0.41	0.53	0.0	0.35
3	0.40	0.51	0.35	0.0

4.27 基于UniFrac的多样品相似度树分析

4.27.1 分析方法

Unifrac分析得到的距离矩阵可用于多种分析方法，通过层次聚类 (Hierarchical cluatering) 中的非加权组平均法UPGMA构建进化树等图形可视化处理，可以直观显示不同环境样品中微生物进化上的相似性及差异性。

UPGMA (Unweighted pair group method with arithmetic mean) 假设在进化过程中所有核苷酸/氨基酸都有相同的变异率，即存在着一个分子钟。通过树枝的距离和聚类的远近可以观察样品间的进化距离。

软件：**R**的**vegan** package

4.27.2 结果说明

结果目录: **8_unifrac/(un)weighted/sample_tree/**

(un)weighted_sample_tree.pdf: 样品进化树分析图

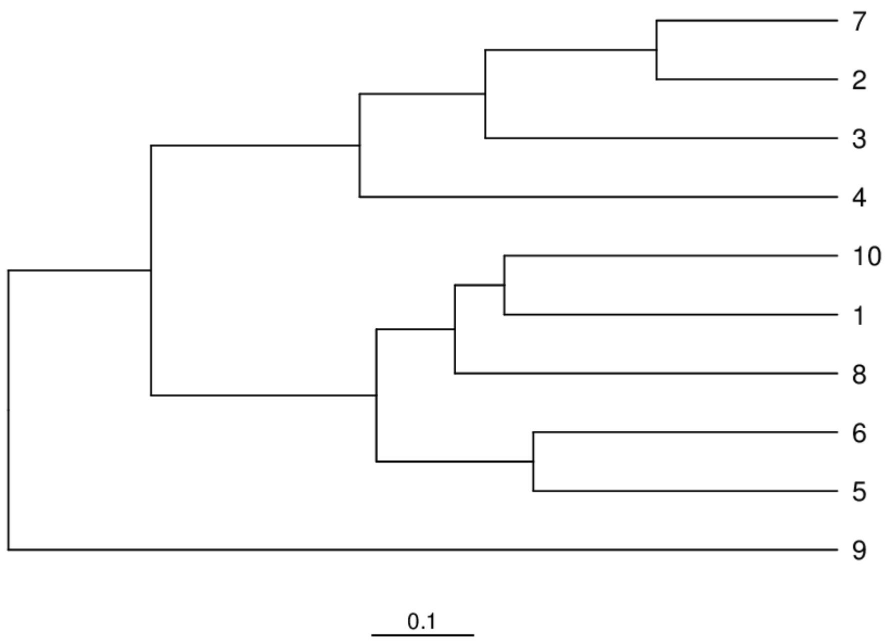

图27.1 样品进化树分析图
分支每一个点代表一个样本，长度值代表样本间距离值，样本相似度越高，则在树中距离越近。相同的group使用同样的颜色代表。

4.28 基于UniFrac的heatmap图

4.28.1 分析方法

距离热图能通过颜色直观的展现样本与样本之间的距离关系，即样本与样本之间的相似程度。

软件：采用**R**的**pheatmap** package根据样本间Unifrac距离矩阵绘制热图。

4.28.2 结果说明

结果目录: **8_unifrac/(un)weighted/heatmap/**

(un)weighted_heatmap.pdf: 样本距离热图

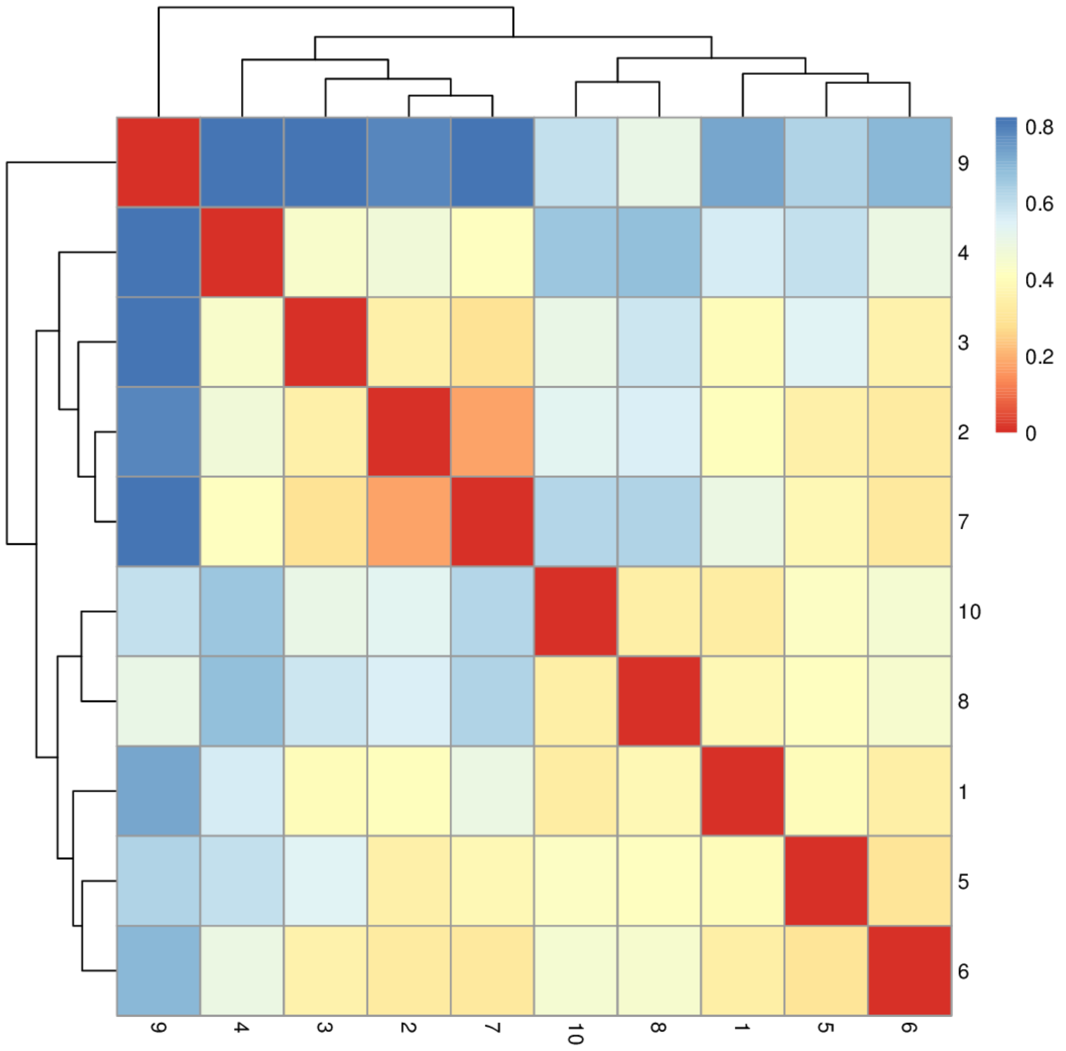

图28.1 样本距离热图
颜色块代表距离值，颜色越红表示样本间距离越近，相似度越高，越是越蓝则距离越远。热图中对样本间做了聚类，通过聚类树亦可看出样本间的距离关系。

4.29 基于UniFrac的PCoA分析

4.29.1 分析方法

PCoA (principal co-ordinates analysis) 是一种研究数据相似性或差异性的可视化方法，通过一系列的特征值和特征向量进行排序后，选择主要排在前几位的特征值，PCoA可以找到距离矩阵中最主要的坐标，结果是数据矩阵的一个旋转，它没有改变样品点之间的相互位置关系，只是改变了坐标系统。通过PCoA可以观察个体或群体间的差异。

软件：**R**的**vegan**的package

4.29.2 结果说明

结果目录: **8_unifrac/(un)weighted/pcoa/**

(un)weighted_pcoa_3D.pdf: unifrac PCoA 3D图

(un)weighted_pcoa_PCoA*_VS_PCoA*.pdf: unifrac PCoA 2D图

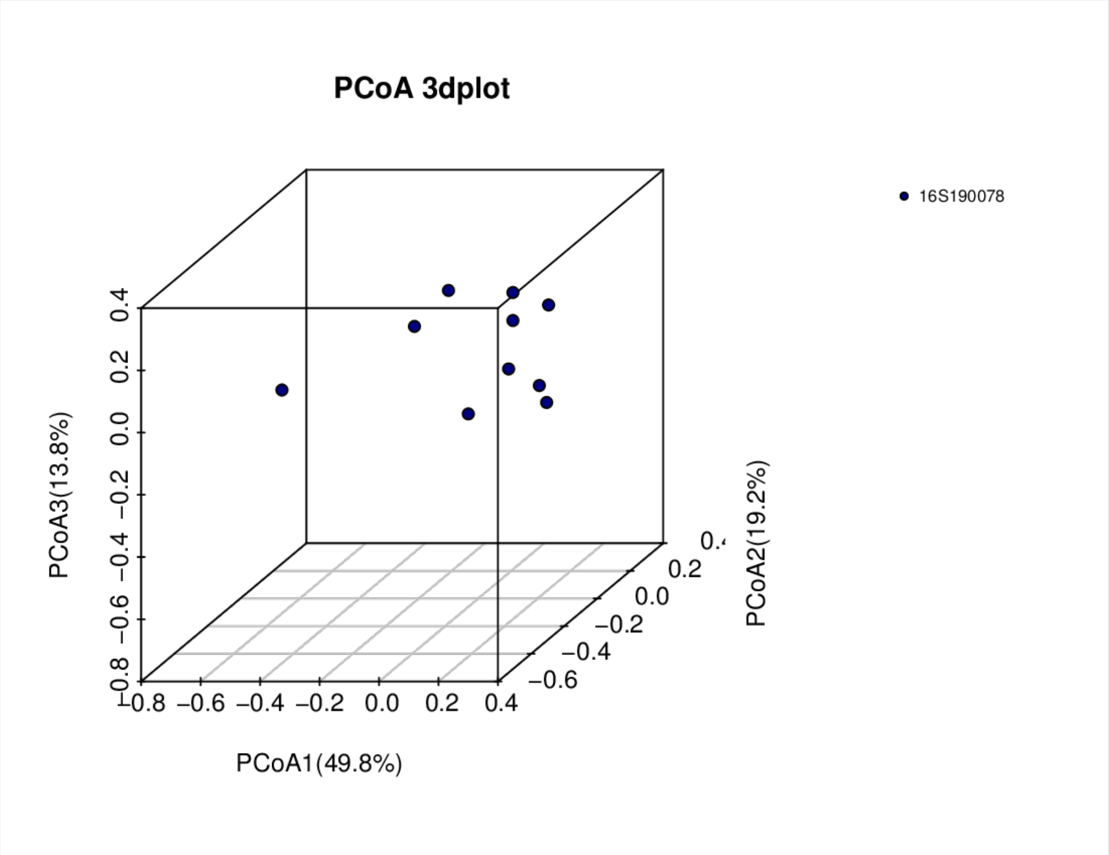

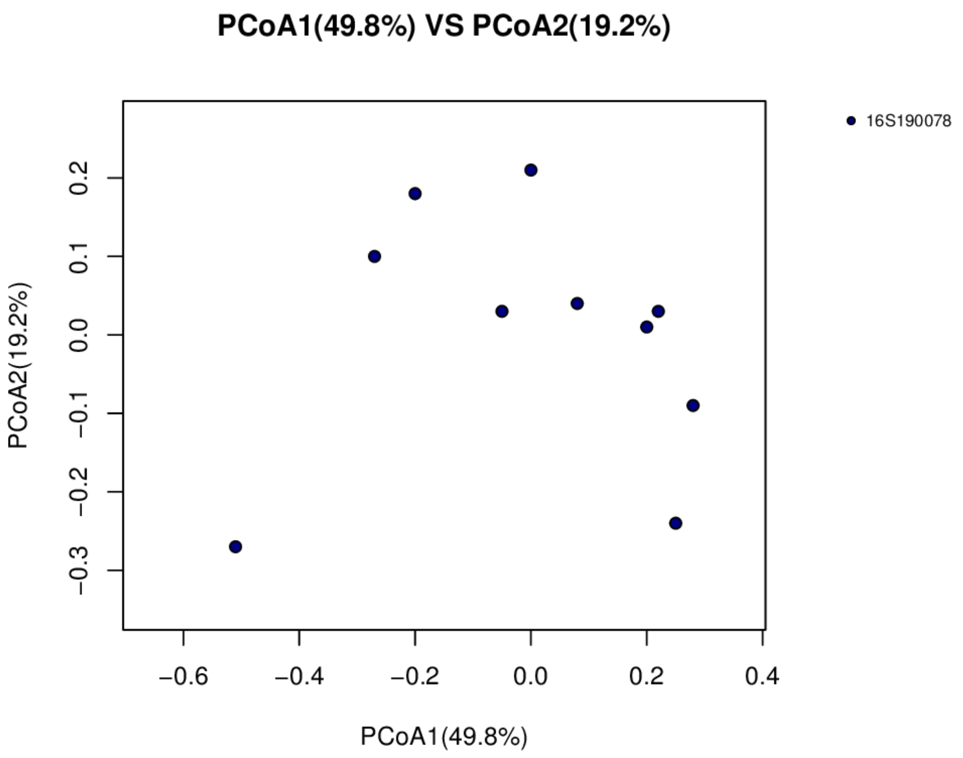

图29.1 unifrac PCoA图
基于unifrac距离矩阵的PCoA 3D图和2D图。不同颜色的点代表不同group中的样本，样本间相似度越高则在图中越聚集，反之样本间相似度越低则在空间距离越远。

4.30 UniFrac距离箱式图

4.30.1 分析方法

将不同分类或环境的多组样品的距离进行四分位计算，比较不同样品组的组内和组间的距离分布差异。

箱线图 (Boxplot) 也称箱须图 (Box-whisker Plot)，是利用数据中的五个统计量：最小值、第一四分位数、中位数、第三四分位数与最大值来描述数据的一种方法，它也可以粗略地看出数据是否具有对称性，分布的分散程度等信息，特别可以用于对几个样本的比较。 简单箱线图由五部分组成，分别是最小值、中位数、最大值和两个四分位数。

同一数轴上，几批数据的箱形图并行排列形成箱式图，可以识别数据异常值；粗略估计和判断数据特征；比较几批数据的形状，使几批数据的中位数、尾长、异常值、分布区间等形状信息一目了然。

软件：**R**

4.30.2 结果说明

结果目录: **8_unifrac/(un)weighted/boxplot/**

(un)weighted_boxplot.pdf: unifrac 箱线分析图

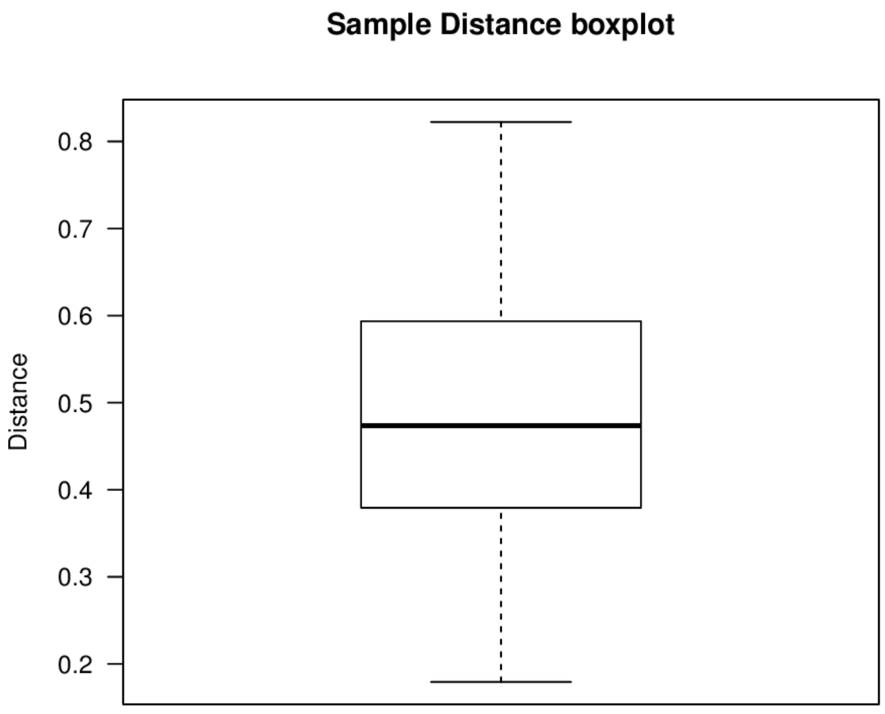

图30.1 unifrac 箱线分析图
第一四分位数 (Q1)，又称“下四分位数”，等于该样本中所有数值由小到大排列后第25%的数字。第二四分位数 (Q2)，又称“中位数”，等于该样本中所有数值由小到大排列后第50%的数字。 第三四分位数 (Q3)，又称“上四分位数”，等于该样本中所有数值由小到大排列后第75%的数字。

4.31 样本间菌群丰度差异分析

4.31.1 分析方法

基于物种分类结果，计算在不同水平上各rank的丰度，比较样本或组间丰度差异，找出样本或组间丰度存在显著差异的物种分类,默认只做门和属的水平，筛选条件为P<=0.05。

当比较对象为样本时，采用fisher exact test；当比较对象为组时，采用Welch’s t-test。最后将检验得到的pvalue值采用FDR做Multiple test correction得到qvalue值。

使用软件: **STAMP**。

4.31.2 结果说明

结果目录: **9_Different/Abu_Diff/**

***/diff_*_reads.*.vs.*.xls:** 两两差异比较文件

表31.1 两两样品差异比较结果

	Freq1	Freq2	pValue	qValue	Effect Size	95.0% lower CI	95.0% upper CI
Escherichia/Shigella	5.5e-03	60.91	0.0	0.0	-60.90	-61.29	-60.51
Streptococcus	7.01	0.29	0.0	0.0	6.71	6.49	6.93
Citrobacter	15.56	0.01	0.0	0.0	15.55	15.24	15.85
Intestinibacter	2.87	0.01	0.0	0.0	2.86	2.72	3.00
Erysipelotrichaceae incertae sedis	0.47	22.66	0.0	0.0	-22.19	-22.53	-21.85
Raoultella	32.07	0.0	0.0	0.0	32.07	31.68	32.47
Eggerthella	5.06	5.1e-03	0.0	0.0	5.06	4.88	5.24
Lachnospiracea incertae sedis	8.75	0.04	0.0	0.0	8.71	8.47	8.95
Bifidobacterium	3.55	0.0	0.0	0.0	3.55	3.40	3.71
Actinomyces	1.37	0.03	5.8e-211	1.2e-209	1.34	1.24	1.44
Blautia	0.29	2.15	4.2e-200	8.2e-199	-1.86	-1.99	-1.74
Ruminococcus2	1.63	0.12	2.9e-199	5.3e-198	1.51	1.40	1.62
Collinsella	0.94	0.0	1.4e-165	2.3e-164	0.94	0.86	1.02
Kluyvera	0.86	0.0	1.7e-150	2.6e-149	0.86	0.78	0.93

***/diff_*_reads.*.vs.*.P0.05.xls:** P值检验显著的两两差异统计文件

***/diff_*_reads.*.vs.*.ExtendErrorBar.pdf:** 差异比较的误差线图

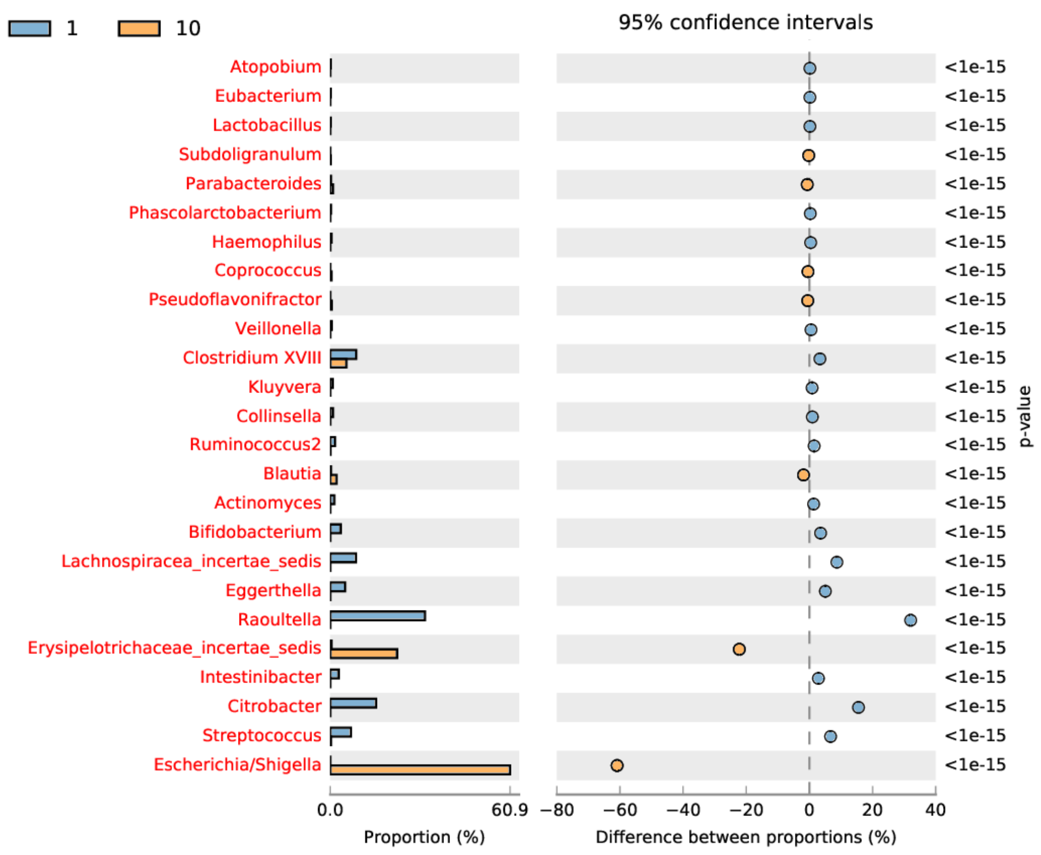

图31.2 差异比较的误差线图
左图所示为不同物种分类在两个样品 (组) 中的丰度比例，中间所示为95%置信度区间内，物种分类丰度的差异比例，最右边的值为p 值，p 值 < 0.05，表示差异显著，物种分类用红色标识。(仅列出p值最低的25个)

4.32 Ternary Plot图

4.32.1 分析方法

Ternary Plot是用一个三角形描述三个变量之间不同属性的比率关系，在分析中可以根据物种分类或功能信息对三个样品的物种或功能组成进行比较分析，通过三角图可以直观的显示出不同物种或功能在样品中的比重和关系。

使用软件: **R**的**ggtern** package。

4.32.2 结果说明

结果目录: **9_Different/Ternaryplot/**

***_*_*.Ternaryplot.pdf:** Ternary Plot图。

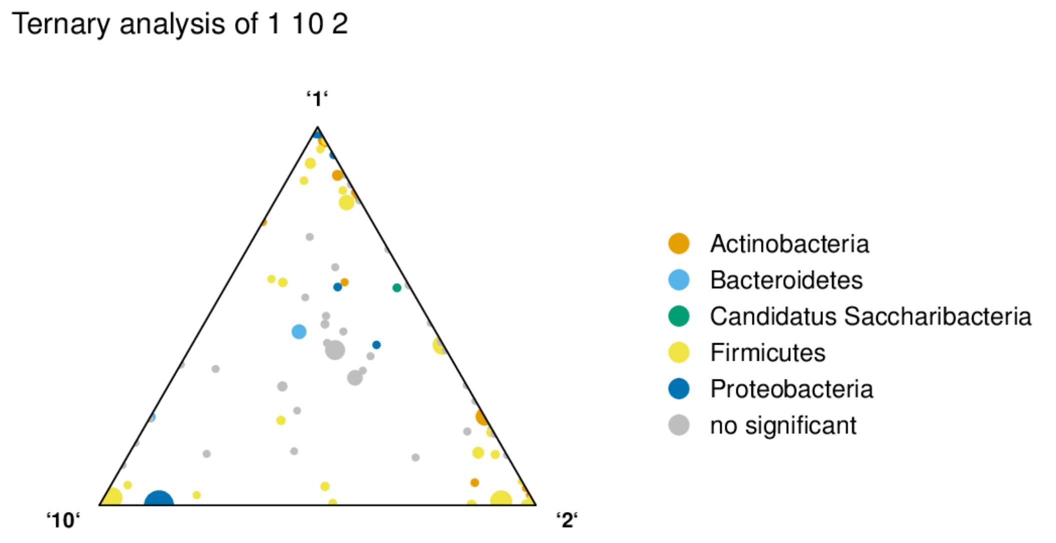

图32.1 Ternary Plot图
三个角分别代表三个样本，圆圈代表不同物种，彩色圆圈代表在下面两角样本中显著富集的物种 (相对于顶角样本) 。灰色圆圈代表非显著富集物种，圆圈大小代表物种的平均相对丰度。

4.33 Adonis/PERMANOVA分析

4.33.1 分析方法

Adonis 又称置换多因素方差分析（permutational MANOVA）或非参数多因素方差分析（nonparametric MANOVA），是一种对半度量或度量距离矩阵的离差平方和进行区分的非参数统计学方法。它利用半度量(如 Bray-Curtis) 或度量距离矩阵(如 Euclidean)对总方差进行分解，分析不同分组因素对样品差异的解释度，并使用置换检验对划分的统计学意义进行显著性分析。

软件：**R**的**vegan**的package。

4.33.2 结果说明

结果目录: **9_Different/Adonis/**

***/*_PERMANOVA.xls:** Adonis分析结果表格文件

表33.1 Adonis分析结果

comparison: 组间比较名称。
F.Model ：F检验值。
R2：表示不同分组对样品差异的解释度，即分组方差与总方差的比值，R2越大表示分组对差异的解释度越高。
P Value：表示P值，小于0.05说明本次检验的可信性度高。

4.34 PICRUSt功能分析

4.34.1 分析方法

通过对已有测序微生物基因组的基因功能的构成进行分析后，我们可以通过16s测序获得的物种构成推测样本中的功能基因的构成，从而分析不同样本和分组之间在功能上的差异。

通过对宏基因组测序数据功能分析和对应16s预测功能分析结果的比较发现，此方法的准确性在84%-95%，对肠道微生物菌群和土壤菌群的功能分析接近95%，能非常好的反映样品中的功能基因构成。

使用软件：**PICRUSt**。

4.34.2 结果说明

基于COG的结果目录: **10_Function/COG/PICRUSt**

COG.abundance.reads.xls: COG基因预测结果

表34.1 COG基因预测结果

	10	1	2	3	4
COG0393	13003	17369	28676	12376	3799
COG4055	0	0	0	0	0
COG2043	14	7	23	1904	17
COG3010	21539	20593	31968	14107	6047
COG3011	22	183	170	145	27057

	10	1	2	3	4
COG3012	10506	18476	11960	4059	1289
COG3013	5170	11794	1059	4512	177
COG3014	0	45	0	9	7
COG3015	5285	12147	1313	409	204
COG3016	13	389	149	180	17
COG3017	5182	11867	1083	106	218
COG3018	0	37	1	2	3
COG3019	37	154	71	237	23
COG0390	9998	11931	4507	9501	883

从左至右依次为预测得到的COG ID、各样本的丰度以及COG功能详细信息

COG.abundance.normalreads.xls: COG基因预测均一化后结果

基于KEGG的结果目录: **10_Function/KEGG/PICRUSt**，文件格式同上。

4.35 功能组分图

4.35.1 分析方法

根据功能预测结果，可以得知一个或多个样品在功能更高层级水平上的情况，观测样品在更高层级水平上的功能结构。

软件：利用**R**对功能统计结果进行作图。

4.35.2 结果说明

基于COG的结果目录: **10_Function/COG/**

barplot/COG_barplot.pdf: 所有样本功能结构分布柱状图

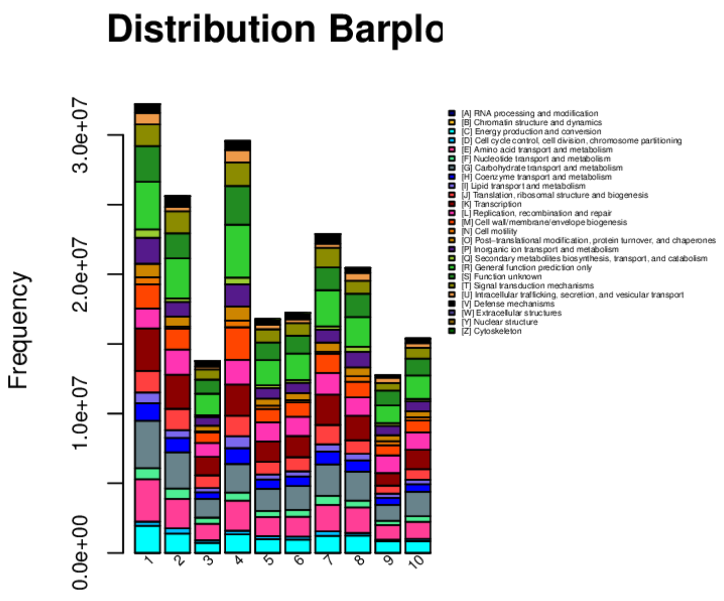

图35.1 基于COG的功能结构分布柱状图
横轴为各样品的编号，纵轴为丰度比例。颜色对应各功能名称，不同色块宽度表示不同功能丰度。

horiz_bar/*_COG_horiz_bar.pdf: 单样本功能丰度条形图

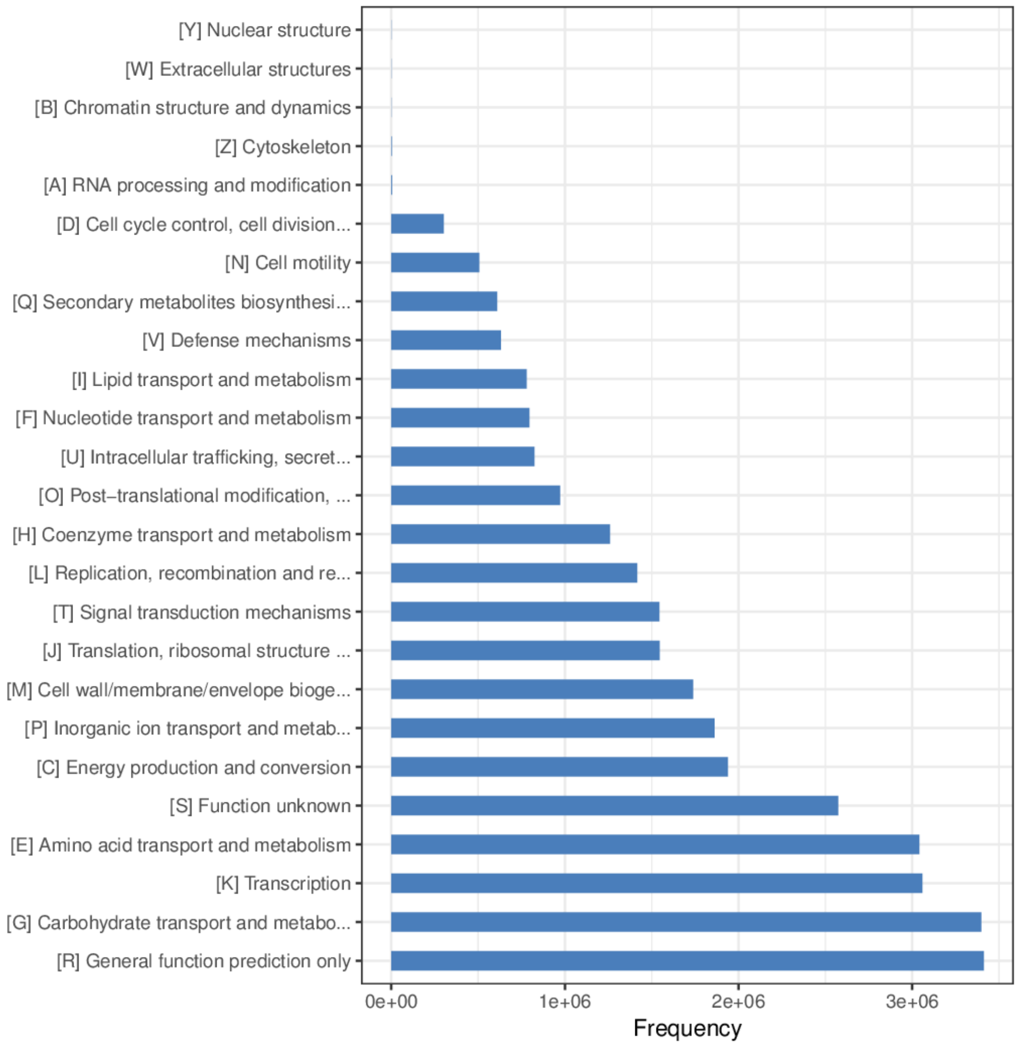

图35.2 基于COG功能分类丰度单样本条形图
图中展示了样本功能分类的分布情况，并按照总体丰度从大到小排序。

基于KEGG的结果目录: **10_Function/KEGG/**，文件格式同上。

4.36 基于功能丰度的样本聚类图

4.36.1 分析方法

样本聚类树图可以通过树枝结构直观的反应出多个样品间的相似性和差异关系。首先根据beta多样性距离矩阵进行层次聚类 (Hierarchical cluatering) 分析，再使用非加权组平均法UPGMA (Unweighted pair group method with arithmetic mean) 算法构建树状结构，得到树状关系形式用于可视化分析

软件: 使用**R**的**vegan** package根据各样本功能丰度计算beta多样性距离矩阵，计算样本间距离的方法为Bray-Curtis。

4.36.2 结果说明

基于COG的结果目录: **10_Function/COG/bray_crutis_tree/**

COG_bray_crutis_tree.phylo: 基于COG的样本聚类树作图数据

COG_bray_crutis_tree.pdf: 基于COG的样本聚类树图

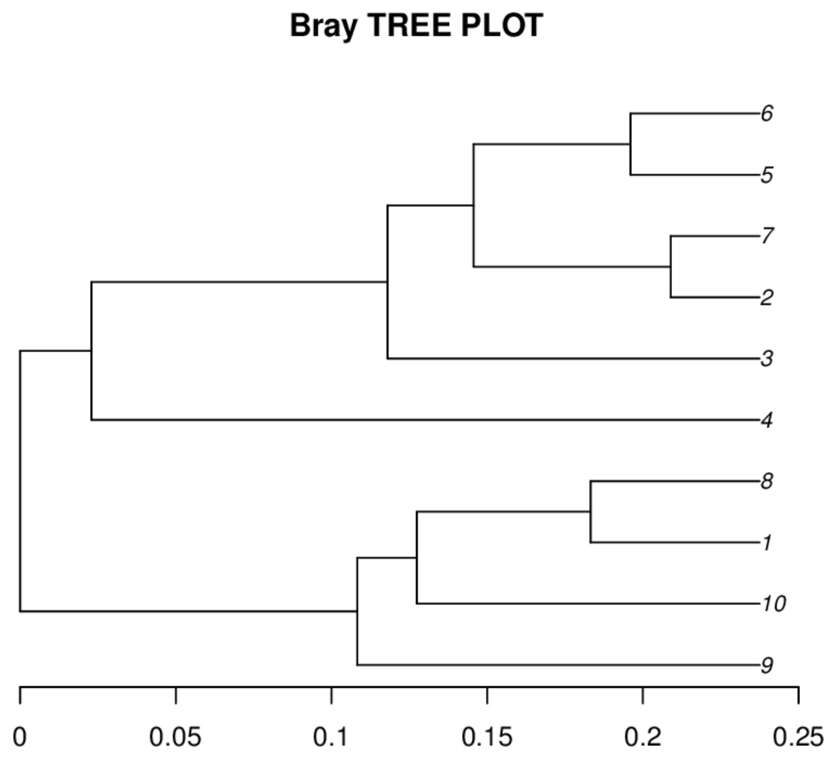

图36.1 基于COG的样本聚类树图
树枝的长度代表样本间的距离，越相似的样本会越靠近，图中同一颜色的树枝表示来源于同一组。

基于KEGG的结果目录: **10_Function/KEGG/bray_crutis_tree/**，文件格式同上。

4.37 功能丰度热图

4.37.1 分析方法

Heatmap可以用颜色变化来反映功能的丰度信息，可以直观的将功能丰度值用定义的颜色深浅表示出来。同时将样品以及功能信息进行聚类并重新排布，将聚类之后的结果显示在heatmap中。

软件：**R**的**gplots** package。

4.37.2 结果说明

基于COG的结果目录: **10_Function/COG/heatmap/**

COG_heatmap_rainbow.pdf: 所有样本功能丰度热图

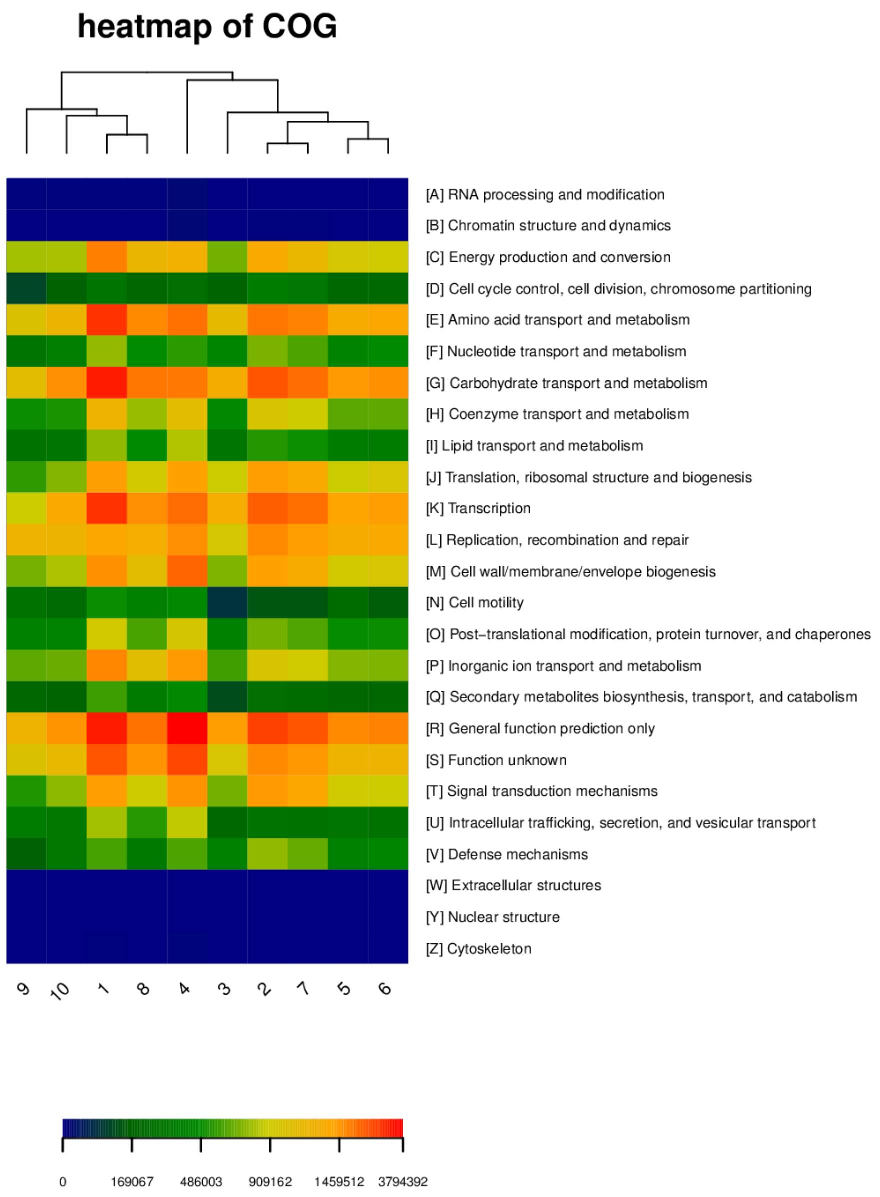

图37.1 基于COG的功能丰度热图
功能丰度热图，用功能丰度矩阵绘制，图中每一列代表一个样本，行代表功能，颜色块代表功能丰度值，颜色越红表示丰度越高，颜色越蓝反之。另外热图对样本做了聚类，样本菌群分布越类似则样本距离越近，在图上方聚类树中的位置越靠近。在图上的上方有颜色块，来自同一组的样本颜色相同。

基于KEGG的结果目录: **10_Function/KEGG/heatmap/**，文件格式同上。

4.38 样品聚类树与功能柱状图组合分析

4.38.1 分析方法

将基于各样本功能丰度通过Bray-Curtis算法构建的样本聚类树与功能丰度柱状图结合起来，能够更加直观的看出样本间的关系及功能构成。

4.38.2 结果说明

基于COG的结果目录: **10_Function/COG/cluster_barplot/**

COG_cluster_barplot.pdf: 样品聚类树与柱状图组合分析图

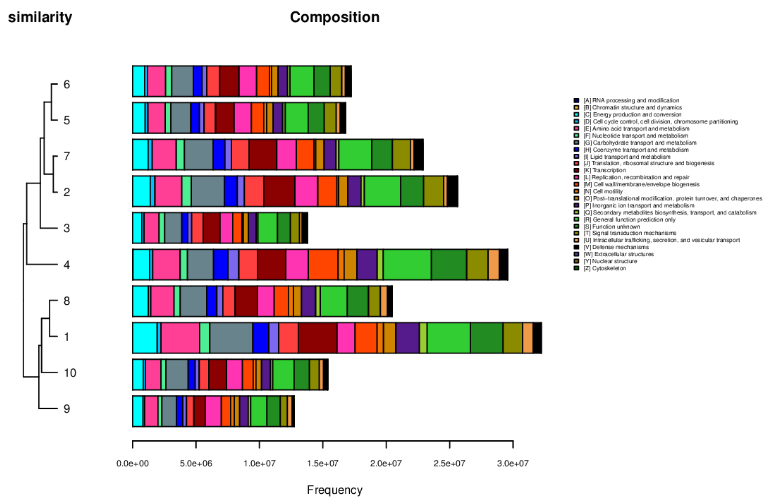

图38.1 基于COG的所有样品聚类树与柱状图组合分析图
图分为左、中、右三个部分。图左：基于Bray-Curtis的样本聚类树图；图中：根据聚类顺序排序的功能丰度柱状图。图右：物种的图示。

基于KEGG的结果目录: **10_Function/KEGG/cluster_barplot/**，文件格式同上。

4.39 基于功能的PCA分析

4.39.1 分析方法

在多元统计分析中，主成分分析PCA (Principal Component Analysis) 是一种简化数据集的技术。主成分分析经常用于减少数据集的维数，同时保持数据集中对方差贡献最大的特征，从而有效地找出数据中最“主要”的元素和结构，去除噪音和冗余，将原有的复杂数据降维，揭示隐藏在复杂数据背后的简单结构。

软件：**R**

4.39.2 结果说明

基于COG的结果目录: **10_Function/COG/PCA/**

COG_PCA_3D.pdf: 所有样本基于COG的PCA 3D图

COG_PCA_PCA*_VS_PCA*.pdf: 所有样本基于COG的PCA 2D图

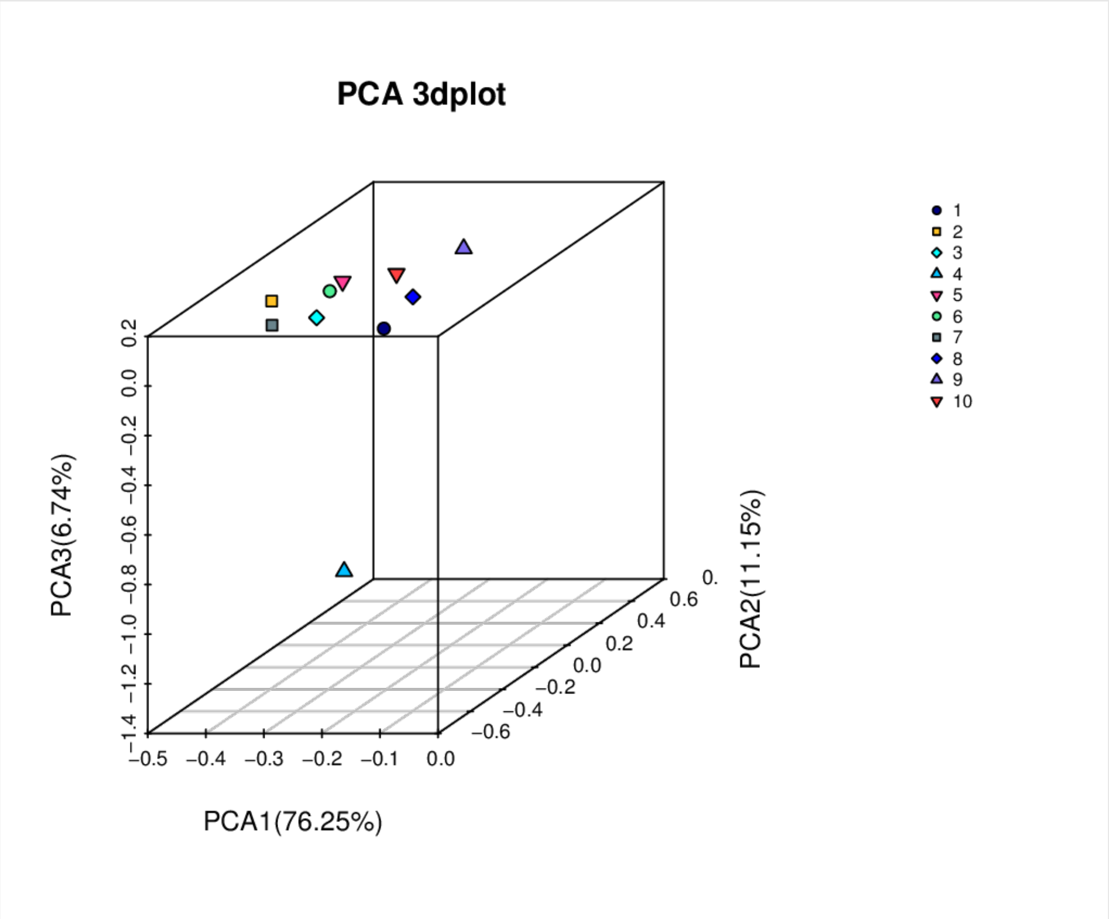

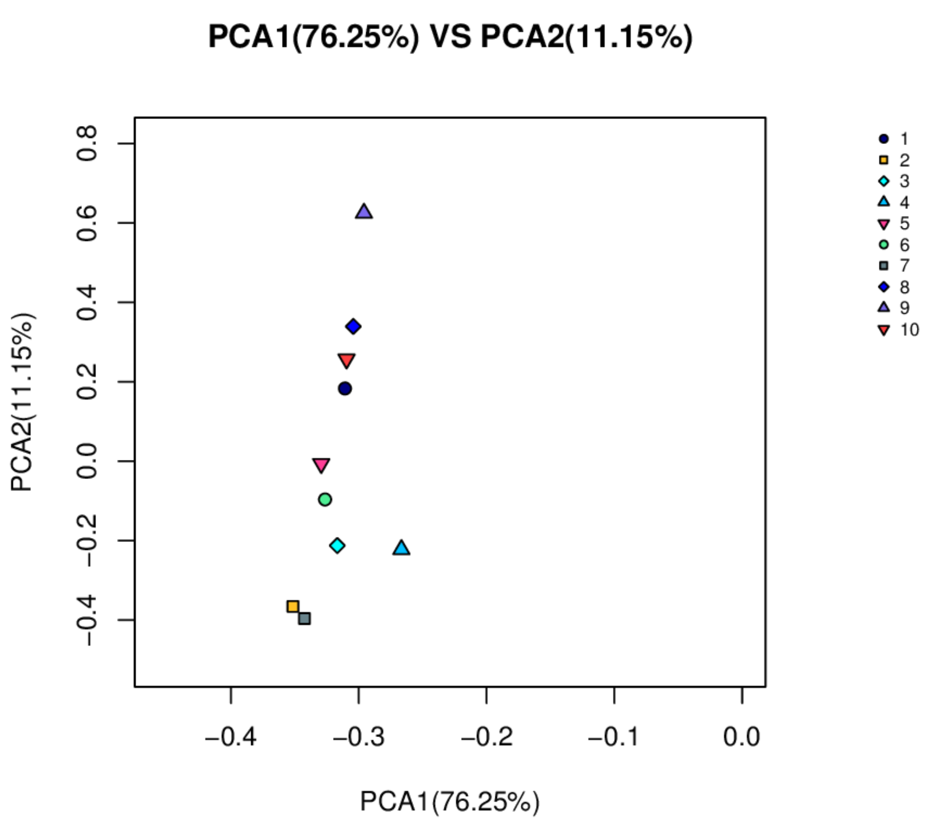

图36.1 基于COG的PCA图
图中不同颜色代表不同样本或者不同的group中的样本，样本间相似度越高则在图中越聚集。

基于KEGG的结果目录: **10_Function/KEGG/PCA/**，文件格式同上。

4.40 基于功能的NMDS分析

4.40.1 分析方法

非度量多维尺度法是一种将多维空间的研究对象 (样本或变量) 简化到低维空间进行定位、分析和归类，同时又保留对象间原始关系的数据分析方法。适用于无法获得研究对象间精确的相似性或相异性数据，仅能得到他们之间等级关系数据的情形。其基本特征是将对象间的相似性或相异性数据看成点间距离的单调函数，在保持原始数据次序关系的基础上，用新的相同次序的数据列替换原始数据进行度量型多维尺度分析。换句话说，当资料不适合直接进行变量型多维尺度分析时，对其进行变量变换，再采用变量型多维尺度分析，对原始资料而言，就称之为非度量型多维尺度分析。其特点是根据样品中包含的物种信息，以点的形式反映在多维空间上，而对不同样品间的差异程度，则是通过点与点间的距离体现的，最终获得样品的空间定位点图。

软件：**R**的**vegan** package

4.40.2 结果说明

基于COG的结果目录: **10_Function/COG/NMDS/**

COG_NMDS.pdf: 所有样本基于COG的NMDS图

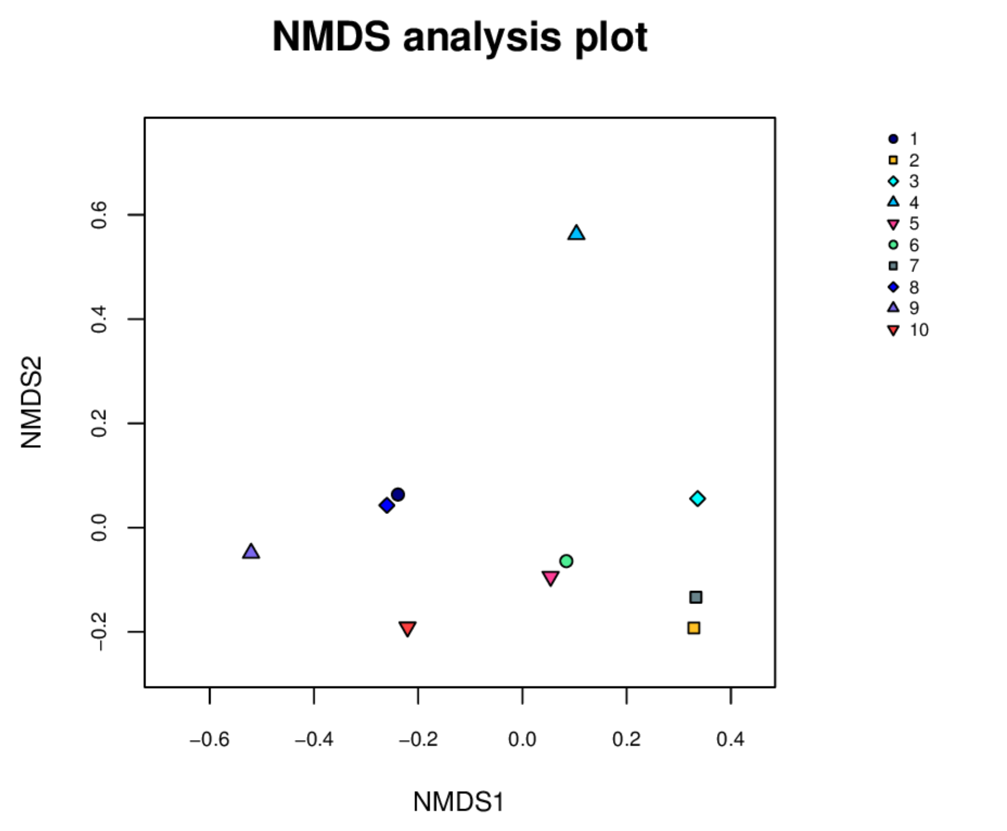

图40.1 基于COG的NMDS图
图中不同颜色代表不同样本或者不同的group中的样本，样本间相似度越高则在图中越聚集。

基于KEGG的结果目录: **10_Function/KEGG/NMDS/**，文件格式同上

4.41 Procrustes分析

4.41.1 分析方法

Procrustes test（强制一致性检验）是检验两个矩阵相关关系的非参数统计方法。这是 Mantel 检验的替代，但是使用简化的空间代替全部距离矩阵。

软件：**R**的**vegan** package

4.41.2 结果说明

基于COG的结果目录: **10_Function/COG/Procrustes/**

***_COG_*.pdf:** 物种丰度矩阵与COG丰度矩阵的Procrustes图

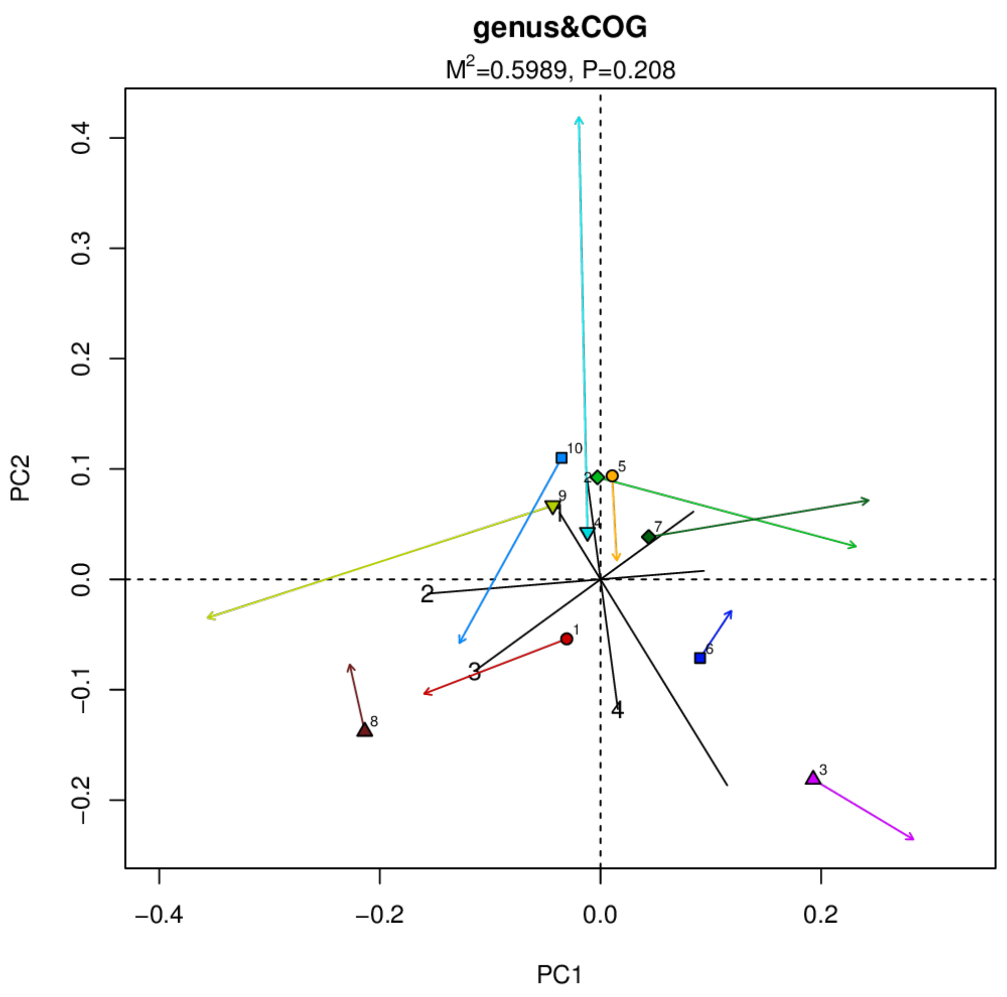

图41.1 属水平丰度矩阵与COG丰度矩阵的PCA Procrustes图
图中不同颜色代表不同样本或者不同的group中的样本，样本间相似度越高则在图中越聚集。箭头代表物种丰度到功能丰度之间的偏移量。

基于KEGG的结果目录: **10_Function/KEGG/Procrustes/**，文件格式同上

4.42 功能累计曲线图

4.42.1 分析方法

功能累积曲线图 (functional accumulation curves) 是用于描述随着样品量的加大功能增加的情况，是调查样本的功能组成和预测样品中功能丰度的有效工具，被广泛的用于样品量是否充分的判断以及功能丰富度

的估计。通过功能累积曲线不仅可以判断样品量是否充分，在样品量充分的前提下，运用功能累积曲线还可以对功能丰富度进行预测。

使用软件：**R**。

4.42.2 结果说明

基于COG的结果目录: **10_Function/COG/**

COG.accumulation.curves.pdf: COG功能累计曲线图

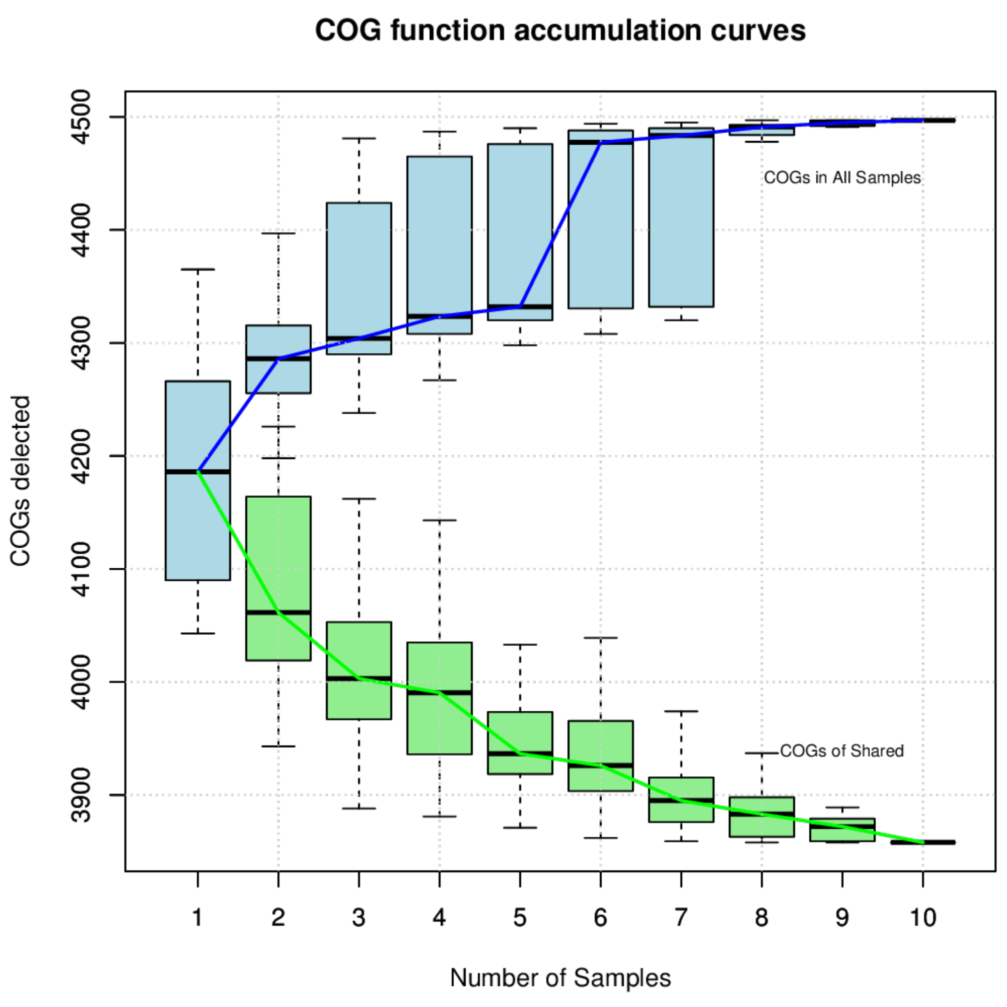

图42.1 COG功能累计曲线图
横坐标为样本数目，纵坐标为抽样后的COG ID数目。图中蓝色表示所有样本COG ID合集，绿色表示所有样本COG ID交集。结果反映了持续抽样下新COG 出现的速率。在一定范围内，随着样本量的加大，若曲线表现为急剧上升则表示群落中有大量新功能被发现；当曲线趋于平缓，则表示此环境中的功能并不会随样本量的增加而显著增多。利用功能累积曲线可以作为对样本量是否充分的判断，曲线急剧上升表明样本量不足，需要增加抽样量；反之，则表明抽样充分，可以进行数据分析。

基于KEGG的结果目录: **10_Function/KEGG/**，文件格式同上。

4.43 功能丰度差异分析

4.43.1 分析方法

基于PICRUSt功能二级分类结果，比较样本或组间丰度差异，找出样本或组间丰度存在显著差异的功能分类,默认筛选条件为P<=0.05。

当比较对象为样本时，采用fisher exact test；当比较对象为组时，采用Welch’s t-test。最后将检验得到的pvalue值采用FDR做Multiple test correction得到qvalue值。

软件：**STAMP**

4.43.2 结果说明

基于COG的结果目录: **10_Function/COG/Abu_Diff/**

diff_COG_reads.*.vs.*.xls: 两两差异比较文件

表43.1 两两差异比较结果

	Freq1	Freq2	pValue	qValue	Effect Size	95.0% lower CI	95.0% upper CI
[A] RNA processing and modification	0.02	5.8e-03	0.0	0.0	0.02	0.02	0.02
[W] Extracellular structures	0.01	1.1e-03	0.0	0.0	0.01	0.01	0.01
[V] Defense mechanisms	2.20	3.06	0.0	0.0	-0.87	-0.87	-0.86
[U] Intracellular trafficking, secretion, and vesicular transport	2.17	1.16	0.0	0.0	1.01	1.00	1.01
[T] Signal transduction mechanisms	5.01	6.19	0.0	0.0	-1.18	-1.19	-1.17
[S] Function unknown	7.87	7.04	0.0	0.0	0.83	0.82	0.84
[R] General function prediction only	10.93	11.19	0.0	0.0	-0.26	-0.27	-0.25
[Q] Secondary metabolites biosynthesis, transport, and catabolism	1.13	1.00	0.0	0.0	0.13	0.12	0.13

	Freq1	Freq2	pValue	qValue	Effect Size	95.0% lower CI	95.0% upper CI
[P] Inorganic ion transport and metabolism	4.59	4.07	0.0	0.0	0.51	0.51	0.52
[N] Cell motility	1.48	0.57	0.0	0.0	0.92	0.91	0.92
[L] Replication, recombination and repair	8.17	7.04	0.0	0.0	1.13	1.12	1.14
[M] Cell wall/ membrane/envelope biogenesis	5.50	5.88	0.0	0.0	-0.38	-0.39	-0.37
[J] Translation, ribosomal structure and biogenesis	4.92	5.96	0.0	0.0	-1.04	-1.05	-1.04
[I] Lipid transport and metabolism	2.07	2.17	0.0	0.0	-0.10	-0.11	-0.10

***/diff_COG_reads.*.vs.*.P0.05.xls:** P值检验显著的两两差异统计文件

***/diff_COG_reads.*.vs.*.ExtendErrorBar.pdf:** 差异比较的误差线图

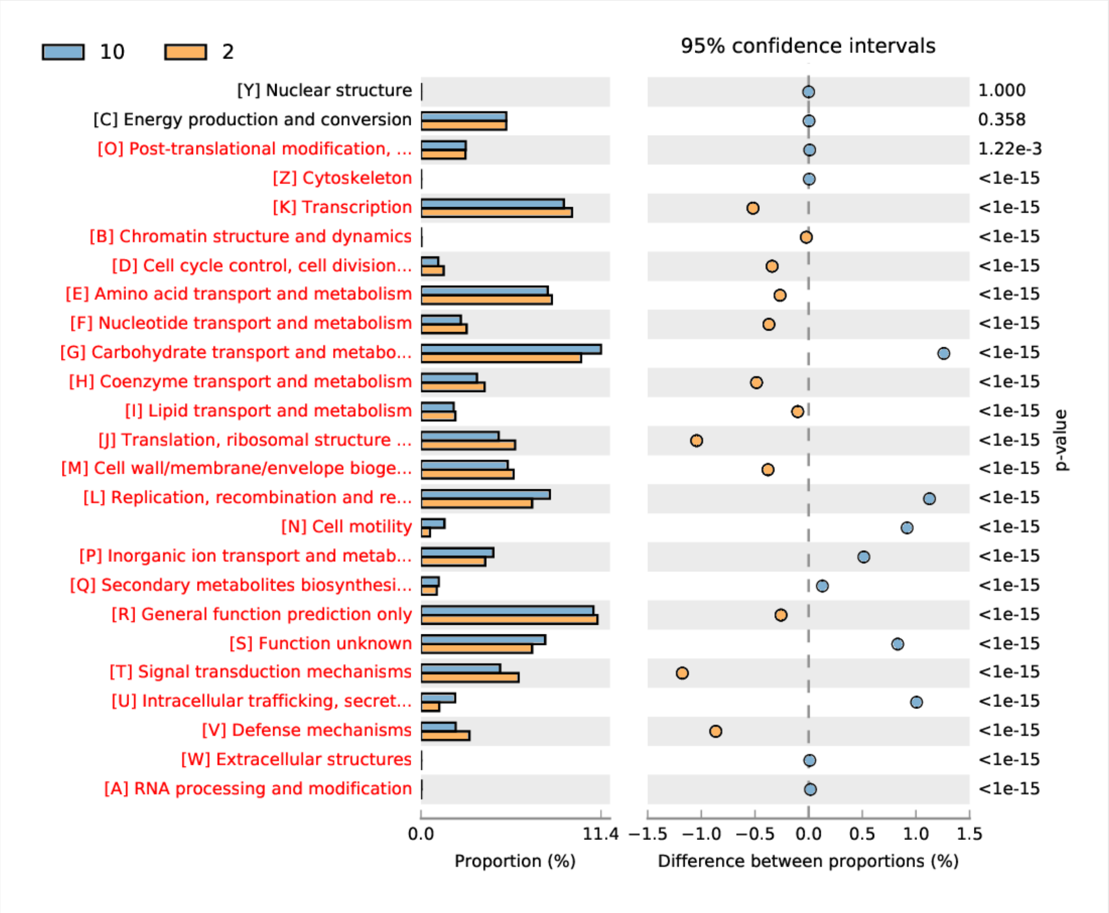

图43.2 差异比较的误差线图
左图所示为不同功能丰度在两个样品 (组) 中的丰度比例，中间所示为95%置信度区间内，功能丰度丰度的差异比例，最右边的值为p 值，p 值 < 0.05，表示差异显著，功能丰度用红色标识。(仅列出p值最低的25个)

基于KEGG的结果目录: **10_Function/KEGG/Abu_Diff/**，文件格式同上。

五. 分析结果文件说明

├── **1_data_for_analysis：数据文件**
│ ├── **1_Raw_data：原始测序数据**
│ ├── ├── *R1.fastq: 原始fastq测序数据文件
│ ├── **2_Merged_data：双端对拼后的数据**
│ ├── ├── merge.extendedFrags.fastq: 所有样本融合后的fastq文件
│ ├── ├── *.fastq: 各样本融合后的fastq文件
│ ├── ├── *_raw_read_len_distribution.pdf: 各分组的原始序列读长统计图
│ ├── **3_QC_data：QC之后的数据**
│ ├── ├── *.last.fastq: 各样本QC后的fastq文件
│ ├── ├── *_QC_read_len_distribution.pdf: 各分组的QC序列读长统计图
│ ├── Sample_infor.xls：质控之后统计统计文件
├── **2_filter_chimeras：去除嵌合体及非靶区域序列结果**
│ ├── *_no_chimeras.fasta：各样本去除嵌合体及非靶区域序列的fasta文件
│ ├── filter_chimeras_result.xls：去除嵌合体及非靶区域后统计表
├── **3_OTU：OTU分析结果**
│ ├──**VENN：OTU venn分析**
│ ├── ├── *_venn.pdf：样本OTU venn分析图
│ ├── ├── *_venn.xls：样本OTU venn分析表格
│ ├── **bray_crutis_tree：基于OTU丰度的样本聚类树分析文件夹**
│ ├── ├── *OTU_NMDS_bray_crutis_tree.pdf：基于Bray-Curtis算法，使用OTU信息的样本聚类图
│ ├── ├── *OTU_NMDS_bray_crutis_tree.phylo：基于Bray-Curtis算法，使用OTU信息的样本聚类结果
│ ├── ALL_OTU_count.xls：各样品OTU序列数统计结果
│ ├── ALL_OTU_count_add_taxonomy：OTU聚类结果与对应代表性序列统计结果
│ ├── gradient_plot.pdf：OTU聚类中OTU数目与similarity参数的关系图
├── **4_alpha_index：Alpha多样性分析结果**
│ ├── **diversity_box：多样性指数分析文件夹**
│ ├── ├── alpha_diversity_change_name.xls：指数统计表
│ ├── ├── Alpha_diversity*.pdf：样品分组之间的指数箱式图
│ ├── **Rank_abundance：Rank-abundance曲线分析文件夹**
│ ├── ├── rank_abundance.pdf：纵坐标是绝对值的rank_abundance图
│ ├── ├── rank_abundance_percentage.pdf：纵坐标是百分比的rank_abundance图
│ ├── **Rarefaction：稀释曲线分析**
│ ├── ├── *rarefaction_plot.pdf：稀释曲线图
│ ├── ├── *rarefraction_result_change_name.xls：稀释曲线分析表格
├── **5_Taxonomic_Classification：物种分类学分析结果**
│ ├── **barplot：样本群落结构分布柱状图**
│ ├── **bray_crutis_tree：基于物种丰度样本聚类树**

│ ├── cluster_barplot：样品聚类树与柱状图组合分析
│ ├── heatmap：物种丰度热图
│ ├── ├── boxplot：物种分类箱式图
│ ├── horiz_plot：物种丰度堆叠条形图
│ ├── Circos：样本与物种关系图
│ ├── Krona：单样品多级物种组成图
│ ├── GraPhlAn：分类和系统发育信息可视化
│ ├── iTOL：分类和系统发育信息可视化
│ ├── MEGAN：群落分类学系统树状图
│ ├── pie_plot：物种丰度饼图
│ ├── sample_barplot：物种分类丰度单样本柱状图
│ ├── unclassifier_plot：各水平未分类百分比图
│ ├── plot_raw_file：物种分类统计结果文件
├── 6_multi_dimension_analysis：多维分析结果
│ ├── OTU：基于OTU的多维分析结果
│ ├── ├── PCA：PCA分析
│ ├── ├── NMDS：NMDS分析
│ ├── ├── Network：网络图分析
│ ├── ├── Co-Network：微生物间相互关系网络图
│ ├── ├── corrplot：微生物间相互关系矩阵图
│ ├── Taxonomy：基于Taxonomy的多维分析结果 (结果与OTU类似)
├── 7_phylogenetic：系统发生进化树分析结果
│ ├── ALL_OTU_tree：所有OTU的聚类图
│ ├── ├── ALL_sample_tree_genus.pdf：所有OTU在genus水平上的聚类图
│ ├── ├── ALL_sample_tree_genus.circular.pdf：所有OTU在genus水平上的聚类环状图
│ ├── OTU_repre：前50个OTU的聚类图
│ ├── ├── first50_tree_genus.pdf：丰度最高的前50个OTU在genus水平上的聚类图
│ ├── ├── first50_tree_genus.circular.pdf：丰度最高的前50个OTU在genus水平上的聚类环状图
├── 8_unifrac：UniFrac分析
│ ├── unweighted：未加权的UniFrac分析
│ ├── ├── boxplot：Unifrac距离箱式图
│ ├── ├── heatmap：Unifrac距离热图
│ ├── ├── pcoa：PCoA分析
│ ├── ├── sample_tree：基于Unifrac距离的相似度树图
│ ├── ├── unweighted_unifrac_distance_matrix.xls：Unifrac距离矩阵表
│ ├── weighted：加权后的UniFrac分析 (结果与unweighted一致)
├── 9_Different：物种丰度差异分析结果
│ ├── Abu_Diff：样本间菌群丰度差异分析
│ ├── Ternaryplot：Ternary Plot图
├── 10_Function：PICRUSt功能分析
│ ├── COG：基于COG的功能分析
│ ├── ├── PICRUSt：功能预测结果

│ ├── ├── **barplot：所有样本功能结构分布柱状图**
│ ├── ├── **horiz_bar：单样本功能丰度条形图**
│ ├── ├── **bray_crutis_tree：基于功能丰度的样本聚类图**
│ ├── ├── **heatmap：功能丰度热图**
│ ├── ├── **cluster_barplot：样品聚类树与功能柱状图组合分析**
│ ├── ├── **PCA：基于功能的PCA分析**
│ ├── ├── **NMDS：基于功能的NMDS分析**
│ ├── ├── **Abu_Diff：功能丰度差异分析**
│ ├── ├── COG.accumulation.curves.pdf：功能累计曲线图
│ ├── **KEGG：基于KEGG的功能分析 (结果与COG一致)**

六. 参考文献

1. Claesson MJ, O'Sullivan O, Wang Q, et al. **Comparative Analysis of Pyrosequencing and a Phylogenetic Microarray for Exploring Microbial Community Structures in the Human Distal Intestine.** Ahmed N, ed. *PLoS ONE.* 2009;4(8):e6669. [PubMed]
2. Schmieder R, Edwards R. **Quality control and preprocessing of metagenomic datasets.** *Bioinformatics.* 2011;27(6):863-864. [PubMed]
3. Tanja Magoč, Steven L. Salzberg. **FLASH: Fast Length Adjustment of Short Reads to Improve Genome Assemblies.***Bioinformatics* 2011;27(21):2957-2963. [PubMed]
4. Zhang J, Kobert K, Flouri T, Stamatakis A. **PEAR: a fast and accurate Illumina Paired-End reAd mergeR.** *Bioinformatics.* 2014;30(5):614-620. [PubMed]
5. Schloss PD, Westcott SL, Ryabin T, et al. **Introducing mothur: Open-Source, Platform-Independent, Community-Supported Software for Describing and Comparing Microbial Communities .** *Applied and Environmental Microbiology.* 2009;75(23):7537-7541. [PubMed]
6. Edgar RC. **Search and clustering orders of magnitude faster than BLAST.** *Bioinformatics.* 2010;26(19):2460-2461. [PubMed]
7. Caporaso JG, Kuczynski J, Stombaugh J, et al. **QIIME allows analysis of high-throughput community sequencing data.** *Nature methods.* 2010;7(5):335-336. [PubMed]
8. Edgar RC. **MUSCLE: multiple sequence alignment with high accuracy and high throughput.** *Nucleic Acids Research.* 2004;32(5):1792-1797. [PubMed]
9. Huson DH, Mitra S, Ruscheweyh H-J, Weber N, Schuster SC. **Integrative analysis of environmental sequences using MEGAN4.** *Genome Research.* 2011;21(9):1552-1560. [PubMed]
10. Wang Q, Garrity GM, Tiedje JM, Cole JR. **Naïve Bayesian Classifier for Rapid Assignment of rRNA Sequences into the New Bacterial Taxonomy .** *Applied and Environmental Microbiology.* 2007;73(16):5261-5267. [PubMed]
11. Altschul SF, Madden TL, Schäffer AA, et al. **Gapped BLAST and PSI-BLAST: a new generation of protein database search programs.** *Nucleic Acids Research.* 1997;25(17):3389-3402. [PubMed]
12. Caporaso JG, Bittinger K, Bushman FD, DeSantis TZ, Andersen GL, Knight R. **PyNAST: a flexible tool for aligning sequences to a template alignment.** *Bioinformatics.* 2010;26(2):266-267. [PubMed]
13. Edgar RC, Haas BJ, Clemente JC, Quince C, Knight R. **UCHIME improves sensitivity and speed of chimera detection.** *Bioinformatics.* 2011;27(16):2194-2200. [PubMed]
14. Price MN, Dehal PS, Arkin AP. **FastTree 2 – Approximately Maximum-Likelihood Trees for Large Alignments.** Poon AFY, ed. *PLoS ONE.* 2010;5(3):e9490. [PubMed]

15. Parks DH, Tyson GW, Hugenholtz P, Beiko RG. **STAMP: statistical analysis of taxonomic and functional profiles.** *Bioinformatics.* 2014;30(21):3123-3124. [PubMed]
16. Langille MGI, Zaneveld J, Caporaso JG, et al. **Predictive functional profiling of microbial communities using 16S rRNA marker gene sequences.** *Nature biotechnology.* 2013;31(9):814-821. [PubMed]
17. Asnicar F, Weingart G, Tickle TL, Huttenhower C, Segata N. **Compact graphical representation of phylogenetic data and metadata with GraPhlAn.** *PeerJ.* 2015;3:e1029. [PubMed]
18. Segata N, Izard J, Waldron L, et al. **Metagenomic biomarker discovery and explanation.** *Genome Biology.* 2011;12(6):R60. [PubMed]
19. Ondov BD, Bergman NH, Phillippy AM. **Interactive metagenomic visualization in a Web browser.** *BMC Bioinformatics.* 2011;12(1):385. [PubMed]
20. Letunic I and Bork P. **Interactive tree of life (iTOL) v3: an online tool for the display and annotation of phylogenetic and other trees.** *Nucleic Acids Res.* 2016;pii: gkw290. [PubMed]
21. Quast C, Pruesse E, Yilmaz P, et al. **The SILVA ribosomal RNA gene database project: improved data processing and web-based tools.** *Nucleic Acids Research.* 2013;41(Database issue):D590-D596. [PubMed]
22. Urmas Kõljalg, R. Henrik Nilsson, Kessy Abarenkov, Leho Tedersoo, et al. **Towards a unified paradigm for sequence-based identification of fungi.** *Molecular Ecology* 2013;22,5271–5277. [PubMed]
