# Supplementary material for: Enteral nutrition modulation with n-3 PUFAs directs microbiome and lipid metabolism in mice
Source: PLoS One. 2021 Mar 25;16(3):e0248482. doi: 10.1371/journal.pone.0248482 (PMC7993877; doi:10.1371/journal.pone.0248482)
Supplement: S2 File — (PDF) [file pone.0248482.s003.pdf]

Tel: 0571-56892193
杭州市西湖区黄姑山路9号天科大厦

# 天科16S分析报告

| | |
|---|---|
| 项目编号 | TK190412H0106 |
| 分析类别 | 细菌16S扩增子测序分析 |
| 样品来源 | 环境 |
| 测序平台 | Miseq |
| 报告单位 | 浙江天科高新技术发展有限公司 |
| 报告日期 | 2019.05.07 |

# 1 建库测序

16S rRNA位于原核细胞核糖体小亚基上，包括 10 个保守区域（Conserved Regions）和 9 个高变区域（Hypervariable Regions），其中保守区在细菌间差异不大，高变区具有属或种的特异性，随亲缘关系不同而有一定的差异。因此，16S rDNA可以做作为揭示生物物种的特征核酸序列，被认为是最适于细菌系统发育和分类鉴定的指标。16S rDNA扩增子测序（16S rDNA Amplicon Sequencing），通常是选择某个或某几个变异区域，利用保守区设计通用引物进行PCR扩增，然后对高变区进行测序分析和菌种鉴定，16S rDNA扩增子测序技术已成为研究环境样品中微生物群落组成结构的重要手段。

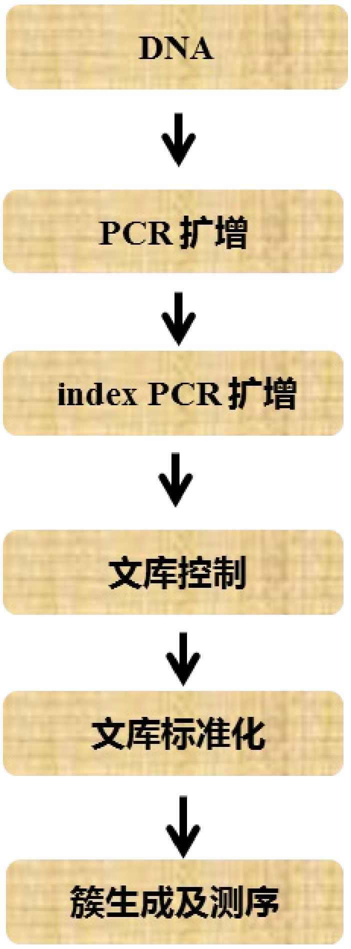


图1.1 16S rDNA建库测序流程图

图1.1 描述的是16S rDNA测序实验步骤:采用 PowerSoil® DNA Isolation Kit(MO BIO, Cat.No.12888) 方法对样本的基因组 DNA 进行提取，之后利用琼脂糖凝胶电泳检测DNA的纯度和浓度，取适量的基因组 DNA 为模板，根据测序区域的选择，使用带 Barcode 的特异引物，New England Biolabs 公司的 Phusion® High-Fidelity PCR Master Mix with GC Buffer，和高效高保真酶进行PCR，确保扩增效率和准确性提取DNA样品、样品检测、PCR、纯化、建库、使用Illumina测序仪进行测序。

## 2 分析流程

测序得到的原始数据（Raw Data），存在一定比例的干扰数据（Dirty Data），为了使信息分析的结果更加准确、可靠，首先对原始数据进行拼接、过滤，得到有效数据（Clean Data）。然后基于有效数据进行OTUs（Operational Taxonomic Units ）聚类和物种分类分析。根据OTUs聚类结果，一方面对每个OTU的代表序列做物种注释，得到对应的物种信息和基于物种的丰度分布情况。同时，对OTUs进行丰度、Alpha多样性计算、Venn图和花瓣图等分析，以得到样品内物种丰富度和均匀度信息、不同样品或分组间的共有和特有OTUs信息等。另一方面，可以对OTUs进行多序列比对并构建系统发生树，并进一步得到不同样品和分组的群落结构差异，通过PCoA和PCA、NMDS等降维图和样品聚类树进行展示。为进一步挖掘分组样品间的群落结构差异，选用T-test、MetaStat、LEfSe、Anosim和MRPP等统计分析方法对分组样品的物种组成和群落结果进行差异显著性检验。 同时，也可结合环境因素进行CCA/RDA分析和多样性指数与环境因子的相关性分析，得到显著影响组间群落变化的环境影响因子。获得下机数据后的信息分析流程如下图：

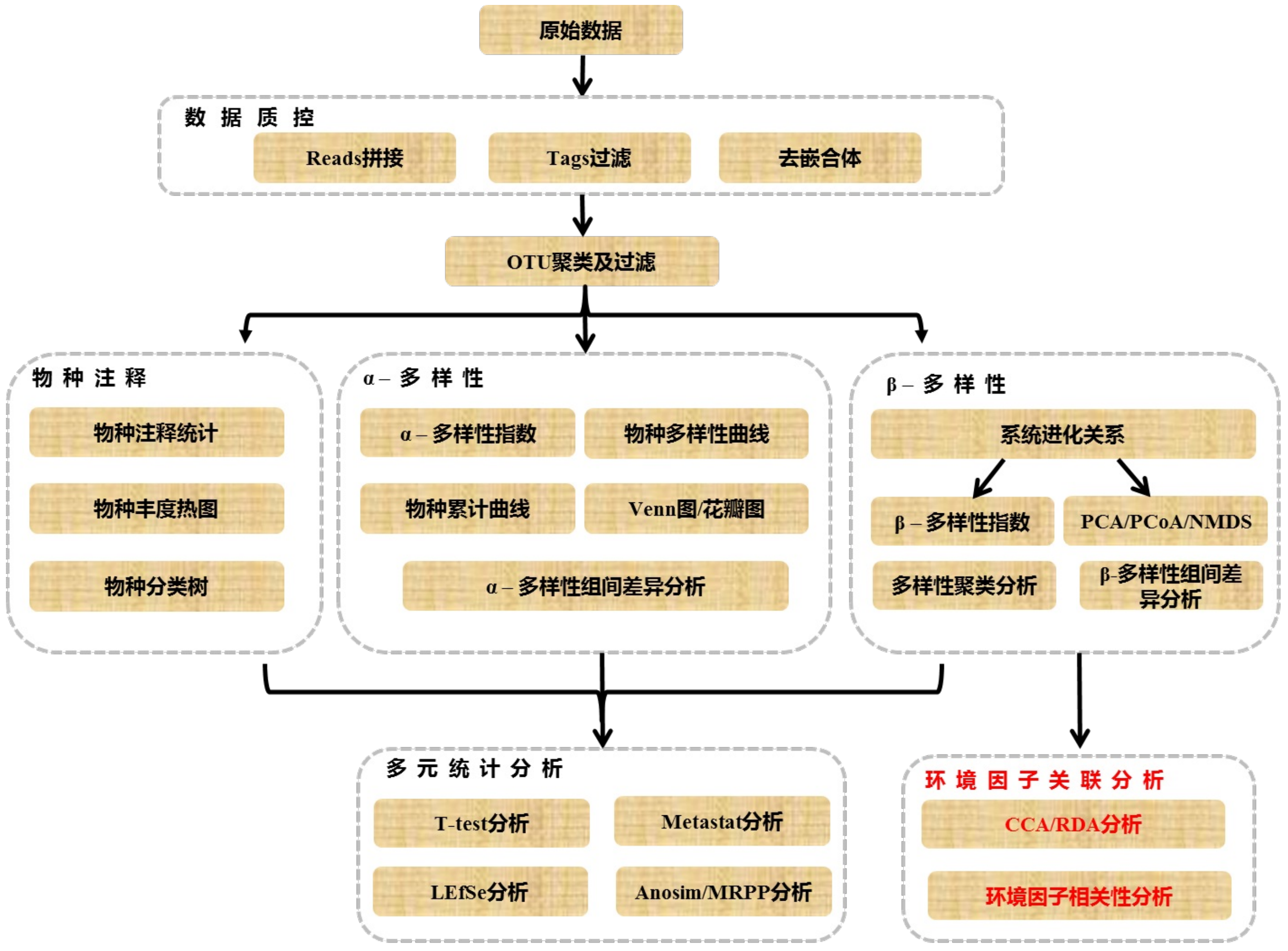


图2.1 16S rDNA标准信息分析流程图

# 3 分析结果

## 3.1 测序数据处理

### 3.1.1 原始测序数据

二代高通量测序仪器（如Illumina HiSeq$^{TM}$ 2500）测序得到的原始图像数据经base calling转化为序列数据，我们称之为raw data或raw reads，结果以FASTQ文件格式存储，包含reads的序列以及碱基的测序质量。在FASTQ格式文件中每个read由四行描述，如下：

```
@HWI-7001457:334:C95HLANXX:7:1101:9740:5060 1:N:0:ATCACG
ATCACATAAAAATGGTCTGAAAAGGCGAATGATCAGAGATGAAAATGATACTCATTTTGATCATCCTTCGCCAACCTATGATCTGAAAG
+
/BBBBFFFFFFFFFFFFFFFFFFFFFFFFFFFFFFFBFFFFFFFFFFBFBBFFFFFFFFFFFFFFFFFFFFFFBFBFFFFFFFFFFFFFBFFFFFF
```

*ReadMe*

每个序列共有4行，第1行和第3行是序列名称（有的fq文件为了节省存储空间会省略第三行“+”后面的序列名称），由测序仪产生；第2行是序列；第4行是序列的测序质量，每个字符对应第2行每个碱基，第四行每个字符对应的ASCII值减去64(或者33)，即为该碱基的测序质量值。测序下机的原始数据的信息统计如下:

**表3.1 原始下机数据统计表**

| #Sample_name | Total_reads | Combined_reads | Uncombined_reads | Percent_combined(%) | Combined_base(bp) | Min_len(bp) | Max_len(bp) | Avg_len(bp) |
|---|---|---|---|---|---|---|---|---|
| C1 | 53,004 | 51,350 | 1,654 | 96.88 | 21,326,841 | 399 | 474 | 415 |
| C2 | 54,577 | 52,721 | 1,856 | 96.60 | 21,809,360 | 399 | 478 | 414 |
| C3 | 53,720 | 52,035 | 1,685 | 96.86 | 21,796,488 | 399 | 481 | 419 |
| CHB10 | 53,197 | 51,396 | 1,801 | 96.61 | 21,537,055 | 308 | 477 | 419 |
| CHB13 | 54,510 | 53,121 | 1,389 | 97.45 | 22,317,900 | 399 | 475 | 420 |
| H10 | 53,702 | 51,889 | 1,813 | 96.62 | 21,780,467 | 400 | 475 | 420 |
| H8 | 55,151 | 53,486 | 1,665 | 96.98 | 22,112,838 | 399 | 476 | 413 |
| IT20 | 51,995 | 50,468 | 1,527 | 97.06 | 21,180,633 | 372 | 476 | 420 |
| IT22 | 51,025 | 49,112 | 1,913 | 96.25 | 20,653,178 | 400 | 475 | 421 |
| #Total | 480,881 | 465,578 | 15,303 | 96.81 | 194,514,760 | 308 | 481 | 418 |

*ReadMe*

结果目录见：00.RawData

### 3.1.2 Clean data

测序得到的raw data，含有接头和barcode序列，信息分析前需要去除这些序列；然后使用**pear**软件将有overlap的reads进行拼接，使用**QIIME**对拼接数据进行过滤，过滤掉含N较多或者低质量序列，最后进行嵌合体过滤，得到可用于后续分析的有效数据，即Effective Tags，统计信息如下：

过滤得到的数据统计信息见下表：

**表3.2 Clean tag 数据统计表**

| #Sample_name | Raw_PE | Combined | Qualified | Nochime | Base(nt) | AvgLen(nt) | Q20 | Q30 | GC% | Effective% |
|---|---|---|---|---|---|---|---|---|---|---|
| C1 | 53,004 | 51,350 | 48,130 | 45,253 | 18,689,632 | 413 | 98.78 | 95.59 | 49.51 | 85.38 |
| C2 | 54,577 | 52,721 | 49,149 | 46,780 | 19,243,978 | 411 | 98.72 | 95.44 | 50.71 | 85.71 |
| C3 | 53,720 | 52,035 | 47,889 | 39,754 | 16,575,996 | 417 | 98.71 | 95.39 | 49.38 | 74.00 |
| CHB10 | 53,197 | 51,396 | 47,534 | 41,254 | 17,217,739 | 417 | 98.71 | 95.36 | 48.11 | 77.55 |
| CHB13 | 54,510 | 53,121 | 48,825 | 43,490 | 18,194,735 | 418 | 98.74 | 95.48 | 49.09 | 79.78 |

| | | | | | | | | | | |
|---|---|---|---|---|---|---|---|---|---|---|
| H10 | 53,702 | 51,889 | 48,004 | 42,243 | 17,653,488 | 418 | 98.73 | 95.46 | 48.18 | 78.66 |
| H8 | 55,151 | 53,486 | 50,326 | 48,844 | 20,078,932 | 411 | 98.77 | 95.59 | 48.98 | 88.56 |
| IT20 | 51,995 | 50,468 | 46,920 | 46,848 | 19,555,218 | 417 | 98.71 | 95.38 | 49.68 | 90.10 |
| IT22 | 51,025 | 49,112 | 45,418 | 36,312 | 15,203,159 | 419 | 98.80 | 95.68 | 48.42 | 71.17 |

*ReadMe*

结果目录见：01.CleanData/

## 3.2 OTU分析和物种注释

### 3.2.1 OTU聚类及物种注释概况

为了研究样品的物种组成多样性，使用**uparse(http://www.drive5.com/uparse/)**软件对所有样品的Effective Tags进行聚类，以97%的一致性（Identity）将序列聚类成为OTUs（Operational Taxonomic Units），然后对OTUs的代表序列进行物种注释。

为了方便快速全面的了解各样品的OTU聚类情况和注释情况，对各样品的OTU聚类和注释结果进行了综合统计，结果如下:

**表3.3 Tags及OTUs数目统计表**

| Sample | Total_tags | Non_pollution_tags | Taxon_tags | OTUs |
|---|---|---|---|---|
| C1 | 45,253 | 45,253 | 43,926 | 131 |
| C2 | 46,780 | 46,780 | 42,922 | 186 |
| C3 | 39,754 | 39,754 | 37,510 | 130 |
| CHB10 | 41,254 | 41,253 | 39,827 | 123 |
| CHB13 | 43,490 | 43,490 | 42,238 | 158 |
| H10 | 42,243 | 42,243 | 40,802 | 113 |
| H8 | 48,844 | 48,844 | 48,217 | 89 |
| IT20 | 46,848 | 46,841 | 45,580 | 137 |
| IT22 | 36,312 | 36,312 | 34,538 | 102 |

ReadMe

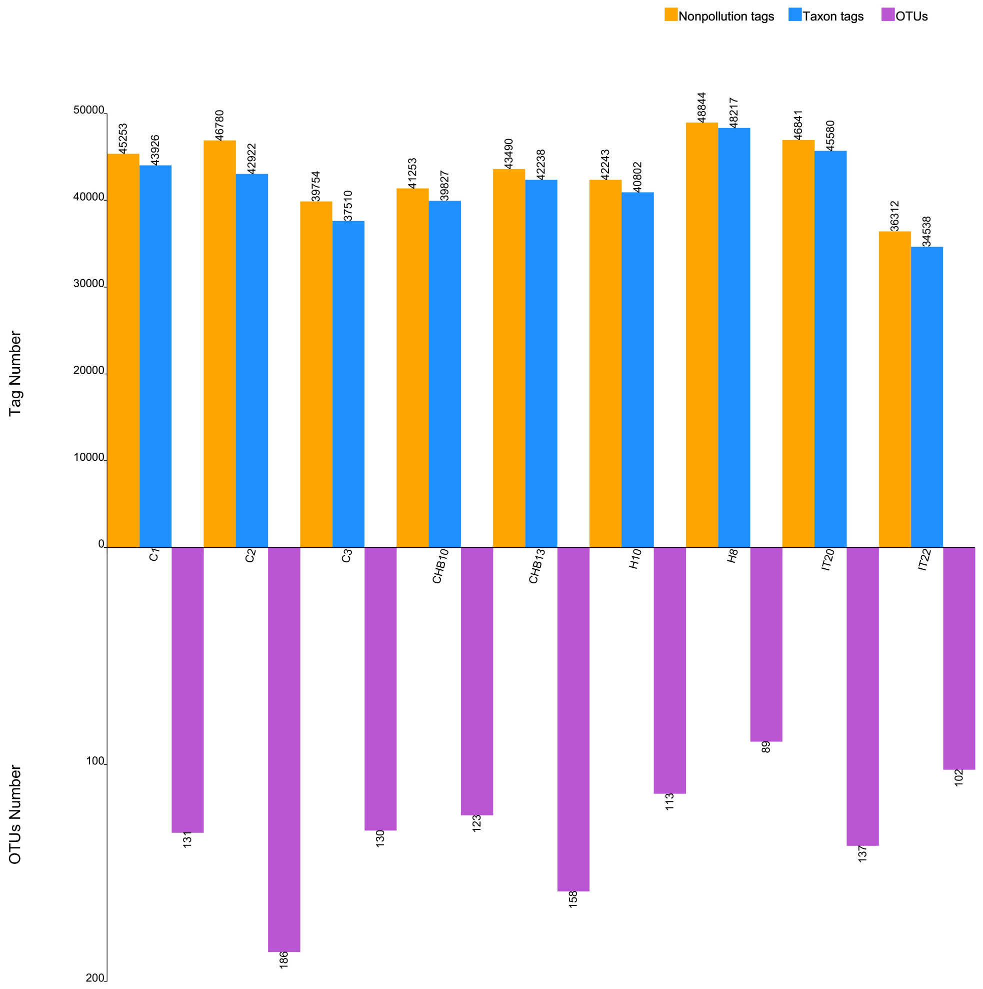


**图3.1 各样品的OTUs聚类和注释情况统计**

横坐标是样品名；上方纵坐标(Tags Number)是Tags数目；Total Tags/Nonpollution_tags（橙色）：指每个样品用于OTU聚类的Tags数目（等同于Effective Tags）；Taxon Tags（蓝色）：指用于构建OTUs并且获得注释信息的Tags数目；Singletons（绿色）：指频数为1，并且无法被聚类到OTUs的Tags数目（无法聚类到OTUs的序列将不用于后续分析）；Unclassified Tags（粉色）指没有获得注释信息的Tags数目；下方纵坐标(OTUs Number)是OTUs的数目，OTUs（紫色）：指每个样品得到的OTUs数目。

另外，注释使用的数据库默认为silva数据库，根据物种注释情况，统计每个样品注释到各分类水平(界Kingdom{k}、门Phylum{p}、纲Class{c}、目Order{o}、科Family{f}、属Genus{g}、种Species{s})上的序列数目，由此可以了解各分类水平的整体注释情况，结果如下：

**表3.4 各分类水平的Tags数目分布统计表**

| Classification | k | p | c | o | f | g | s |
|---|---|---|---|---|---|---|---|
| C1 | 43,926 | 43,925 | 43,925 | 43,925 | 43,922 | 43,790 | 10,968 |
| C2 | 42,922 | 42,921 | 42,921 | 42,921 | 42,919 | 42,564 | 13,107 |
| C3 | 37,510 | 37,510 | 37,510 | 37,510 | 37,509 | 37,209 | 7,848 |
| CHB10 | 39,827 | 39,826 | 39,826 | 39,826 | 39,633 | 39,435 | 7,699 |
| CHB13 | 42,238 | 42,237 | 42,237 | 42,237 | 42,123 | 41,980 | 4,498 |
| H10 | 40,802 | 40,802 | 40,802 | 40,802 | 40,798 | 40,752 | 10,490 |
| H8 | 48,217 | 48,215 | 48,215 | 48,215 | 48,214 | 48,126 | 2,021 |
| IT20 | 45,580 | 45,552 | 45,552 | 45,552 | 45,490 | 45,362 | 12,497 |
| IT22 | 34,538 | 34,538 | 34,538 | 34,538 | 34,537 | 34,504 | 10,880 |

*ReadMe*

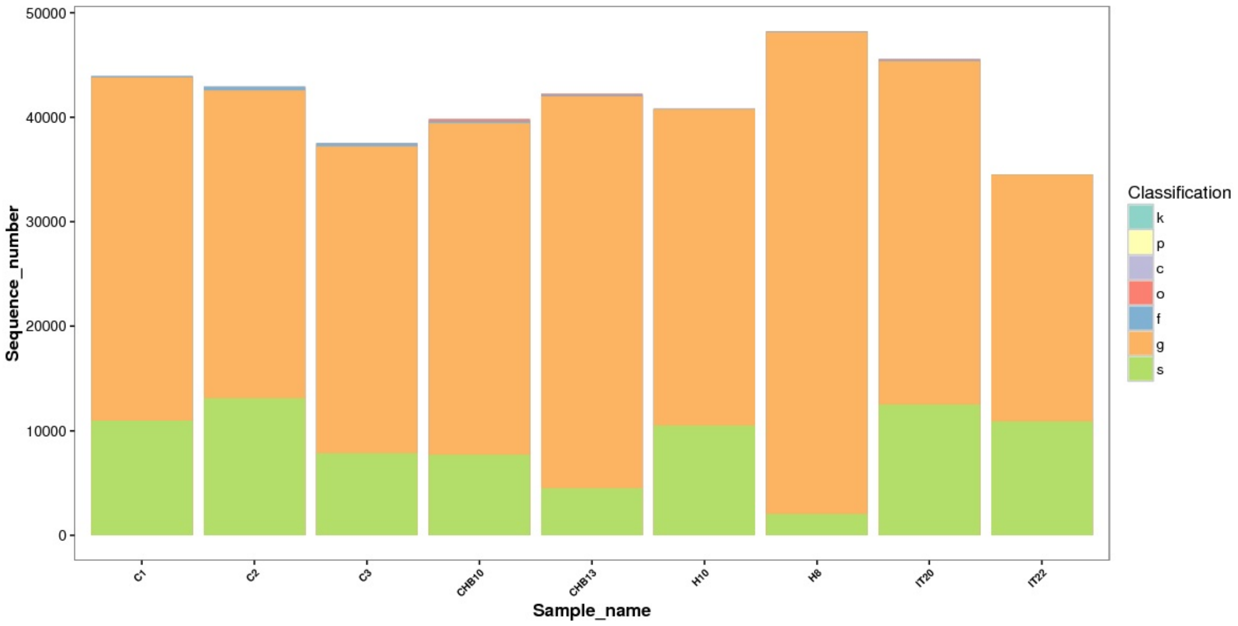


**图3.2 各样品在各分类水平上的序列数目**

各样品的Tags及OTUs数目统计分布结果见：
./02.OTUanalysis/taxa_stat/Sample_Tag_OTU_stat.xls;./02.OTUanalysis/taxa_stat/Sample_Tag_OTU_stat.{svg,png}
各样品在各分类水平上的序列数目见： ./02.OTUanalysis/taxa_stat/kpcofgs_Tag_stat_classify.xls;
./02.OTUanalysis/taxa_stat/AllSamples.kpcofgs_Tag_stat_classify.png.{pdf,png}

### 3.2.2 物种分布情况

**物种相对丰度展示**

根据物种注释结果，统计每个样品在各分类水平（Phylum、 Class、 Order、 Family、 Genus）上的均一化之前的绝对丰度、均一化之后的绝对丰度，均一化之后的相对丰度，结果见：02.OTUanalysis/taxa_abundance/；以均一化之后门水平的相对丰度为例，结果形式展示如下表：

**表3.5 相对丰度示例表**

| Taxonomy | C1 | C2 | C3 | ... | SampleN | Tax_detail |
|---|---|---|---|---|---|---|
| Bacteroidetes | 0.537350 | 0.454745 | 0.700590 | ... | ... | k__Bacteria;p__Bacteroidetes; |
| Firmicutes | 0.444032 | 0.516851 | 0.280155 | ... | ... | k__Bacteria;p__Firmicutes; |
| Fusobacteria | 0 | 0.000839 | 0 | ... | ... | k__Bacteria;p__Fusobacteria; |
| Proteobacteria | 0.016851 | 0.006253 | 0.016764 | ... | ... | k__Bacteria;p__Proteobacteria; |
| Actinobacteria | 0.001563 | 0.021078 | 0.002490 | ... | ... | k__Bacteria;p__Actinobacteria; |

根据物种注释结果，选取每个样品在各分类水平（Phylum、 Class、 Order、 Family、 Genus）上最大丰度排名前10的物种，生成物种相对丰度柱形累加图，以便直观查看各样品在不同分类水平上，相对丰度较高的物种及其比例。以门水平物种相对丰度柱形图为例展示如下

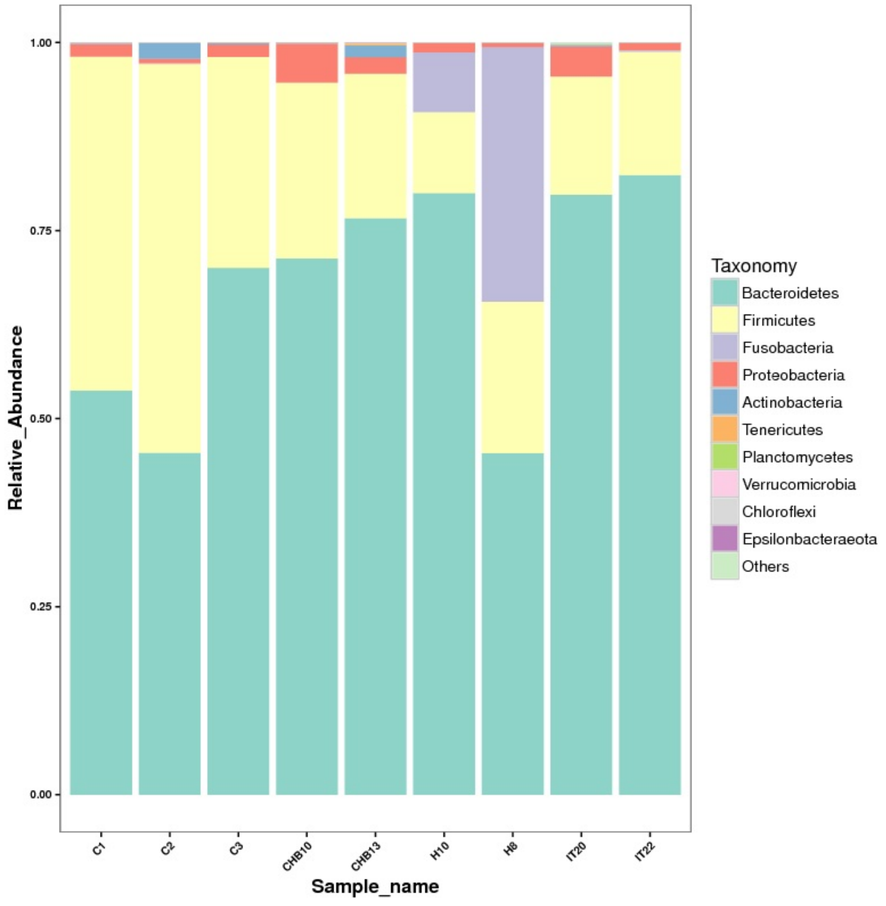


**图3.3 门水平上的物种相对丰度柱形图**

横坐标（Sample Name）是样品名；纵坐标（Relative Abundance）表示相对丰度；Others表示图中这10个门之外的其他所有门的相对丰度之和。

top10物种相对丰度柱形图见：02.OTUanalysis/top10/;包括门纲目科属（Phylum、 Class、 Order、 Family、 Genus）5个分类级别的结果。

**物种丰度聚类热图**

根据所有样品在属水平的物种注释及丰度信息，选取丰度排名前35的属，根据其在每个样品中的丰度信息，从物种和样品两个层面进行聚类，绘制成热图，便于发现哪些物种在哪些样品中聚集较多或含量较低。结果展示见：

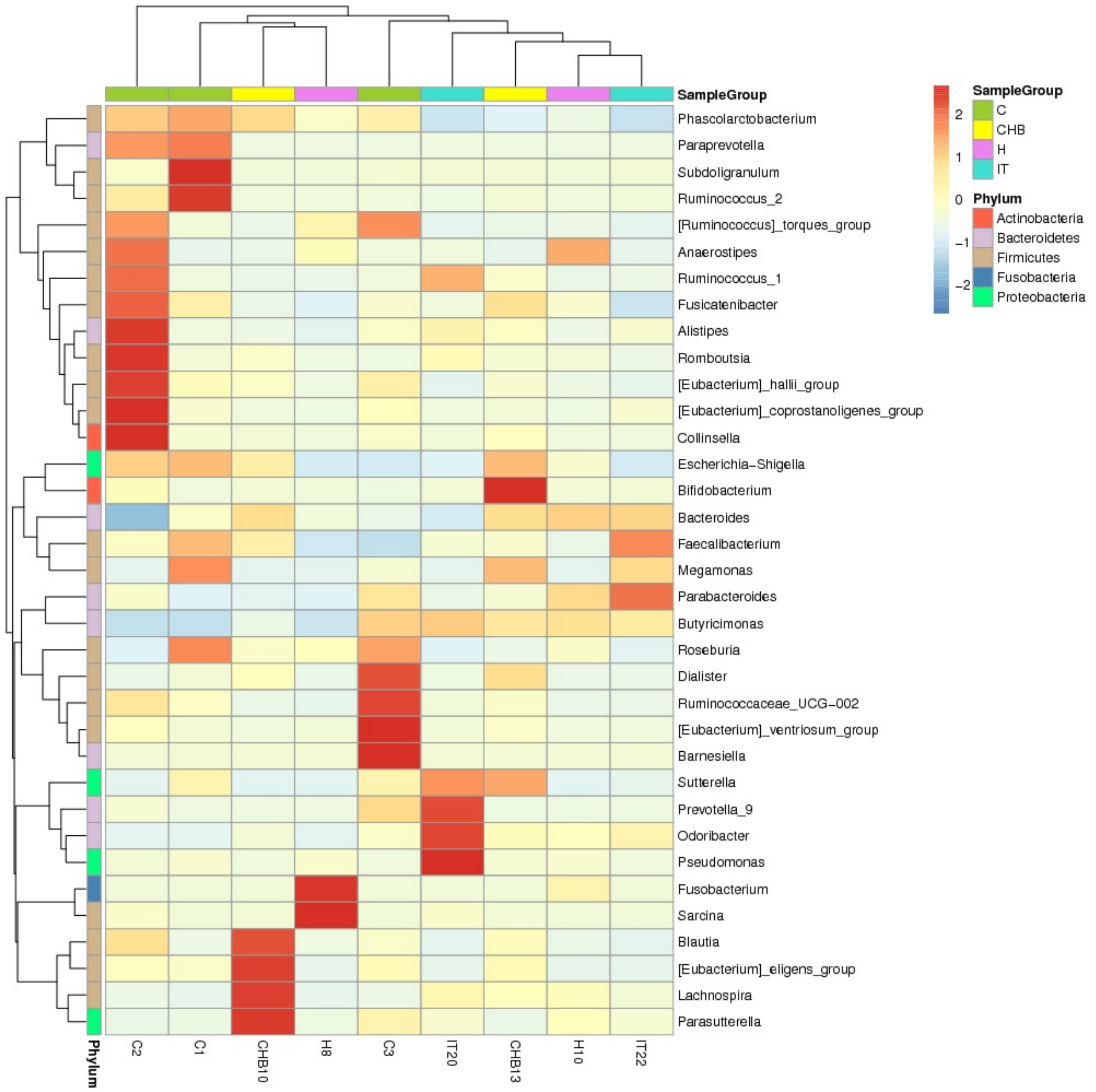


**图3.4 物种丰度聚类图**

纵向为样品信息，横向为物种注释信息，图中左侧的聚类树为物种聚类树；上方的聚类树为样品组间的聚类树；中间热图对应的值为每一行物种相对丰度经过标准化处理后得到的Z值，即一个样品在某个分类上的Z值为样品在该分类上的相对丰度和所有样品在该分类的平均相对丰度的差除以所有样品在该分类上的标准差所得到的值。

结果目录见：02.OTUanalysis/taxa_heatmap/cluster.taxa.png

### 3.2.3 物种分类树分析

**特定物种分类树**

对每个样品的物种分类结果，筛选特别关注的物种（默认选择最大相对丰度前10的属）进行物种分类树统计并进行画图展示，其中单个样品的物种分类树见下第一张图（点击图片显示高清大图）。多个样品的物种分类树见下第二张图。

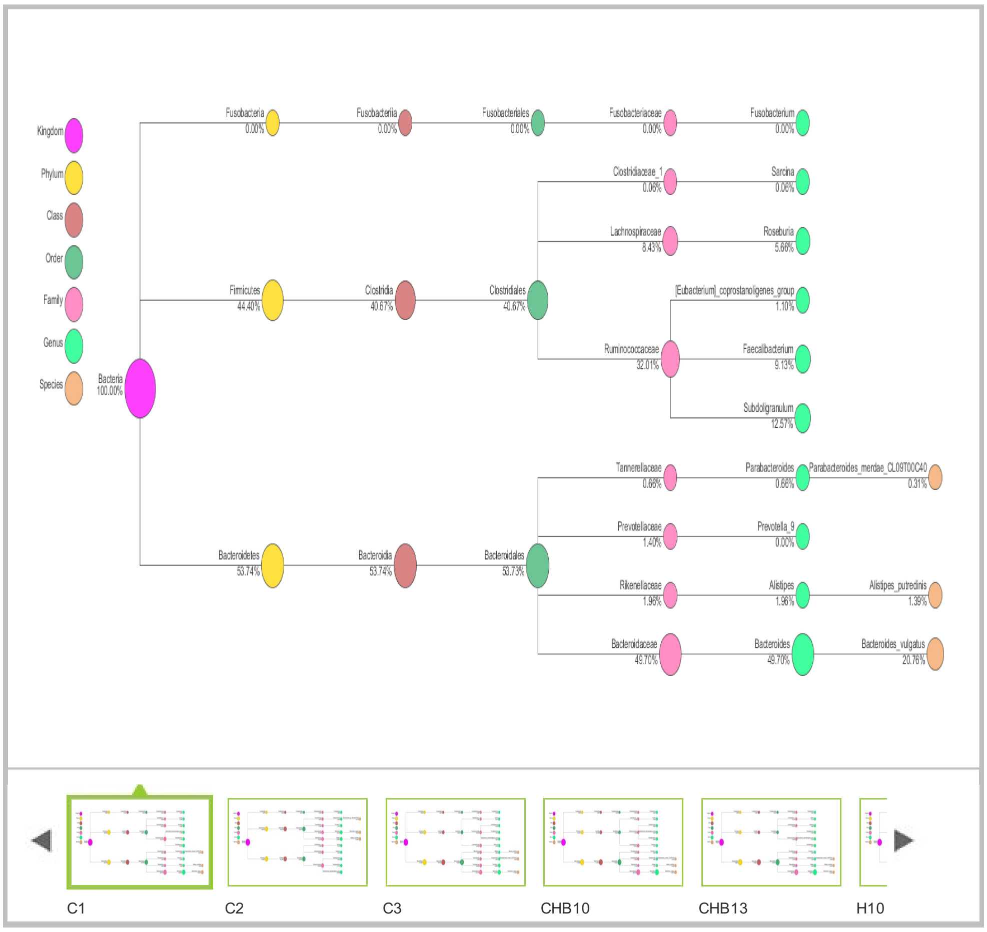


**图3.5 单样品中特定物种分类树**

不同颜色的圆圈表示不同的分类水平，对应左侧图例；圆圈的大小代表该分类的相对丰度大小；分类名下方的数字表示相对丰度百分率，即该分类占该样品中所有分类物种的百分率。

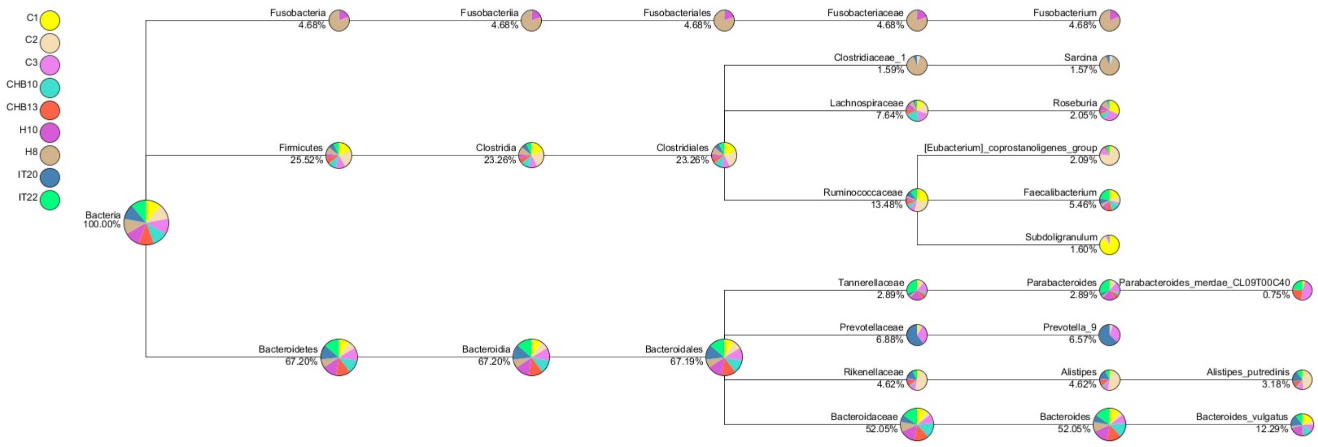


**图3.6 多样品中特定物种分类树**

圆圈中不同颜色的扇形表示不同的样品，对应左侧图例；扇形的大小表示该样品在该分类上相对丰度的比例大小；分类名下方的数字表示所有样品在该分类上

的平均相对丰度百分率。

ReadMe

结果目录见：./02.OTUanalysis/taxa_tree

组间结果目录见./02.OTUanalysis/taxa_tree_group

## 物种注释结果KRONA展示

使用**KRONA**软件对物种注释结果进行可视化展示，展示结果中见文件**result_krona.html**，圆圈从内到外依次代表不同的分类级别，扇形的大小代表不同OTU注释结果的相对比例，更多详细的信息请参考KRONA展示结果详解。示例图如下所示:

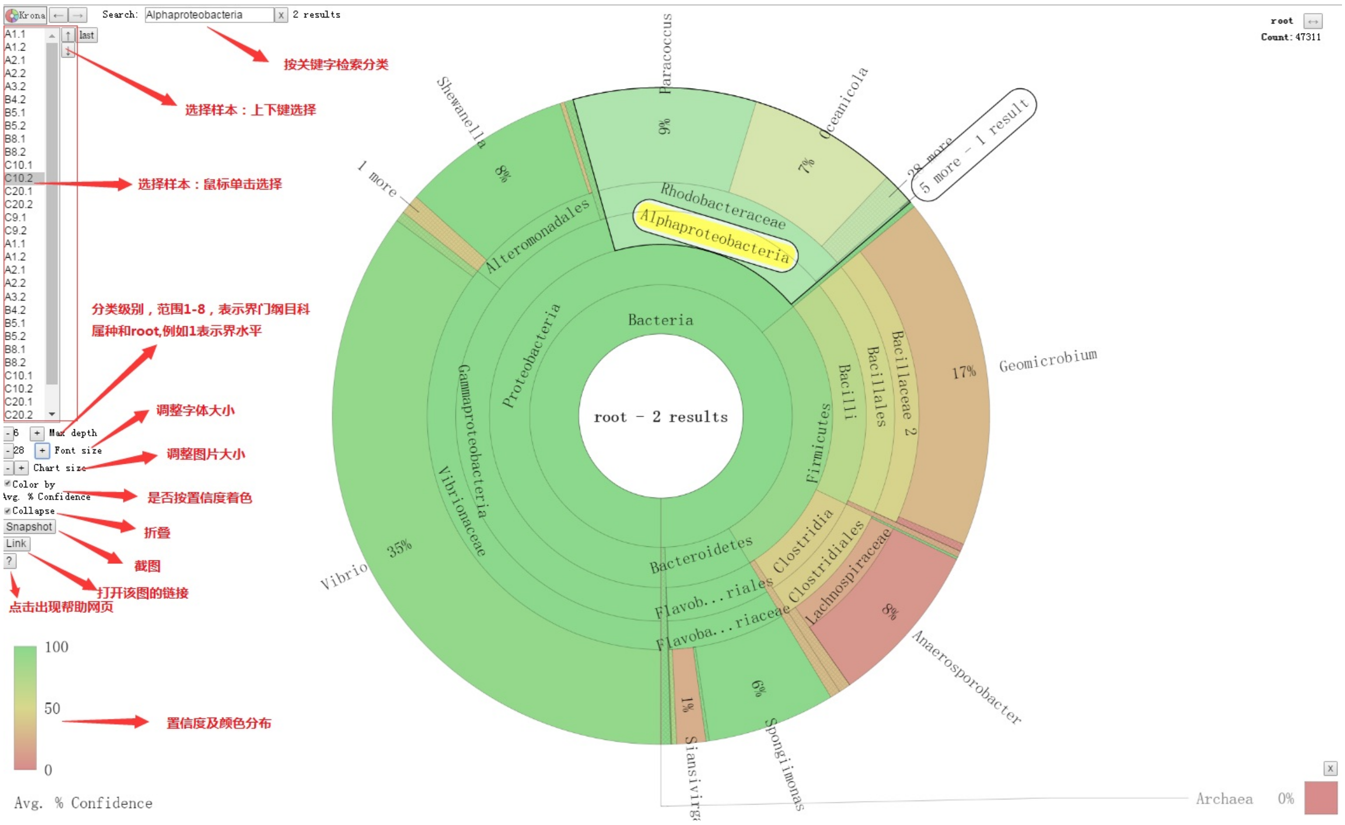


**图3.7 物种注释结果可视化展示**

ReadMe

## 物种相关性结果展示

根据物种在各个样本中的丰度及变化情况，用CCREPE（http://huttenhower.sph.harvard.edu/ccrepe ）进行Spearman秩相关性分析，计算物种之间的相关性，然后挑选出前100组，用Cytoscape(http://chianti.ucsd.edu/cytoscape-3.2.1/)软件进行展示，选择属水平物种间相关性展示如下：

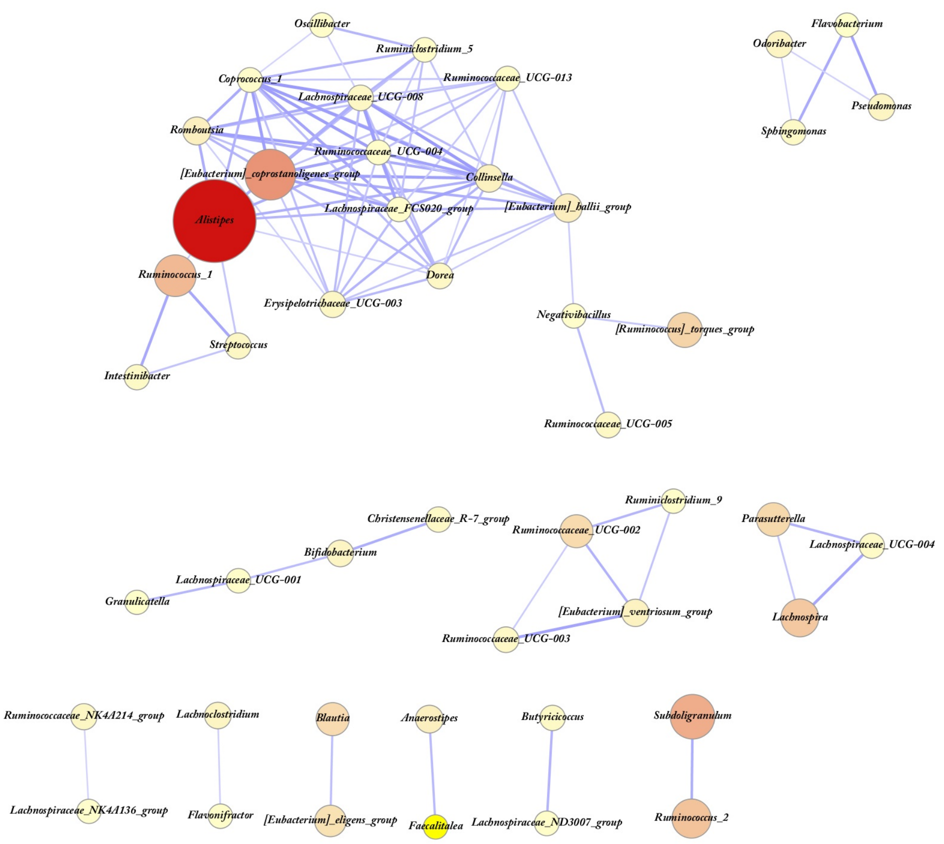


**图3.8 属水平相关性结果可视化展示**

说明：图中每个节点代表一个物种，节点颜色的深浅和大小表示丰度的高低；相互关联的物种用线连接（红色表示为正相关性，绿色表示为负相关性），线的粗细和颜色的深浅代表两个物种相关性（绝对值）的高低。

结果目录见：02.OTUanalysis/cor/

## 3.3 Tax4Fun功能分析

为了研究样品的物种的功能，使用Tax4Fun(http://tax4fun.gobics.de< /a>)软件对KEGG功能进行预测，然后根据样本间的功能丰度，做组间差异分析。

### 3.3.1 KEGG注释结果

表3.6 KEGG 功能注释结果

| #OTU ID | C1 | C2 | C3 | ... | SampleN |
|---|---|---|---|---|---|
| Metabolism;Carbohydrate metabolism;Glycolysis / Gluconeogenesis | 0.008306 | 0.008772 | 0.009079 | ... | ... |
| Metabolism;Carbohydrate metabolism;Citrate cycle (TCA cycle) | 0.004355 | 0.004386 | 0.005559 | ... | ... |
| Metabolism;Carbohydrate metabolism;Pentose phosphate pathway | 0.006854 | 0.007310 | 0.007347 | ... | ... |
| Metabolism;Carbohydrate metabolism;Pentose and glucuronate interconversions | 0.010873 | 0.009906 | 0.008831 | ... | ... |
| Metabolism;Carbohydrate metabolism;Fructose and mannose metabolism | 0.021360 | 0.019032 | 0.021742 | ... | ... |

样本的KEGG丰度对应图表的结果见：03.Tax4Fun/taxa_summary_plots

### 3.3.2 T-test组间KEGG功能差异分析

针对有分组的项目，可以通过功能差异统计分析进行深入研究。通过统计分析，可以有针对性的找出分组间丰度变化差异显著的KEGG功能，并得到差异功能在不同分组间的富集情况。

**表3.7 T test组间差异表**

| KEGG descrip... | avg(CHB) | sd(CHB) | avg(C) | sd(C) | p.value | interval lower | interval upper | q.values |
|---|---|---|---|---|---|---|---|---|
| Metabolism;C... | 0.009080 | 0.000200 | 0.008719 | 0.000389 | 0.268246 | -0.00121 | 0.000489 | 0.524996 |
| Metabolism;C... | 0.005316 | 0.000126 | 0.004767 | 0.000686 | 0.298688 | -0.00215 | 0.001056 | 0.560552 |
| Metabolism;C... | 0.007679 | 6.95e-05 | 0.007170 | 0.000274 | 0.074842 | -0.00112 | 0.000110 | 0.384967 |
| Metabolism;C... | 0.010546 | 0.000177 | 0.009870 | 0.001021 | 0.370695 | -0.00307 | 0.001726 | 0.626978 |
| Metabolism;C... | 0.023230 | 5.25e-05 | 0.020711 | 0.001466 | 0.096602 | -0.00615 | 0.001114 | 0.418219 |
| Metabolism;C... | 0.017436 | 2.20e-05 | 0.012765 | 0.001225 | 0.022129 | -0.00771 | -0.00162 | 0.216554 |
| Metabolism;C... | 0.001390 | 0.000130 | 0.001461 | 0.000229 | 0.690093 | -0.00044 | 0.000584 | 0.804620 |
| Metabolism;L... | 0.006725 | 0.000110 | 0.005640 | 0.001107 | 0.230546 | -0.00378 | 0.001611 | 0.489687 |
| Metabolism;L... | 1.73e-06 | 7.63e-07 | 3.58e-06 | 2.95e-06 | 0.395187 | -4.8e-06 | 8.50e-06 | 0.641878 |

KEGG description表示对应的代谢通路信息；avg，sd，分别是对应组的平均值和标准差；p value是假设检验的p值，interval lower是置信区间的下限值，interval upper是置信区间的上 限值，q value是p value矫正的q 值。

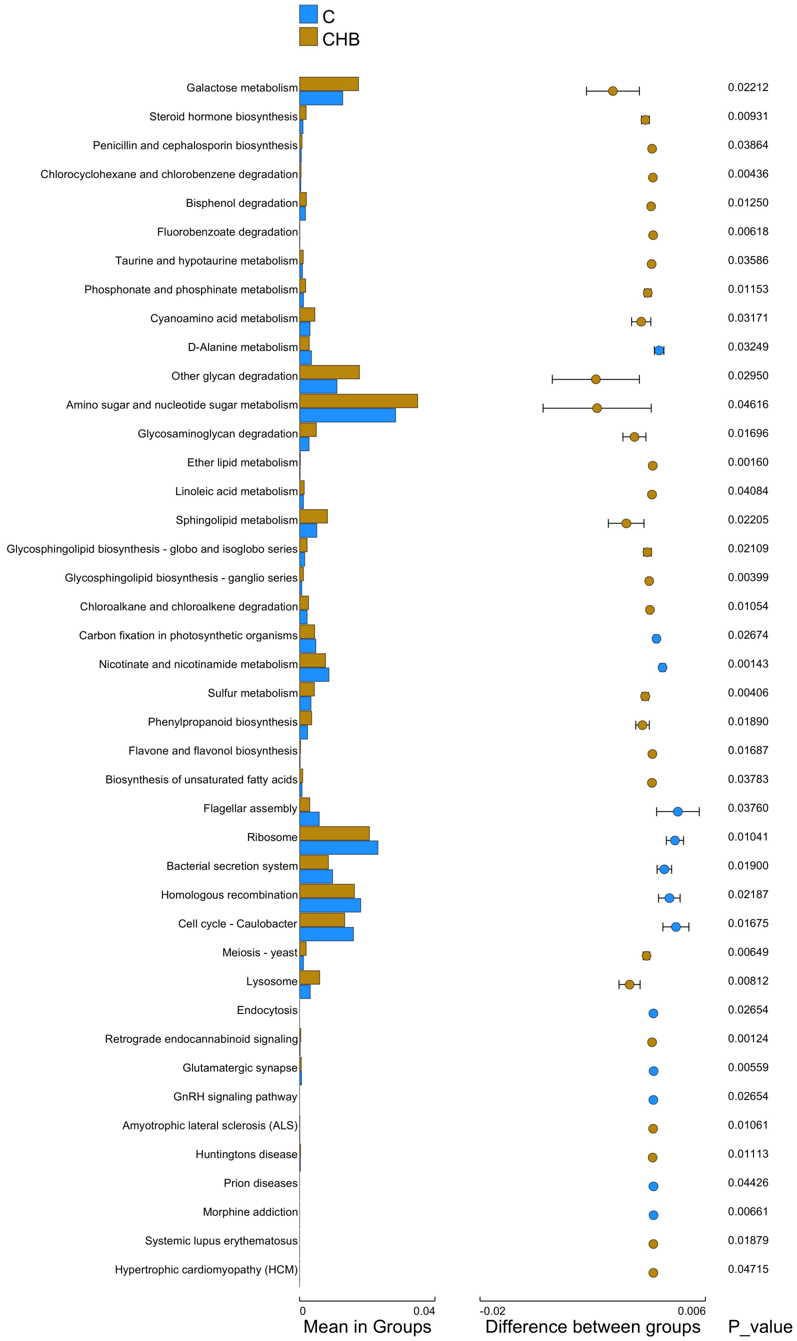


**图3.9 T_test组间功能差异分析图**

说明：左图为组间差异KEGG功能丰度展示，图中每个条形分别表示在分组间丰度差异显著的功能在每个组中的均值。 右图为组间差异置信度展示，图中每个圈的最左端点表示均值差的95%置信区间下限，圆圈的最右端点表示均值差95%置信区间上限。圆圈的圆心代表的是均值的差。圆圈颜色所代表的组为均值高的 组。展示结果的最右端是对应差异功能的组间显著性检验p值。

## 3.4 样品复杂度分析（Alpha Diversity）

Alpha Diversity 用于分析样品内（Within-community）的微生物群落多样性[6]，通过单样本的多样性分析（Alpha 多样性）可以反映样品内的微生物群落的丰富度和多样性，包括用物种累积曲线、物种多样性曲线和一系列统计学分析指数来评估各样品中微生物群落的物种丰富度和多样性的差异。

### 3.4.1 Alpha Diversity指数表格

一般来说，在97%以上的序列一致性下聚类成为一个OTU的序列被认为可能是源自于同一个种（Species Boundary）的序列。因此，对不同样品在97%一致性阈值下的Alpha Diversity 分析指数进行统计。结果如下：

**表3.8 Alpha Indices 统计表**

| | PD_whole_tree | observed_species | shannon | simpson | ace | chao1 | goods_coverage |
|---|---|---|---|---|---|---|---|
| C1 | 8.094129 | 122.0 | 3.616030 | 0.855734 | 144.6440 | 145.0 | 0.999305 |
| C2 | 10.47953 | 183.0 | 5.249594 | 0.949603 | 203.8450 | 223.625 | 0.999247 |
| C3 | 7.022279 | 130.0 | 4.357653 | 0.898227 | 133.9765 | 133.6 | 0.999739 |
| CHB10 | 7.315819 | 120.0 | 3.576939 | 0.809828 | 135.8919 | 141.375 | 0.999449 |
| CHB13 | 9.61633 | 153.0 | 3.997646 | 0.845138 | 166.8155 | 164.4 | 0.999449 |
| H10 | 7.036729 | 107.0 | 3.675570 | 0.874651 | 121.7268 | 120.6 | 0.999507 |
| H8 | 6.505189 | 86.0 | 2.982340 | 0.805039 | 107.0736 | 109.75 | 0.999420 |
| IT20 | 8.82985 | 132.0 | 3.672653 | 0.816585 | 143.2167 | 143.6666 | 0.999565 |
| IT22 | 6.713119 | 102.0 | 3.695175 | 0.882384 | 114.6420 | 110.75 | 0.999565 |

*ReadMe*

结果文件见：04.AlphaDiversity/alpha_diversity_index.xls

### 3.4.2 物种多样性曲线

稀释曲线和Rank abundance曲线是常见的描述组内样品多样性的曲线。Rarefaction Curve，即稀释曲线，是从样品中随机抽取一定测序量的数据，统计它们所代表物种数目（即OTUs数目），以抽取的测序数据量与对应的物种数来构建曲线。稀释曲线可直接反映测序数据量的合理性，并间接反映样品中物种的丰富程度，当曲线趋向平坦时，说明测序数据量渐进合理，更多的数据量只会产生少量新的物种（OTUs）。

Rank Abundance曲线是将样品中的OTUs按相对丰度（或者包含的序列数目）由大到小排序得到对应的排序编号，再以OTUs的排序编号为横坐标，OTUs中的相对丰度（也可用该等级OTU中序列数的相对百分含量）为纵坐标,将这些点用折线连接，即绘制得到Rank Abundance曲线，它可直观的反映样品中物种的丰富度和均匀度。在水平方向上，物种的丰富度由曲线的宽度来反映，物种的丰富度越高，曲线在横轴上的跨度越大；在垂直方向上，曲线的平滑程度，反映了样品中物种的均匀程度，曲线越平缓，物种分布越均匀。

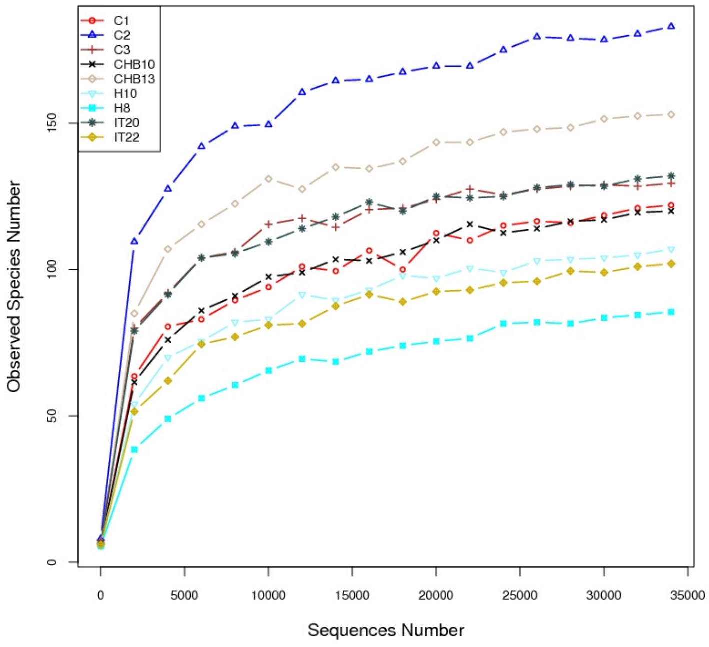


图3.10 稀释曲线

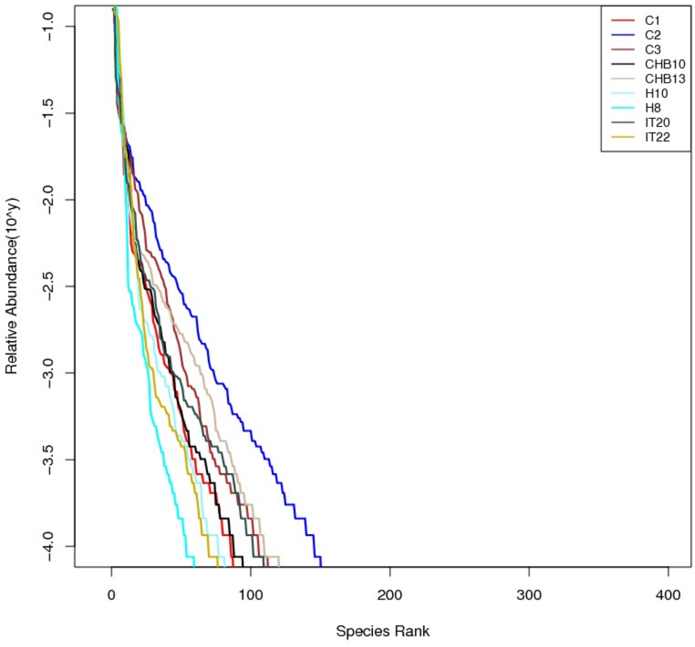


图3.11 Rank Abundance 曲线

左图为稀释曲线，右图为Rank Abundance 曲线，横坐标为从某个样品中随机抽取的测序条数，纵坐标为基于该测序条数能构建的OTU数量，用来反映测序深度情况，不同的样品使用不同颜色的曲线表示；Rank Abundance 曲线中，横坐标为按OTUs丰度排序的序号，纵坐标为对应的OTUs的相对丰度，不同的样品使用不同的颜色的折线表示。

结果目录见：04.AlphaDiversity/observed_species和04.AlphaDiversity/Rank_Abundance

### 3.4.3 稀释曲线网页化展示

我们使用QIIME中的**make_rarefaction_plots.py**做稀释曲线的网页化展示，结果见文件**rarefaction_plots.html**

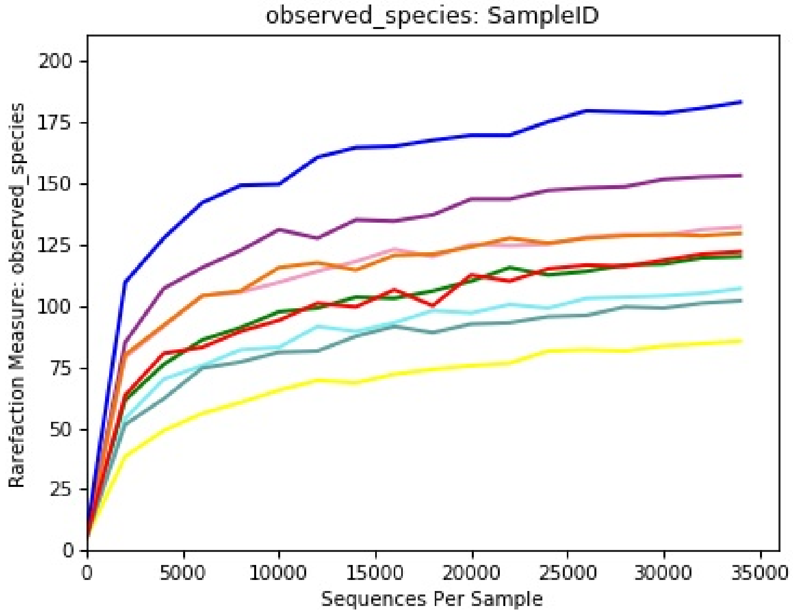


**图3.12 稀释曲线可视化展示**

结果目录：04.AlphaDiversity/make_rarefaction_plots

### 3.4.4 基于OTU的Venn图

**基于OTU的venn图**

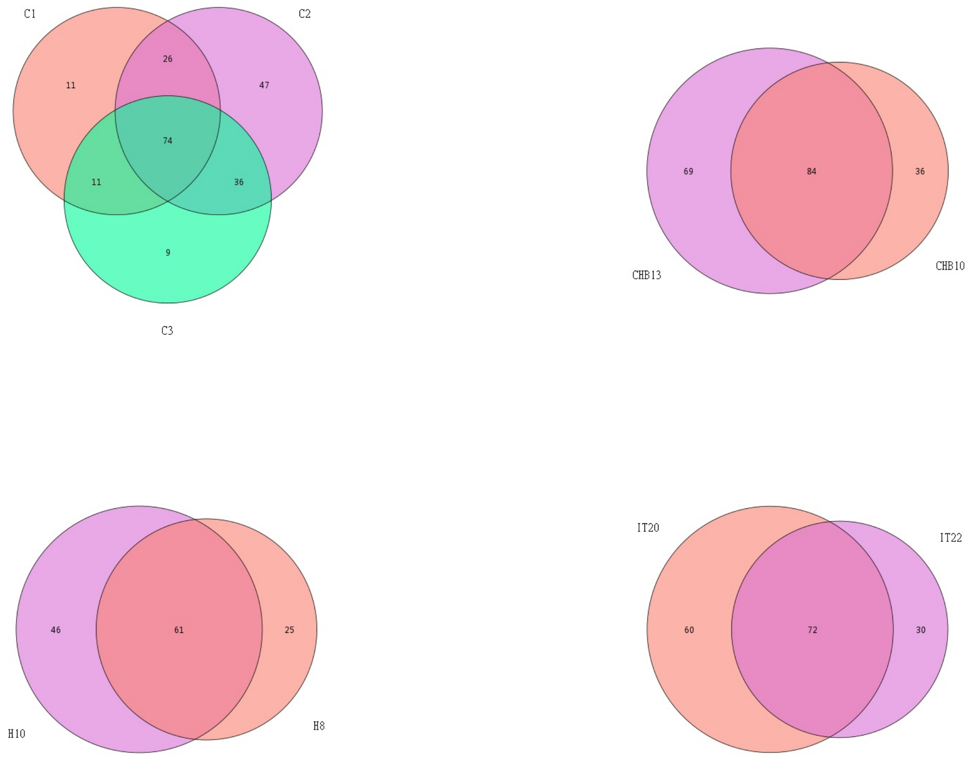


**图3.13 维恩图**

图中每个圈代表一个（组）样品，圈和圈重叠部分的数字代表样本（组）之间共有的OTUs个数，没有重叠部分的数字代表样本（组）的特有OTUs个数

结果目录见：04.AlphaDiversity/venn_figure/

### 3.4.5 Alpha多样性指数组间差异分析

Alpha多样性指数组间差异分析中，箱形图可以直观的反应组内物种多样性的中位数、离散程度、最大值、最小值、异常值（箱形图的解读请查看帮助）。同时，通过wilcox秩和检验和Tukey检验（只有2个分组时进行T-test和wilcox秩和检验，分组大于2时进行Tukey和wilcox秩和检验）分析组间物种多样性差异是否显著。 其组间差异分析的箱形图如下：

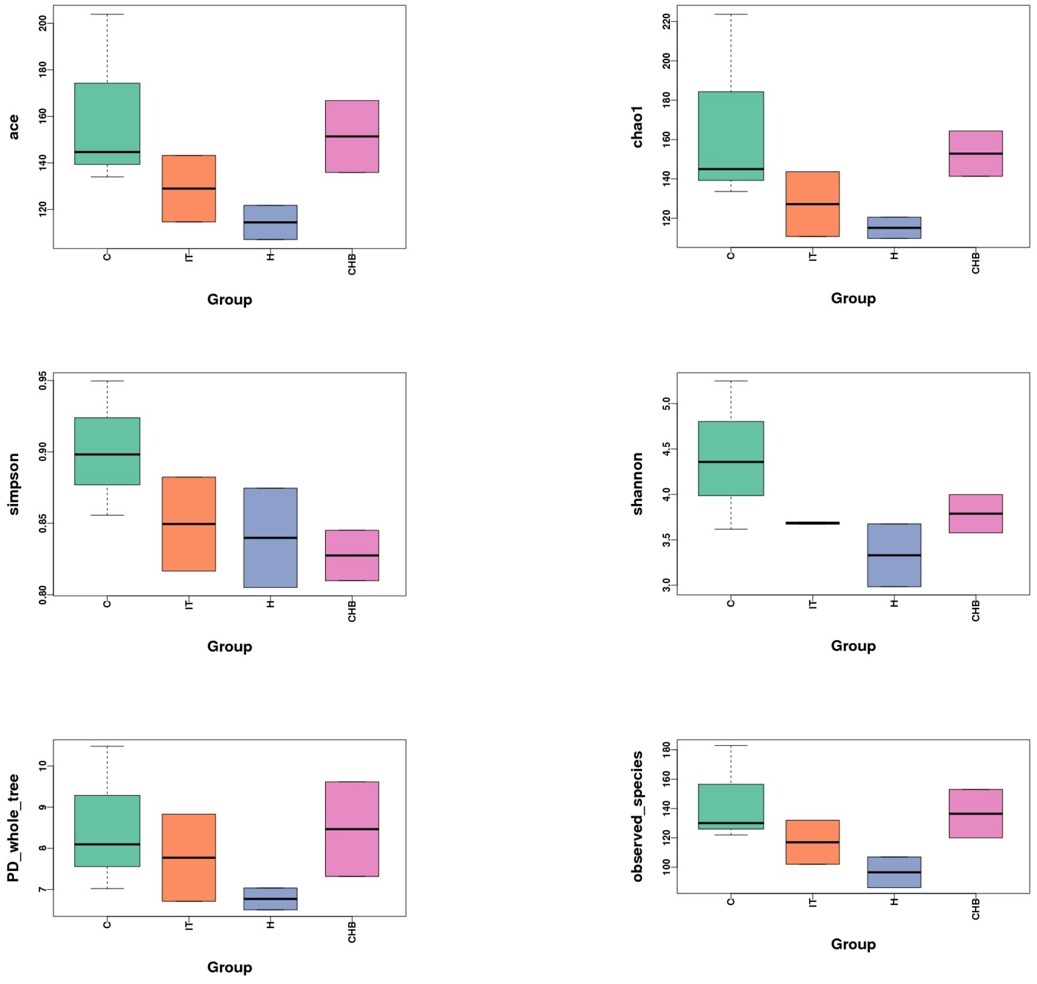


**图3.14 6种Alpha多样性指数组间差异的箱形图,从上到下，从左至右，依次为ace，chao1，simpson，shannon，PD_whole_tree，observed_species**

6种Alpha多样性指数组间差异的箱形图:

04.AlphaDiversity/Alpha_index/{ace，chao1，PD_whole_tree，observed_species，shannon，simpson}/*.box.{pdf,png}

差异显著性检验的结果见:

04.AlphaDiversity/Alpha_index/{ace,chao1,PD_whole_tree,observed_species,shannon,simpson}/*.{_wilcox.txt,_Tukey.txt}

## 3.4 多样品比较分析（Beta Diversity）

Beta Diversity是对不同样品的微生物群落构成进行比较分析。首先根据所有样品的物种注释结果和OTUs的丰度信息，将相同分类的OTUs信息合并处理得到物种丰度信息表（Profiling Table）。 同时利用OTUs之间的系统发生关系，进一步计算Unifrac距离（Unweighted Unifrac）。Unifrac 距离是一种利用各样品中微生物序列间的进化信息计算样品间距离，两个以上的样品，则得到一个距离矩阵。 然后，利用OTUs的丰度信息对Unifrac距离（Unweighted Unifrac）进一步构建Weighted Unifrac距离[10]。最后，通过多变量统计学方法主成分分析（PCA，Principal Component Analysis），主坐标分析（PCoA，Principal Co-ordinates Analysis）， 无度量多维标定法（NMDS，Non-Metric Multi-Dimensional Scaling），非加权组平均聚类分析（UPGMA，Unweighted Pair-group Method with Arithmetic Means）分析以及Beta多样性指数组间差异分析等方法，从中发现不同样品（组）间的差异。

### 3.5.1 系统进化关系展示

为了进一步研究OTUs的系统进化关系以及后续Beta多样性指数研究的需要，通过多序列比对得到所有OTUs代表序列的系统发生关系。分别选取相对丰度排名前10的属所对应的OTUs、相对丰度排名前50的OTUs的系统发生关系数据进行分析，相对丰度排名前50的OTUs进化关系如下图：

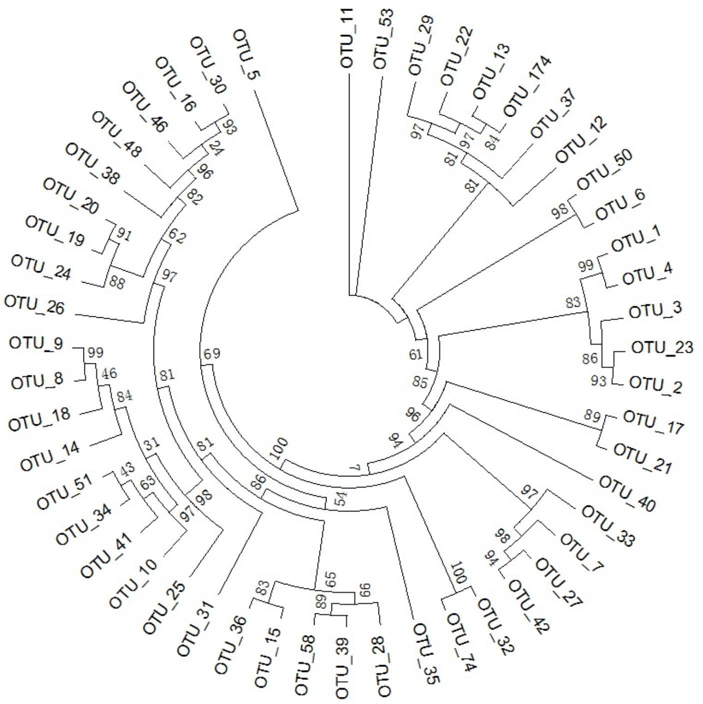


**图3.15 OTUs的系统发生关系**

系统进化关系图：05.BetaDiversity/phylo_tree/select(.OTUs.top50).tre.png

### 3.5.2 Beta多样性指数

Beta多样性研究中，选用 Weighted Unifrac距离和 Unweighted Unifrac 两个指标来衡量两个样品间的相异系数，其值越小，表示这两个样品在物种多样性方面存在的差异越小。以 Weighted Unifrac 和 Unweighted Unifrac 距离绘制的Heatmap展示结果如下图：

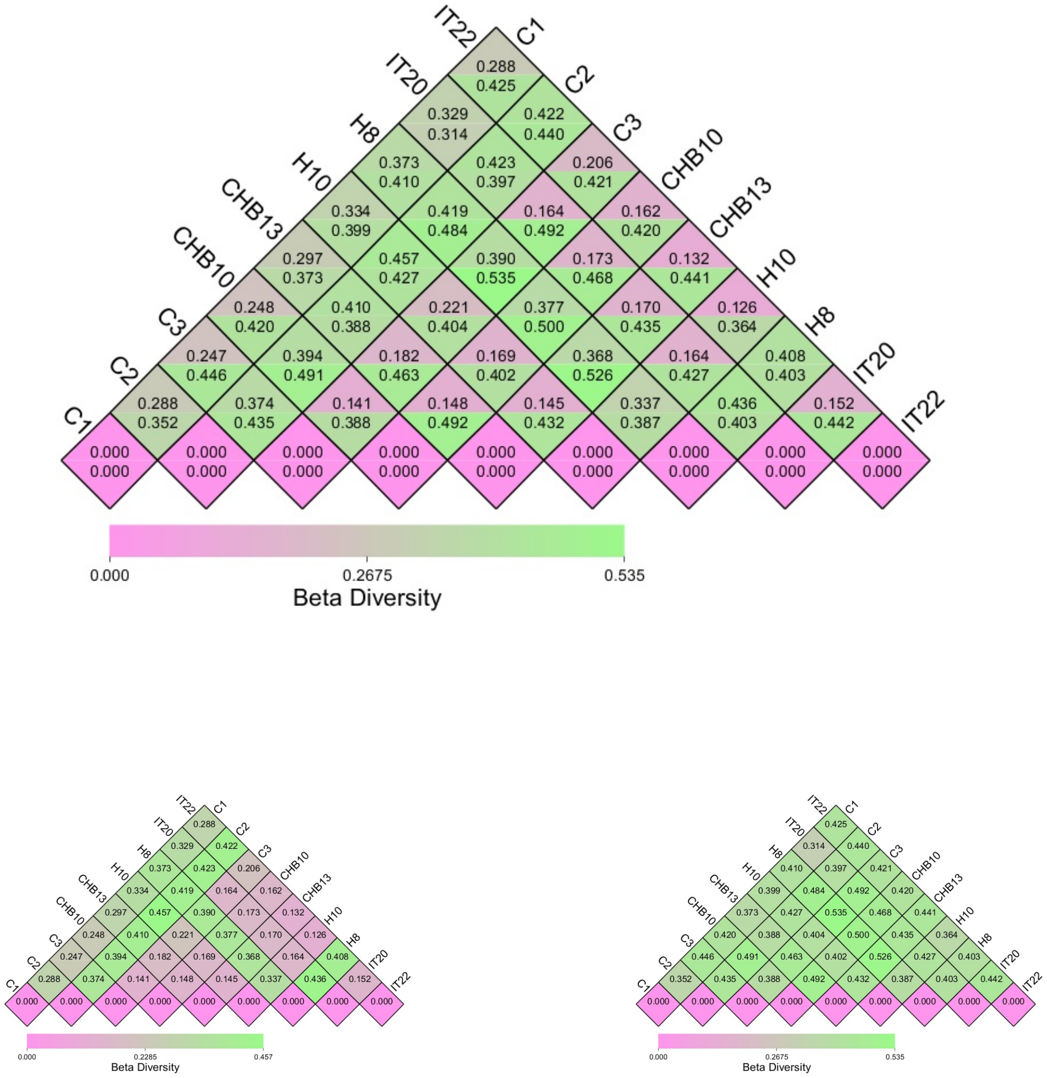


**图3.16 Beta多样性指数热图**

上方的图中方格中的数字是样品两两之间的相异系数，相异系数越小的两个样品，物种多样性的差异越小；同一方格中，上下两个值分别代表Weighted Unifrac和Unweighted Unifrac距离;下方左侧图是Weighted Unifrac指数热图，右侧是Unweighted Unifrac指数热图

结果目录见：05.BetaDiversity/beta_heatmap/beta_diversity.heatmap.*.png

### 3.5.3 PCA分析

主成分分析（PCA，Principal Component Analysis），是一种应用方差分解，对多维数据进行降维，从而提取出数据中最主要的元素和结构的方法。应用PCA分析， 能够提取出最大程度反映样品间差异的两个坐标轴，从而将多维数据的差异反映在二维坐标图上，进而揭示复杂数据背景下的简单规律。如果样品的群落组成越相似，则它们在PCA图中的距离越接近。基于OTU水平的PCA分析结果展示见下图：

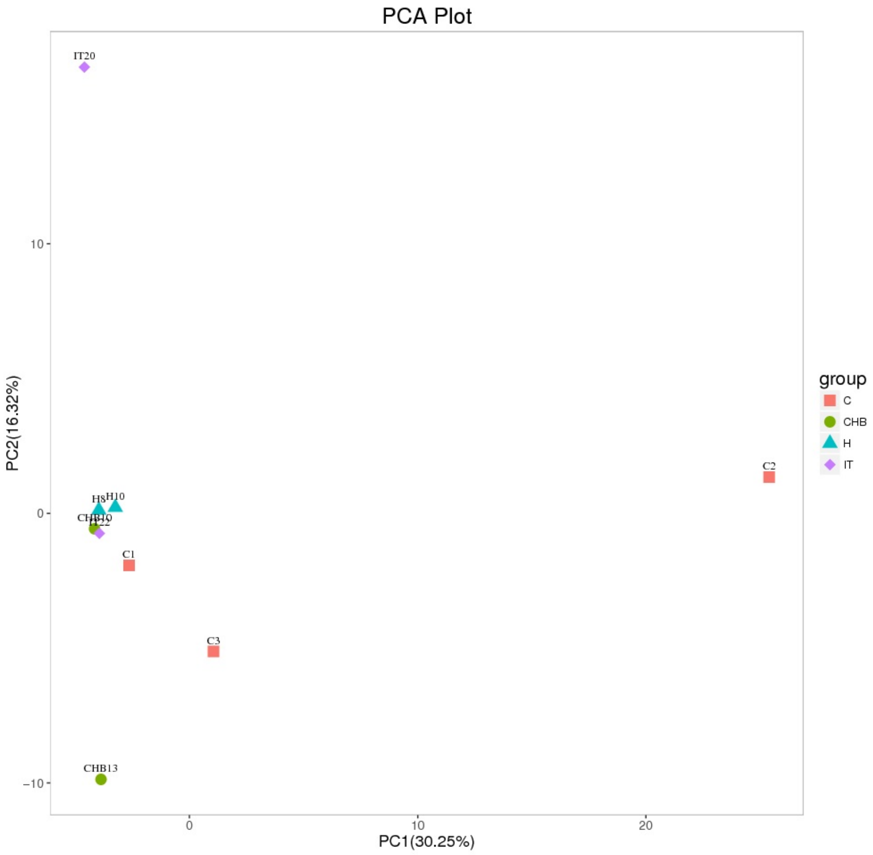


**图3.17 PCA分析**

横坐标表示第一主成分，百分比则表示第一主成分对样品差异的贡献值；纵坐标表示第二主成分，百分比表示第二主成分对样品差异的贡献值；图中的每个点表示一个样品，同一个组的样品使用同一种颜色表示；在有聚类圈的PCA图中，以分组信息添加聚类圈。

结果目录见：05.BetaDiversity/PCA/

### 3.5.4 PCoA分析

主坐标分析（PCoA，Principal Co-ordinates Analysis），是一种与PCA类似的降维排序方法，通过一系列的特征值和特征向量排序从多维数据中提取出最主要的元素和结构。区别在于PCA是基于样品的相似系数矩阵来寻找主坐标，而PCoA是基于距离矩阵来寻找主坐标。我们基于Weighted Unifrac距离和Unweighted Unifrac 距离来进行PCoA分析，并选取贡献率最大的主坐标组合进行作图展示。如果样品距离越接近，表示物种组成结构越相似，因此群落结构相似度高的样品倾向于聚集在一起，群落差异很大的样品则会远远分开。结果展示如下：

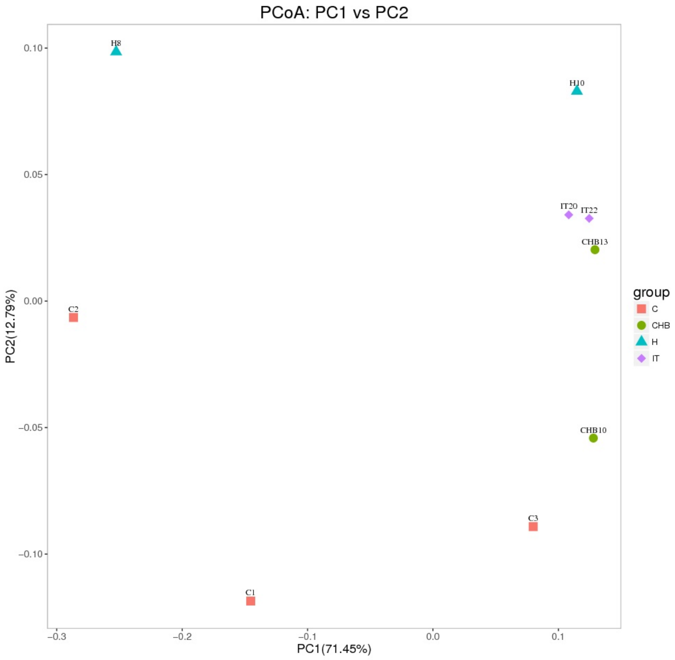


图3.18 基于Weighted Unifrac 距离PCoA分析

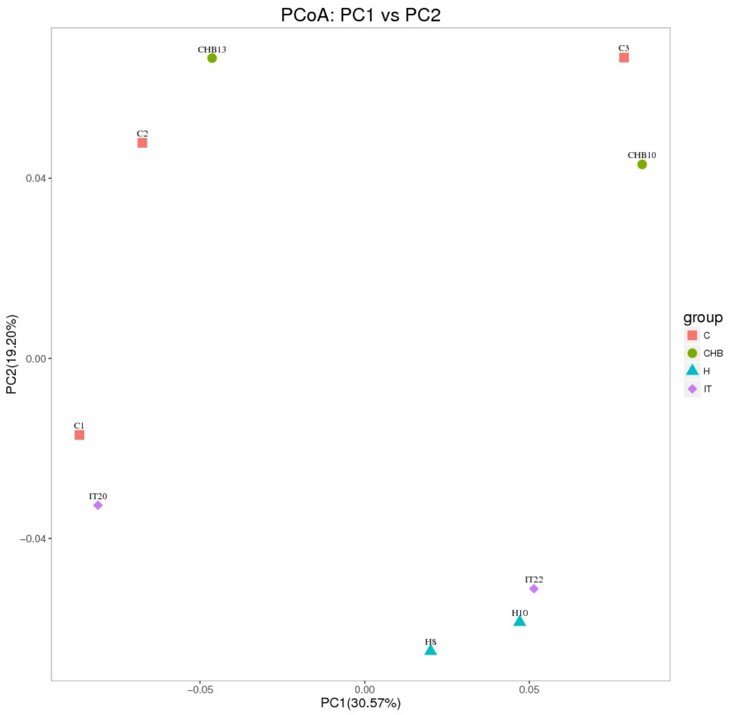


图3.19 基于Unweighted Unifrac 距离PCoA分析

横坐标表示一个主成分，纵坐标表示另一个主成分，百分比表示主成分对样品差异的贡献值；图中的每个点表示一个样品，同一个组的样品使用同一种颜色表示。

结果目录见：05.BetaDiversity/PCoA/(un)weighted_unifrac/

### 3.5.5 NMDS分析

无度量多维标定法（NMDS，Non-Metric Multi-Dimensional Scaling）统计是一种适用于生态学研究的排序方法。NMDS是非线性模型，其设计目的是为了克服线性模型（包括PCA、 PCoA）的缺点，更好地反映生态学数据的非线性结构[12]。应用NMDS分析，根据样本中包含的物种信息，以点的形式反映在多维空间上，而对不同样本间的差异程度，则是通过点与点间的距离体现，能够反映样本的组间和组内差异等。 基于OTU水平的NMDS分析结果展示见下图：

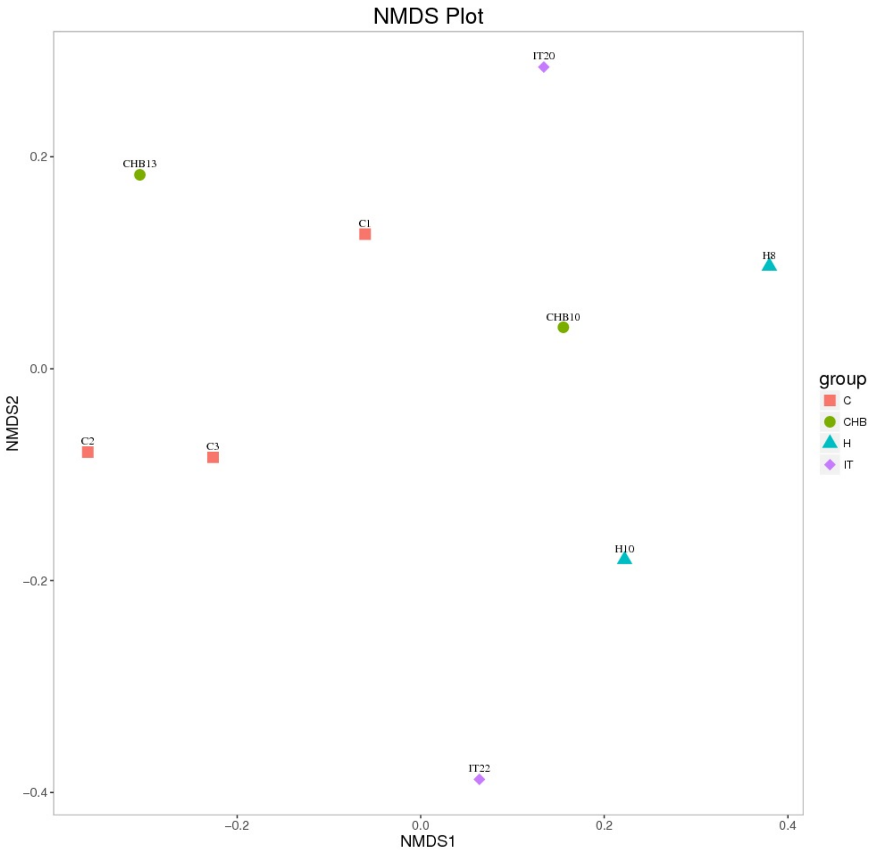


**图3.20 NMDS分析**

图中的每个点表示一个样品，点与点之间的距离表示差异程度，同一个组的样品使用同一种颜色表示。Stress小于0.2时，表明NMDS分析具有一定的可靠性。

结果目录见：05.BetaDiversity/NMDS

### 3.5.6 样品聚类分析

为了研究不同样品间的相似性，还可以通过对样品进行聚类分析，构建样品的聚类树。在环境生物学中，UPGMA（Unweighted Pair-group Method with Arithmetic Mean）是一种较为常用的聚类分析方法，它最早便是用来解决分类问题的。UPGMA 的基本思想是：首先将距离最小的 2 个样品聚在一起，并形成一个新的节点（新的样品），其分支点位于 2 个样品间距离的 1/2 处；然后计算新的 “样品” 与其它样品间的平均距离，再找出其中的最小 2 个样品进行聚类；如此反复，直到所有的样品都聚到一起，最终得到一个完整的聚类树。

以Weighted Unifrac距离矩阵和Unweighted Unifrac距离矩阵做UPGMA聚类分析，并将聚类结果与各样品在门水平上的物种相对丰度整合展示，见下方结果图：

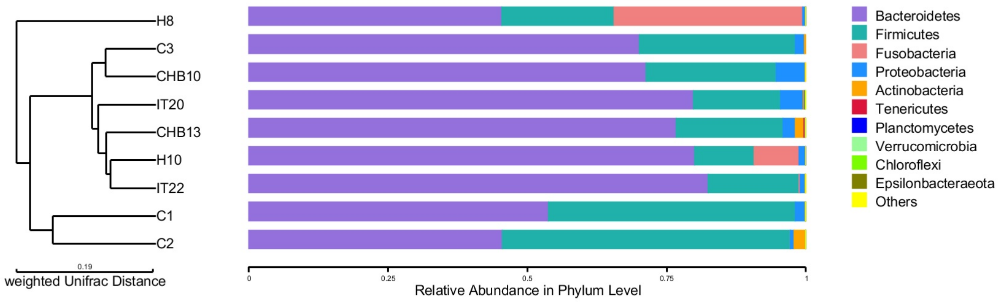


**图3.21 基于Weighted Unifrac距离的UPGMA聚类树**

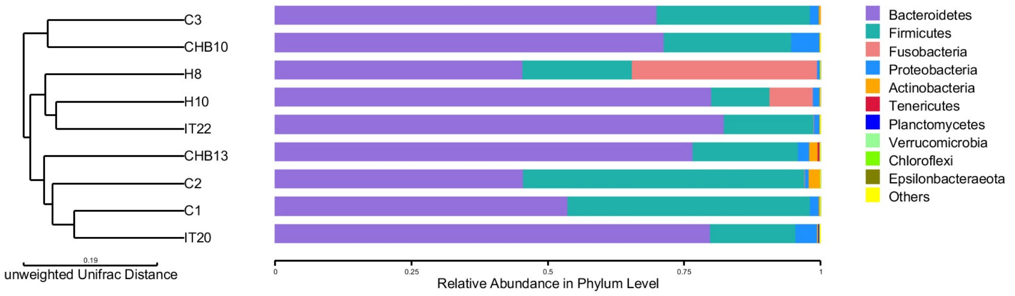


**图3.22 基于Unweighted Unifrac距离的UPGMA聚类树**

左侧是UPGMA聚类树结构，右侧的是各样品在门水平上的物种相对丰度分布图。

结果目录见：05.BetaDiversity/beta_tree/UPGMA.(un)weighted.tree.png

### 3.5.7 Beta多样性指数组间差异分析

将多组样本的Unifrac距离进行四分位计算，比较不同样本组的组内和组间的距离分布差异。箱形图由五个部分组成，分别是最小值、第一四分位数、中位数、第三四分位数以及最大值。它可以粗略地看出数据是否具有对称性，分布的离散程度等信息。（箱形图的解读请查看箱形图）。同时，通过T-test检验，wilcox秩和检验和Tukey检验（只有2个分组时进行T-test和wilcox秩和检验，分组大于2时进行Tukey和wilcox秩和检验）分析组间物种Beta多样性差异是否显著。Unifrac距离差异分析的箱形图展示如下:

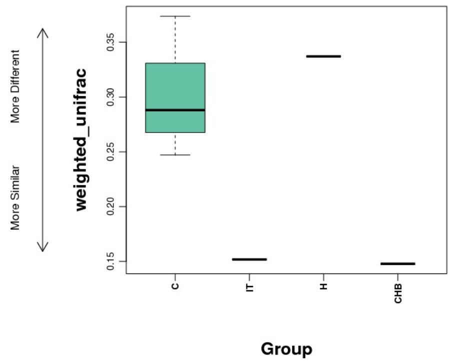


图3.23 基于Weighted Unifrac 组内距离分布差异的箱形图

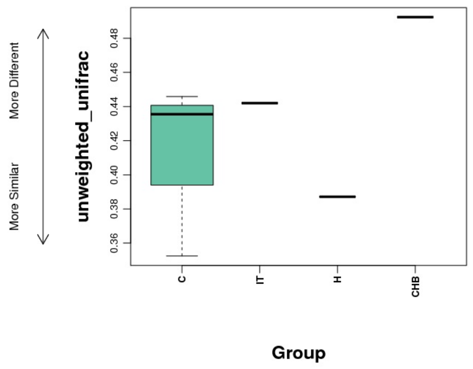


图3.24 基于Unweighted Unifrac 组内距离分布差异的箱形图

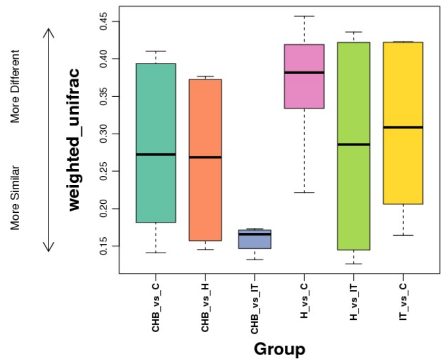


图3.25 基于Weighted Unifrac 组间距离分布差异的箱形图

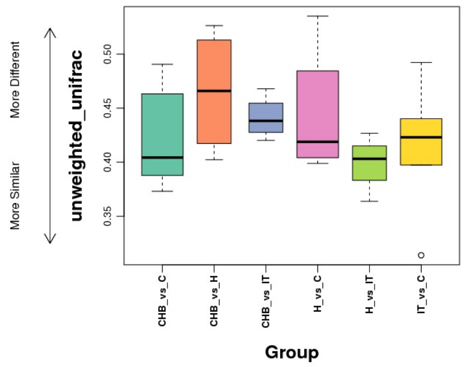


图3.26 基于Unweighted Unifrac 组间距离分布差异的箱形图

结果目录见：05.BetaDiversity/beta_div/(un)weighted_unifrac.within（between）.png

## 3.6 统计分析（Statistics）

针对有分组的项目，可以通过群落结构差异统计分析进行深入研究。通过统计分析，可以有针对性的找出分组间丰度变化差异显著的物种，并得到差异物种在不同分组间的富集情况，同时，可以比较组内差异和组间差异的大小，判断不同分组间的群落结构差异是否具有显著意义。

### 3.6.1 T-test组间物种差异分析

为了寻找各分类水平（门Phylum、纲Class、目Order、科Family、属Genus、种Species）下，组间的差异物种，做组间的T-test检验，找出差异显著（p值<=0.05)的物种。取其中一水平下T test 原始结果文件的前10行作为展示：

**表3.9 T test组间差异表**

| Taxa | avg(CHB) | sd(CHB) | avg(C) | sd(C) | p.value | interval lower | interval upper | q.values |
|---|---|---|---|---|---|---|---|---|
| k__Bacteria;... | 0.739779 | 0.037977 | 0.564228 | 0.125107 | 0.124854 | -0.45135 | 0.100252 | 0.645812 |
| k__Bacteria;... | 0.213069 | 0.029604 | 0.413679 | 0.121232 | 0.093664 | -0.07410 | 0.475321 | 0.645812 |
| k__Bacteria;... | 0 | 0 | 0.000279 | 0.000484 | 0.422649 | -0.00092 | 0.001484 | 0.645812 |
| k__Bacteria;... | 0.036742 | 0.021210 | 0.013289 | 0.006093 | 0.351889 | -0.17841 | 0.131506 | 0.645812 |
| k__Bacteria;... | 0.008454 | 0.010072 | 0.008377 | 0.011009 | 0.994158 | -0.03470 | 0.034550 | 1 |
| k__Bacteria;... | 0.001201 | 0.001699 | 0 | 0 | 0.5 | -0.01646 | 0.014065 | 0.645812 |
| k__Bacteria;... | 2.895361 | 4.094659 | 2.895361 | 2.895361 | 1 | -0.00017 | 0.000173 | 1 |
| k__Bacteria;... | 0.000492 | 0.000696 | 6.755843 | 6.027175 | 0.546456 | -0.00654 | 0.005700 | 0.645812 |
| k__Bacteria;... | 0 | 0 | 1.930241 | 1.671637 | 0.183503 | -2.22233 | 6.082819 | 0.645812 |

Taxa是物种分类信息；avg，sd，分别是对应组的平均值和标准差；p value是假设检验的p值，interval lower是置信区间的下限值，interval upper是置信区间的上限值，q value是p value矫正的q 值。

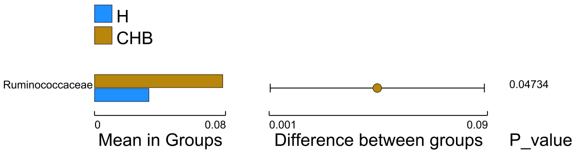


**图3.27 T_test组间物种差异分析图**

说明：左图为组间差异物种丰度展示，图中每个条形分别表示在分组间丰度差异显著的物种在每个组中的均值。 右图为组间差异置信度展示，图中每个圈的最左端点表示均值差的95%置信区间下限，圆圈的最右端点表示均值差95%置信区间上限。圆圈的圆心代表的是均值的差。圆圈颜色所代表的组为均值高的组。展示结果的最右端是对应差异物种的组间显著性检验p值。

各分类层级（phylum、class、order、family、genus、species）的T_test组间物种差异分析结果见：06.StatTest/T.test/
以门水平为例，筛选显著差异后的表格见：06.StatTest/T.test/phylum/*.Psig.xls和*.Qsig.xls

### 3.6.2 Metastat分析

根据样品中物种丰度信息，运用permutation test （置换次数 为999）检测组间微生物群落中具有显著性差异的物种，进行稀有频率数据的多重假设检验和错误发现率（FDR）分析评估观察到差异的显著性。以门（Phylum）水平的组间物种差异显著性分析为例，物种差异分析统计结果示例如下表（取门水平下的分析结果作为展示）。

**表3.10 MetaStat分析**

| Taxa | mean(CHB) | variance(CHB) | standard error(CHB) | mean(C) | variance(C) | standard error(C) | p.value | FDR |
|---|---|---|---|---|---|---|---|---|
| k__Bacteria;... | 0.739779 | 0.001442 | 0.026854 | 0.564228 | 0.015651 | 0.072230 | 0.188811 | 0.626262 |
| k__Bacteria;... | 0.213069 | 0.000876 | 0.020933 | 0.413679 | 0.014697 | 0.069993 | 0.188811 | 0.626262 |
| k__Bacteria;... | 0 | 0 | 0 | 0.000279 | 2.35e-07 | 0.000279 | 1 | 1 |
| k__Bacteria;... | 0.036742 | 0.000449 | 0.014997 | 0.013289 | 3.71e-05 | 0.003517 | 0.093906 | 0.626262 |
| k__Bacteria;... | 0.008454 | 0.000101 | 0.007122 | 0.008377 | 0.000121 | 0.006356 | 1 | 1 |
| k__Bacteria;... | 0.001201 | 2.88e-06 | 0.001201 | 0 | 0 | 0 | 0.402597 | 0.626262 |
| k__Bacteria;... | 2.89e-05 | 1.67e-09 | 2.89e-05 | 2.89e-05 | 8.38e-10 | 1.67e-05 | 1 | 1 |
| k__Bacteria;... | 0.000492 | 4.84e-07 | 0.000492 | 6.75e-05 | 3.63e-09 | 3.47e-05 | 0.402597 | 0.626262 |
| k__Bacteria;... | 0 | 0 | 0 | 1.93e-05 | 2.79e-10 | 9.65e-06 | 0.379620 | 0.626262 |

Taxa是物种分类信息；Mean，Variance，standard error 对应各组的平均值，方差和标准差；P value是假设检验的p值，FDR是P value矫正后的值。可以根据P value<=0.05和FDR<=0.05来筛选得到显著差异基因。

各分类层级（phylum、class、order、family、genus）的T_test组间物种差异分析结果见：06.StatTest/MetaStat
以门水平为例，筛选显著差异后的表格见：06.StatTest/MetaStat/phylum/*.Psig.xls和*.Qsig.xls

### 3.6.3 Anosim和MRPP分析

Anosim和MRPP分析用于比较组间群落结构差异是否显著性，同时可评判组内差异和组间差异的大小。

**Anosim分析**

Anosim分析是一种非参数检验，用来检验组间的差异是否显著大于组内差异，从而判断分组是否有意义，详细计算过程可查看R软件的说明。分析结果见下表。

**表3.11 Anosim分析结果**

| Group | R-value | P-value |
|---|---|---|
| IT-VS-C | 0.083333 | 0.6 |
| H-VS-C | 0.75 | 0.1 |
| CHB-VS-C | 0.083333 | 0.5 |
| H-VS-IT | 0.5 | 0.333333 |
| CHB-VS-IT | -0.5 | 1 |
| CHB-VS-H | 0 | 0.666666 |

R-value介于[-1，1]之间，R-value大于0，说明组间差异显著。R-value小于0，说明组内差异大于组间差异，统计分析的可信度用 P-value 表示，P< =0.05 表示统计具有显著性。

结果文件见：06.StatTest/Anosim/stat_anosim.txt

**MRPP分析**

MRPP分析与Anosim类似，用于分析组间微生物群落结构的差异是否显著，通常配合PCA、PCoA、NMDS等降维图使用，详细计算过程可查看R软件的说明。分析结果见下表。

**表3.12 MRPP分析结果**

| Group | A | observed-delta | expected-delta | Significance |
|---|---|---|---|---|
| IT-VS-C | -0.00661 | 0.676709 | 0.672262 | 0.6 |
| H-VS-C | 0.109420 | 0.623805 | 0.700448 | 0.1 |
| CHB-VS-C | -0.02591 | 0.666877 | 0.650028 | 0.6 |
| H-VS-IT | 0.085575 | 0.652961 | 0.714068 | 0.333333 |
| CHB-VS-IT | -0.04534 | 0.706801 | 0.676139 | 0.666666 |
| CHB-VS-H | 0.001060 | 0.640671 | 0.641351 | 0.666666 |

Observed delta 值越小说明组内差异小，Expected delta 值越大说明组间差异大。A值大于0说明组间差异大于组内差异，A值小于0说明组内差异大于组间差异。Significance值小于0.05说明差异显著

结果文件见：06.StatTest/MRPP/stat_mrpp.txt

# 4 分析说明以及参考文献

## 4.1 测序数据处理

根据Barcode序列和PCR扩增引物序列从下机数据中拆分出各样品数据，截去Barcode和引物序列后使用**PEAR**（V1.2.11）对每个样品的reads进行拼接，得到的拼接序列为原始Tags数据（Raw Tags）；拼接得到的Raw Tags，需要经过严格的过滤处理得到高质量的Tags数据（Clean Tags）。 参照**Qiime**（V1.9.1）的Tags质量控制流程， 进行如下操作：1）Tags截取：将Raw Tags从连续低质量值（默认质量阈值为<=19）碱基数达到设定长度（默认长度值为3）的第一个低质量碱基位点截断；2）Tags长度过滤：Tags经过截取后得到的Tags数据集，进一步过滤掉其中连续高质量碱基长度小于Tags长度75%的Tags。 经过以上处理后得到的Tags需要进行去除嵌合体序列（http://www.drive5.com/usearch/manual/chimera_formation.html）的处理， Tags序列通过**UCHIME Algorithm**与数据库**Gold database**进行比对 检测嵌合体序列， 并最终去除其中的嵌合体序列，得到最终的有效数据（Effective Tags）。

## 4.2 OTU聚类和物种注释

利用Uparse软件（**Uparse V8.1.1861**）对所有样品的全部 Effective Tags进行聚类，默认以97%的一致性（Identity）将序列聚类成为OTUs（Operational Taxonomic Units），同时会选取OTUs的代表性序列，依据其算法原则，筛选的是OTUs中出现频数最高的序列作为OTUs的代表序列。 对OTUs代表序列进行物种注释，用**uclust**方法与Silva数据库（http://www.arb-silva.de）进行物种注释分析，并分别在各个分类水平：kingdom（界），phylum（门），class（纲），order（目），family（科），genus（属），species（种）统计各样本的群落组成。 使用PyNAST软件（Version 1.2）与Silva数据库中的 "Core Set" 数据信息进行快速多序列比对，得到所有OTUs代表序列的系统发生关系。 最后对各样品的数据进行均一化处理，以样品中数据量最少的为标准进行均一化处理，后续的Alpha多样性分析和Beta多样性分析都是基于均一化处理后的数据。

## 4.3 样品复杂度分析（Alpha Diversity）

使用**Qiime**软件（Version 1.9.1）计算Observed-species，Chao1，Shannon，Simpson，ACE，Goods-coverage 指数，使用R软件（Version 3.2.2）绘制稀释曲线，Rank abundance曲线，物种累积曲线并使用R软件进行Alpha多样性指数组间差异分析；Alpha多样性指数组间差异分析会分别进行有参数检验和非参数检验，如果只有两组，选用T-test和wilcox检验，如果多于两组，选用的是Tukey检验和agricolae包的wilcox检验。

Alpha多样性指数具体描述如下:

1、计算菌群丰度（Community richness）的指数:

[Chao - the Chao1 estimator] (http://scikit-bio.org/docs/latest/generated/generated/skbio.diversity.alpha.chao1.html#skbio.diversity.alpha.chao1);

[ACE - the ACE estimator] (http://scikit-bio.org/docs/latest/generated/generated/skbio.diversity.alpha.ace.html#skbio.diversity.alpha.ace);

2、计算菌群多样性（Community diversity）的指数:

[Shannon - the Shannon index] (http://scikit-bio.org/docs/latest/generated/generated/skbio.diversity.alpha.shannon.html#skbio.diversity.alpha.shannon);

[Simpson - the Simpson index] (http://scikit-bio.org/docs/latest/generated/generated/skbio.diversity.alpha.simpson.html#skbio.diversity.alpha.simpson);

3、测序深度指数有:

[Coverage - the Good’s coverage](http://scikit-bio.org/docs/latest/generated/generated/skbio.diversity.alpha.goods_coverage.html#skbio.diversity.alpha.goods_coverage);

## 4.4 多样品比较分析（Beta Diversity）

用**Qiime**软件（Version 1.9.1）计算Unifrac距离、构建UPGMA样品聚类树。使用R件（Version 3.2.2）绘制PCA，PCoA和NMDS图。PCA分析使用R软件的ade4包和ggplot2软件包，PCoA分析使用R软件的WGCNA，stats和ggplot2软件包，NMDS分析使用R软件的vegan软件包。 使用R软件进行Beta多样性指数组间差异分析，分别进行有参数检验和非参数检验，如果只有两组，选用T-test和wilcox检验，如果多于两组，选用的是Tukey检验和agricolae包的wilcox检验。

## 4.5 统计分析（Statistics）

组间差异显著的物种分析利用R软件做组间T_test检验，得到p值，使用R软件的qvalue得到q值。Metastats分析使用R软件在各分类水平（Phylum、Class、Order、Family、Genus、Species）下，做组间的permutation test，得到p值，然后利用Benjamini and Hochberg False Discovery Rate方法对于p值

进行修正，得到FDR值。Anosim分析和MRPP分析分别使用R vegan包的anosim函数和mrpp函数。LEfSe分析使用LEfSe（**LEfSe**）软件，默认设置LDA Score的筛选值为2。首先使用非参数系数的Kruskal-Wallis (KW) sum-rank test检测组间丰度显著差异的特征，如果组间有相关联的子分组，则再进一步使用Wilcoxon rank-sum test对上一步的差异特征在子分组中的差异一致性检查，最后运用LDA判别分析估计这些差异特征对组间区别的影响大小。

分析方法英文版Methods.pdf

主要参考文献如下：


[1] Caporaso, J. Gregory, et al. Global patterns of 16S rRNA diversity at a depth of millions of sequences per sample. Proceedings of the National Academy of Sciences 108.Supplement 1 (2011): 4516-4522.

[2] Youssef, Noha, et al. Comparison of species richness estimates obtained using nearly complete fragments and simulated pyrosequencing-generated fragments in 16S rRNA gene-based environmental surveys. Applied and environmental microbiology 75.16 (2009): 5227-5236.

[3] Hess, Matthias, et al. Metagenomic discovery of biomass-degrading genes and genomes from cow rumen. Science 331.6016 (2011): 463-467.

[4] Li, Bing, et al. Characterization of tetracycline resistant bacterial community in saline activated sludge using batch stress incubation with high-throughput sequencing analysis. Water research 47.13 (2013): 4207-4216.

[5] Ondov, Brian D., Nicholas H. Bergman, and Adam M. Phillippy. Interactive metagenomic visualization in a Web browser. BMC bioinformatics 12.1 (2011): 385.

[6] Whittaker, Robert H. Evolution and measurement of species diversity. Taxon (1972): 213-251.

[7] Lundberg, Derek S., et al. Practical innovations for high-throughput amplicon sequencing.Nature methods 10.10 (2013): 999-1002.

[8] Lozupone, Catherine, and Rob Knight. UniFrac: a new phylogenetic method for comparing microbial communities. Applied and environmental microbiology 71.12 (2005): 8228-8235.

[9] Lozupone, Catherine, et al. UniFrac: an effective distance metric for microbial community comparison. The ISME journal 5.2 (2011): 169.

[10] Lozupone, Catherine A., et al. Quantitative and qualitative β diversity measures lead to different insights into factors that structure microbial communities. Applied and environmental microbiology 73.5 (2007): 1576-1585.

[11] Avershina, Ekaterina, Trine Frisli, and Knut Rudi. De novo Semi-alignment of 16S rRNA Gene Sequences for Deep Phylogenetic Characterization of Next Generation Sequencing Data. Microbes and Environments 28.2 (2013): 211-216.

[12] Magali Noval Rivas, PhD, Oliver T. Burton, et al. A microbita signature associated with experimental food allergy promotes allergic senitization and anaphylaxis. The Journal of Allergy and Clinical Immunology.Volume 131, Issue 1, Pages 201-212, January 2013.

[13] Zhang J, Kobert K, Flouri T, Stamatakis A. PEAR: a fast and accurate Illumina Paired-End reAd mergeR. Bioinformatics. 2014;30(5):614-620.

[14] Bokulich, Nicholas A., et al. Quality-filtering vastly improves diversity estimates from Illumina amplicon sequencing. Nature methods 10.1 (2013): 57-59.

[15] Caporaso, J. Gregory, et al. QIIME allows analysis of high-throughput community sequencing data. Nature methods 7.5 (2010): 335-336.

[16] Edgar, Robert C., et al. UCHIME improves sensitivity and speed of chimera detection. Bioinformatics 27.16 (2011): 2194-2200.

[17] Haas, Brian J., et al. Chimeric 16S rRNA sequence formation and detection in Sanger and 454-pyrosequenced PCR amplicons. Genome research 21.3 (2011): 494-504.

[18] Edgar, Robert C. UPARSE: highly accurate OTU sequences from microbial amplicon reads. Nature methods 10.10 (2013): 996-998.

[19] Wang, Qiong, et al. Naive Bayesian classifier for rapid assignment of rRNA sequences into the new bacterial taxonomy. Applied and environmental microbiology 73.16 (2007): 5261-5267.

[20] DeSantis, Todd Z., et al. Greengenes, a chimera-checked 16S rRNA gene database and workbench compatible with ARB. Applied and environmental microbiology 72.7 (2006): 5069-5072.

[21] Caporaso, J. Gregory, et al. PyNAST: a flexible tool for aligning sequences to a template alignment. Bioinformatics 26.2 (2010): 266-267.

[22] White, James Robert, Niranjan Nagarajan, and Mihai Pop. Statistical methods for detecting differentially abundant features in clinical metagenomic samples. PLoS computational biology 5.4 (2009): e1000352.
