# Supplementary material for: Enteral nutrition modulation with n-3 PUFAs directs microbiome and lipid metabolism in mice
Source: PLoS One. 2021 Mar 25;16(3):e0248482. doi: 10.1371/journal.pone.0248482 (PMC7993877; doi:10.1371/journal.pone.0248482)
Supplement: S3 File — (PDF) [file pone.0248482.s004.pdf]

报告编号：20191201

技术服务报告

项目名称：胆汁酸检测分析

单位名称：浙江大学

联　系　人：吴漫漫

联系方式：17857685283

电子邮件：

杭州汉库医学检验实验室

2019.12.06

肝组织样本及粪便样本中 15 种胆汁酸含量的定量检测

1. 仪器及试剂

1.1 试剂

乙腈、甲醇（色谱纯）购自 Merk 公司，甲酸（分析纯）、乙酸铵（分析纯）购自 CNW，超纯水由 CLV-Toc/UF 超纯水仪（Thermo）制备。

标准品：胆酸（CA）、脱氧胆酸（DCA）、甘氨胆酸钠盐（GCA）、甘氨熊脱氧胆酸（GUDCA）、甘氨鹅脱氧胆酸钠盐（GCDCA）、牛磺胆酸钠盐（TCA）、牛磺脱氧胆酸钠盐（TDCA）、甘氨石胆酸（GLCA）、熊脱氧胆酸（UDCA）、牛磺石胆酸（TLCA）购自加拿大 TRC 公司；鹅脱氧胆酸（CDCA）、石胆酸（LCA）、甘氨脱氧胆酸钠盐（GDCA）、牛磺熊脱氧胆酸钠盐（TUDCA）、牛磺鹅脱氧胆酸钠盐（TCDCA）购自 Sigma-Aldrich；

同位素标准品：CA-d4、UDCA-d4、CDCA-d4 购自 CDN（加拿大），其余的同位素内标购自 TRC（加拿大）。

1.2 设备

超高效液相色谱-三重四极杆质谱联用仪（UPLC/MS/MS）Acquity UPLC®I CLASS XEVO® TQ-S，配有电喷雾（ESI）离子源及 Masslynx 工作站系统，美国 Waters 公司；Legend micro 17R 低温高速离心机，美国 Thermo Scientific 公司；VORTEX3 S025 涡旋混合器，德国 IKA 公司。

2. 试剂的制备

内标溶液配制：用甲醇配制 1 mg/mL 的 15 种胆汁酸同位素内标储备液，-20℃保存；取适量储备液用甲醇稀释成 0.5 μmol/L 的内标沉淀剂，置-4℃条件下保存。

标准溶液配制：15 种胆汁酸标准品用甲醇配制成 1mg/mL 的标准品储备液，-20℃保存。分别取适量标准品储备液，稀释成标准曲线绘制所需的系列浓度。

碳酸氢铵溶液：配制成 20 mmol/L 的水溶液。

3. 样本制备

3.1 肝组织样本处理

准确称取组织样本约 20 mg（湿重，记录），放入冷冻碾磨仪（球磨仪）中，加 500 μL 内标沉淀剂（4℃）和 500 μL 冷甲醇，碾磨 1 min，完全转移至 1.5 mL 离心管中，在 4℃条件下以 13000 rpm 离心 10 min，取 800 μL 上清液至新的离心管中，置于-20℃保存。

3.2 粪便样本处理

粪便搅拌均匀，冻干后碾成粉末，准确称量约 100 mg，加入 200 μL 碳酸氢铵溶液。静置 5 min 后，

加入 500 μL 内标沉淀剂（4℃）及 500μL 冷甲醇，涡旋混匀，超声萃取 2min，在 4℃条件下以 13000 rpm 离心 10 min，取 800 μL 上清液至新的离心管中，置于-20℃保存。

3.3 进样前处理

将上述样本取出，恢复到室温，准确移取 100 μL 样本处理液，加入 100 μL 纯水，涡旋混匀，离心后取上清液至进样瓶中待测。

3.4 标准曲线样本制备

取 100 μL 标准溶液加入 300 μL 沉淀剂，涡旋混匀，离心后取 100 μL 上清，加入 100 μL 纯水，涡旋混匀，离心后取上清液至进样瓶中待测。

4. UPLC-MS/MS 分析

色谱条件：色谱柱选用 Acquity UPLC BEH C18（2.1×100 mm，1.8 μm）（Waters）；进样量：10 μL；流速：0.4mL/min；柱温：40℃；流动相 A 为 10 mmol/L 乙酸铵水溶液，流动相 B 为 0.1%甲酸/甲醇溶液；梯度洗脱条件：起始流动相为 35%B，3 min 升至 60%B，4.5 min 升至 100%B 保持 0.5 分钟，后复原至 35%B 平衡 1 分钟。

质谱条件：ESI 正离子模式，毛细管电压 2500 V，脱溶剂温度 450℃，锥孔电压 30 V，脱溶剂气 700 L/Hr，锥孔气为 30，离子源温度 150℃。采用 MRM 模式，每个化合物两个离子对，分别用于定量和定性。

定量分析：采用内标法进行定量，通过体积校准和重量较准，获得化合物在粪便和组织中的含量。

5. 分析结果

5.1 分析方法评估

15 种胆汁酸标准溶液检测结果显示，在 0.032~100 μmol/L 浓度范围内，线性相关系数均大于 0.995。

质控样本的检测结果表明，15 种胆汁酸检测结果的 RSD 均小于 15%，同时质控样本的实际定值和日常质控数值的偏差均小于 15%，表明测试结果稳定可控。

5.2 实际数据分析

根据定量曲线，结合体积校准，计算样本中胆汁酸组分含量检测结果如下表：

表 1 样本中胆汁酸组分绝对含量检测结果 （含量单位 pmol）

	重量(mg)	TCA	TUDCA	TCDCA	TDCA	TLCA	GCA	GUDCA	GCDCA	GDCA	GLCA	CA	UDCA	CDCA	DCA	LCA
G-1	20.7	1681.05	94.94	239.19	144.31	2.88	368.36	3.62	6.76	3.58	0.30	145.40	3.75	3.10	3.06	0.11
G-2	20.7	1011.32	170.56	258.96	275.57	4.62	136.26	3.24	3.88	4.71	0.14	17.25	1.06	1.05	1.55	0.01
G-3	19.6	1311.69	250.47	358.60	219.92	11.86	134.79	3.60	7.16	4.98	0.16	6.70	0.71	1.30	1.12	0.03
G-4	21.2	6504.68	526.23	377.25	391.05	8.01	2802.82	72.21	66.45	90.52	0.55	76.82	14.29	3.82	84.84	2.19
G-5	20.7	11065.14	121.02	249.36	267.09	11.97	3441.76	15.01	39.65	71.99	1.75	385.71	6.43	5.08	19.66	2.36
G-6	21	7315.24	709.53	839.68	174.90	28.63	11522.78	345.68	593.29	140.95	6.65	277.10	61.68	32.02	14.55	3.47
G-7	20.8	5914.13	143.69	207.44	142.91	6.14	5155.41	47.61	83.24	76.04	1.14	171.08	7.61	4.46	6.69	1.39
G-8	21.5	9754.56	326.20	423.33	170.46	4.01	4188.48	31.83	65.50	25.66	0.40	1720.33	35.65	26.97	21.98	1.77
G-9	20.8	6376.71	140.07	298.69	44.56	3.32	4346.98	30.41	102.00	19.22	0.85	140.82	3.85	4.16	2.94	2.53
G-10	20.2	5938.04	252.93	461.09	74.11	3.53	2616.80	47.27	106.37	28.95	0.46	277.86	27.11	22.75	5.44	1.24
G-11	19.9	6805.64	306.41	288.57	149.02	9.68	1044.05	22.72	30.64	20.16	0.76	845.99	85.99	32.27	11.08	2.32
G-12	20.3	6196.15	110.29	612.44	247.41	39.60	8274.23	67.44	491.44	184.02	10.97	320.47	16.25	25.21	11.84	4.94
G-13	20	4442.79	144.01	360.44	91.80	12.33	3152.68	43.99	154.76	57.59	2.83	194.01	10.30	16.42	5.61	1.90
F-1	101.8	64.01	30.37	208.13	26.19	12.63	27.87	4.95	21.01	17.77	7.85	1046.40	25065.73	1255.15	13524.73	5971.39
F-2	54.4	381.57	167.22	75.12	71.79	3.17	187.09	23.62	8.91	28.58	1.97	1444.88	29132.78	93.33	17613.29	3749.23
F-3	104.7	112.56	101.38	31.34	58.48	10.14	179.69	71.58	12.72	88.46	13.06	1949.99	40785.50	1155.17	24665.75	5690.31
F-4	105.8	222.89	38.09	53.25	73.46	1.57	52.53	14.60	4.74	109.97	2.57	9556.07	34823.54	1244.90	30404.11	3639.27
F-5	101.9	92.23	25.13	58.03	48.74	4.19	90.93	11.93	31.12	51.85	4.66	6014.53	25930.06	1423.23	24057.43	4926.52
F-6	99.7	497.11	107.28	34.23	237.46	11.18	8.05	2.14	0.46	26.29	2.04	8223.92	15837.48	289.80	17771.41	1980.21
F-7	81.3	542.67	145.00	62.26	404.49	25.89	9.66	7.56	0.50	56.95	5.89	4231.23	14453.94	144.71	14919.33	1362.19
F-8	56.2	4993.87	162.93	117.59	11.89	0.80	114.30	1.78	0.67	36.67	0.31	16986.42	3890.93	2762.94	159.98	217.56
F-9	60.9	4588.35	314.18	142.91	411.06	10.61	122.65	2.28	4.25	36.39	0.57	14856.78	4452.45	1976.68	8243.20	707.56
F-10	74.5	26731.56	111.73	72.52	9.68	1.33	451.26	6.46	3.08	25.19	0.22	16444.52	7413.57	3003.69	71.34	197.32
F-11	49.5	3980.48	92.61	52.42	18.08	1.07	76.13	1.54	1.65	13.25	0.37	12735.00	2460.26	1144.67	581.06	234.23
F-12	64.6	5451.85	213.29	160.30	4.60	1.62	106.67	2.53	4.79	22.71	0.30	16199.08	4588.30	3570.51	40.23	214.83
F-13	56.6	10779.76	154.57	97.92	9.79	0.90	169.33	2.58	2.51	17.01	0.33	14870.00	3735.99	1793.13	65.29	184.23

表 2 样本中胆汁酸组分浓度检测结果 （含量单位 nmol/g）

	TCA	TUDCA	TCDCA	TDCA	TLCA	GCA	GUDCA	GCDCA	GDCA	GLCA	CA	UDCA	CDCA	DCA	LCA
G-1	81.210	4.586	11.555	6.971	0.139	17.795	0.175	0.327	0.173	0.014	7.024	0.181	0.150	0.148	0.005
G-2	48.856	8.240	12.510	13.313	0.223	6.583	0.157	0.187	0.228	0.007	0.833	0.051	0.051	0.075	0.000
G-3	66.923	12.779	18.296	11.220	0.605	6.877	0.184	0.365	0.254	0.008	0.342	0.036	0.066	0.057	0.002
G-4	306.825	24.822	17.795	18.446	0.378	132.208	3.406	3.134	4.270	0.026	3.624	0.674	0.180	4.002	0.103
G-5	534.548	5.846	12.046	12.903	0.578	166.269	0.725	1.915	3.478	0.085	18.633	0.311	0.245	0.950	0.114
G-6	348.345	33.787	39.985	8.329	1.363	548.704	16.461	28.252	6.712	0.317	13.195	2.937	1.525	0.693	0.165
G-7	284.333	6.908	9.973	6.871	0.295	247.856	2.289	4.002	3.656	0.055	8.225	0.366	0.214	0.322	0.067
G-8	453.700	15.172	19.690	7.928	0.187	194.813	1.480	3.047	1.193	0.019	80.015	1.658	1.254	1.022	0.082
G-9	306.573	6.734	14.360	2.142	0.160	208.989	1.462	4.904	0.924	0.041	6.770	0.185	0.200	0.141	0.122
G-10	293.962	12.521	22.826	3.669	0.175	129.545	2.340	5.266	1.433	0.023	13.755	1.342	1.126	0.269	0.061
G-11	341.992	15.397	14.501	7.488	0.486	52.465	1.142	1.540	1.013	0.038	42.512	4.321	1.622	0.557	0.117
G-12	305.229	5.433	30.169	12.188	1.951	407.598	3.322	24.209	9.065	0.540	15.787	0.800	1.242	0.583	0.243
G-13	222.140	7.201	18.022	4.590	0.617	157.634	2.200	7.738	2.880	0.142	9.701	0.515	0.821	0.281	0.095
F-1	0.629	0.298	2.044	0.257	0.124	0.274	0.049	0.206	0.175	0.077	10.279	246.225	12.330	132.856	58.658
F-2	7.014	3.074	1.381	1.320	0.058	3.439	0.434	0.164	0.525	0.036	26.560	535.529	1.716	323.774	68.920
F-3	1.075	0.968	0.299	0.559	0.097	1.716	0.684	0.121	0.845	0.125	18.625	389.546	11.033	235.585	54.349
F-4	2.107	0.360	0.503	0.694	0.015	0.497	0.138	0.045	1.039	0.024	90.322	329.145	11.767	287.373	34.398
F-5	0.905	0.247	0.569	0.478	0.041	0.892	0.117	0.305	0.509	0.046	59.024	254.466	13.967	236.089	48.347
F-6	4.986	1.076	0.343	2.382	0.112	0.081	0.021	0.005	0.264	0.020	82.487	158.851	2.907	178.249	19.862
F-7	6.675	1.784	0.766	4.975	0.318	0.119	0.093	0.006	0.700	0.072	52.045	177.785	1.780	183.510	16.755
F-8	88.859	2.899	2.092	0.212	0.014	2.034	0.032	0.012	0.652	0.006	302.249	69.234	49.163	2.847	3.871
F-9	75.342	5.159	2.347	6.750	0.174	2.014	0.037	0.070	0.598	0.009	243.954	73.111	32.458	135.356	11.618
F-10	358.813	1.500	0.973	0.130	0.018	6.057	0.087	0.041	0.338	0.003	220.732	99.511	40.318	0.958	2.649
F-11	80.414	1.871	1.059	0.365	0.022	1.538	0.031	0.033	0.268	0.007	257.273	49.702	23.125	11.739	4.732
F-12	84.394	3.302	2.481	0.071	0.025	1.651	0.039	0.074	0.352	0.005	250.760	71.026	55.271	0.623	3.326
F-13	190.455	2.731	1.730	0.173	0.016	2.992	0.046	0.044	0.301	0.006	262.721	66.007	31.681	1.154	3.255

免责声明

1. 测试结果仅对所送检的样品负责。本项目中的样本采集、分装、预处理和邮寄均由测试委托单位（浙江大学）完成，由此导致的结果不确定性由委托单位负责。
2. 委托单位应对送检样品的代表性、样品信息等资料的真实性负责，本实验室不承担任何相关责任。
3. 分析检测样本及检测原始数据将在本实验室保留 30 天。如委托单位对分析结果有异议，需在 15 日自然日内以书面方式与本实验室取得联系。超过 15 个自然日，将视为委托单位对本报告内容和检测结果无疑义。超过 30 个自然日，本实验室有权按照标准规程自行废弃标本和删除原始数据。
4. 本测试结果仅用于科学研究，包括数据分析、性能评价和质量分析等。如有其他用途，须与本实验室沟通确认。本实验室不承担任何未经确认的测试结果使用所导致的直接和/或间接经济损失、法律责任。
5. 测试结果以本报告提供的数据为准。本实验室不承担任何因涂改、篡改、冒用等不良行为所导致的经济损失、法律责任。
6. 杭州汉库医学检验实验室的联系方式如下：

   联系人：金钱星；移动电话：15925661166；E-mail：jqx@hankoo.cn。
